# Comprehensive enhancer-target gene assignments improve gene set level interpretation of genome-wide regulatory data

**DOI:** 10.1186/s13059-022-02668-0

**Published:** 2022-04-26

**Authors:** Tingting Qin, Christopher Lee, Shiting Li, Raymond G. Cavalcante, Peter Orchard, Heming Yao, Hanrui Zhang, Shuze Wang, Snehal Patil, Alan P. Boyle, Maureen A. Sartor

**Affiliations:** 1 Department of Computational Medicine and Bioinformatics, University of Michigan Medical School, Ann Arbor, MI USA; 2 Department of Biostatistics, School of Public Health, University of Michigan Medical School, Ann Arbor, MI USA; 3 Biomedical Research Core Facilities, Epigenomics Core, University of Michigan Medical School, Ann Arbor, MI USA; 4 Department of Human Genetics, University of Michigan Medical School, Ann Arbor, MI USA

**Keywords:** Enhancer, Target gene, Gene Ontology, Gene set enrichment test, Genomic regions, ChIP-seq, Regulome

## Abstract

**Background:**

Revealing the gene targets of distal regulatory elements is challenging yet critical for interpreting regulome data. Experiment-derived enhancer-gene links are restricted to a small set of enhancers and/or cell types, while the accuracy of genome-wide approaches remains elusive due to the lack of a systematic evaluation. We combined multiple spatial and in silico approaches for defining enhancer locations and linking them to their target genes aggregated across >500 cell types, generating 1860 human genome-wide distal enhancer-to-target gene definitions (EnTDefs). To evaluate performance, we used gene set enrichment (GSE) testing on 87 independent ENCODE ChIP-seq datasets of 34 transcription factors (TFs) and assessed concordance of results with known TF Gene Ontology annotations, and other benchmarks.

**Results:**

The top ranked 741 (40%) EnTDefs significantly outperform the common, naïve approach of linking distal regions to the nearest genes, and the top 10 EnTDefs perform well when applied to ChIP-seq data of other cell types. The GSE-based ranking of EnTDefs is highly concordant with ranking based on overlap with curated benchmarks of enhancer-gene interactions. Both our top general EnTDef and cell-type-specific EnTDefs significantly outperform seven independent computational and experiment-based enhancer-gene pair datasets. We show that using our top EnTDefs for GSE with either genome-wide DNA methylation or ATAC-seq data is able to better recapitulate the biological processes changed in gene expression data performed in parallel for the same experiment than our lower-ranked EnTDefs.

**Conclusions:**

Our findings illustrate the power of our approach to provide genome-wide interpretation regardless of cell type.

**Supplementary Information:**

The online version contains supplementary material available at 10.1186/s13059-022-02668-0.

## Background

Enhancers, silencers, and insulators are key genomic cis-regulatory elements that play pivotal roles in spatiotemporal control of gene expression by physical contact with the promoters of target genes they control [1–3]. Promoters are located immediately upstream of the transcription start sties (TSSs), facilitating the recruitment of transcription factors and RNA polymerase II (RNAPII) to instruct the initiation and direction of gene transcription, whereas enhancers and silencers can be located anywhere in the genome and often at distal regions, such as upstream, downstream, or in introns of target genes or unrelated genes. Via interaction with promoters of the target genes, enhancers are bound by activator proteins and stimulate the rate of transcription, while silencers were bound by repressor proteins and decrease the rate. In certain cases when the interactions between enhancers/silencers and promoters are unwanted, insulators can block their interactions [4]. Bound by tissue-specific transcription factors and cofactors, such as p300 and Mediator, the cis-regulatory elements and promoter connections direct what, when, and how the genome is transcribed so as to control cell fate decisions during development and differentiation [5–7]. For simplicity, we will refer to these distal cis-regulatory elements as general “enhancers” (>5 kb from a transcription start site [TSS]) hereinafter.

Perturbation of enhancer activities and/or functions induced by genomic variants, epigenomic dysregulation, and/or aberrant chromosomal rearrangements can underlie disease susceptibility and developmental malformations [8, 9]. A prototypic example of this is the point mutation in the *Shh* enhancer, ZRS (*zone of polarizing activity regulatory sequence*), which can lead to limb malformations such as polydactyly in humans [10]. Recently, genome-wide association studies (GWAS) identified that >88% of disease-linked variants occur within noncoding regulatory DNA [11], especially enriched in enhancers [12]. These findings confirm the importance of enhancers in orchestrating transcriptional regulation and reveal that the dysregulation of enhancer function contributes to the pathogenesis of a variety of diseases, referred to as “enhanceropathies” [13].

A challenge in enhancer biology is to decipher their target genes and the mechanisms underlying the precise enhancer-gene interactions, which is reviewed in Pennacchio et al. [14]. The enhancer to target gene specificity is essential to understand how gene expression is programmed during normal development and differentiation, and how the ectopic enhancer and/or non-target gene interactions can lead to diseases. However, interpreting genome-wide regulatory data is significantly hampered by our limited knowledge of enhancers and their target genes for multiple reasons. First, enhancers are commonly located distal to their target genes with multiple intervening genes in between, and greatly varying distances. One enhancer can act on multiple genes and one gene can be regulated by multiple enhancers [15]. Second, enhancers act in a dynamic and often cell-type-specific manner, which further complicates the definition of a comprehensive set of enhancers and their target genes. Third, enhancers and promoters share various characteristics and functions [16, 17], thus making it challenging to disentangle the two elements based on functional genomic data.

With the breathtaking progress in technologies such as massive parallel sequencing and high-resolution chromosome conformation capture, our knowledge of cis-regulatory elements’ function and spatial organization have grown considerably over the past decade [18–23]. In most cases, enhancers are located at regions distal of their target genes up to hundreds of kilobases, and they can bypass more proximally located genes to bind to the promoters of the genes they control through long-range 3D chromosomal interactions [19, 24, 25]. The 3D genome is organized in hierarchical layers, from bottom to top including chromatin loops (or insulated neighborhoods), topological associating domains (TADs), and compartments [26]. The chromatin loops are the fundamental structural and functional building blocks of genome organization, which form between two convergent CTCF (CCCTC binding factor) binding sites bound by the cohesin protein complex [27].

Large epigenomics consortia like ENCODE [28–30] and Roadmap Epigenomics [31], have generated a tremendous amount of regulatory data across various tissue and cell types, including genome-wide transcription factor (TF) binding by ChIP-seq [32], chromatin accessibility assays (e.g., DNase-seq [33], ATAC-seq [34]), genome-wide chromatin mark profiles, and 3D chromosome organization. However, enhancer-promoter interactions are still restricted to a small number of cell types, which have been probed by Chromatin Interaction Analysis by Paired-End Tag Sequencing (ChIA-PET [35]), and the genome-wide interaction map is still limited due to the high cost of Hi-C experiments [36]. Other enhancer-promoter interaction datasets have been generated by mathematical and/or bioinformatic approaches. The FANTOM5 [37] dataset is based on the gene expression correlation between enhancer and promoter regions, and Thurman et al. exploited DNase signal correlation between enhancers and promoters using DNase-seq data [38]. However, the reliability and generalization of these approaches remains elusive due to the lack of a systematic evaluation.

Gene set enrichment (GSE) testing is widely applied to infer the regulatory networks embedded in the abundant high-throughput gene regulation data, including ChIP-seq, Bisulfite sequencing, DNase-seq, and ATAC-seq. The first step in this analysis is to assign the genomic regions identified by the assays to their target genes, and most methods simply do the assignment using the nearest gene regardless of the actual regulatory targets [39–42]. Since enhancers and their target genes have long-range chromosomal contact, adjacent gene assignments tend to link enhancers to non-target genes, leading to incorrect interpretation for distal enhancer regulation. In this study, we aimed to determine the best sets of human “enhancers” (enhancers, silencers and insulators) and their gene targets. By all possible combinations of existing experimental and/or computationally derived datasets, we generated 1860 enhancer-to-target gene definitions, referred to as EnTDefs, and systematically evaluated their performance based on the concordance of GSE results of 87 ENCODE ChIP-seq datasets with known TF biological processes, resulting in a handful of best-performing EnTDefs. We also showed that as opposed to being random, target genes that are often missed or often falsely identified using adjacent gene assignments are biased to specific Gene Ontology terms. In addition, we compared cell-type-specific EnTDefs (CT-EnTDefs) with non-cell-type-specific ones (general EnTDefs) and found that general EnTDefs were more favorable. Our findings demonstrate that the novel, top-performing EnTDefs significantly enhance the biological interpretation for genomic region data regardless of cell type.

## Results

### Creation and ranking of genome-wide enhancer-to-target gene definitions (EnTDefs)

**Fig. 1:**
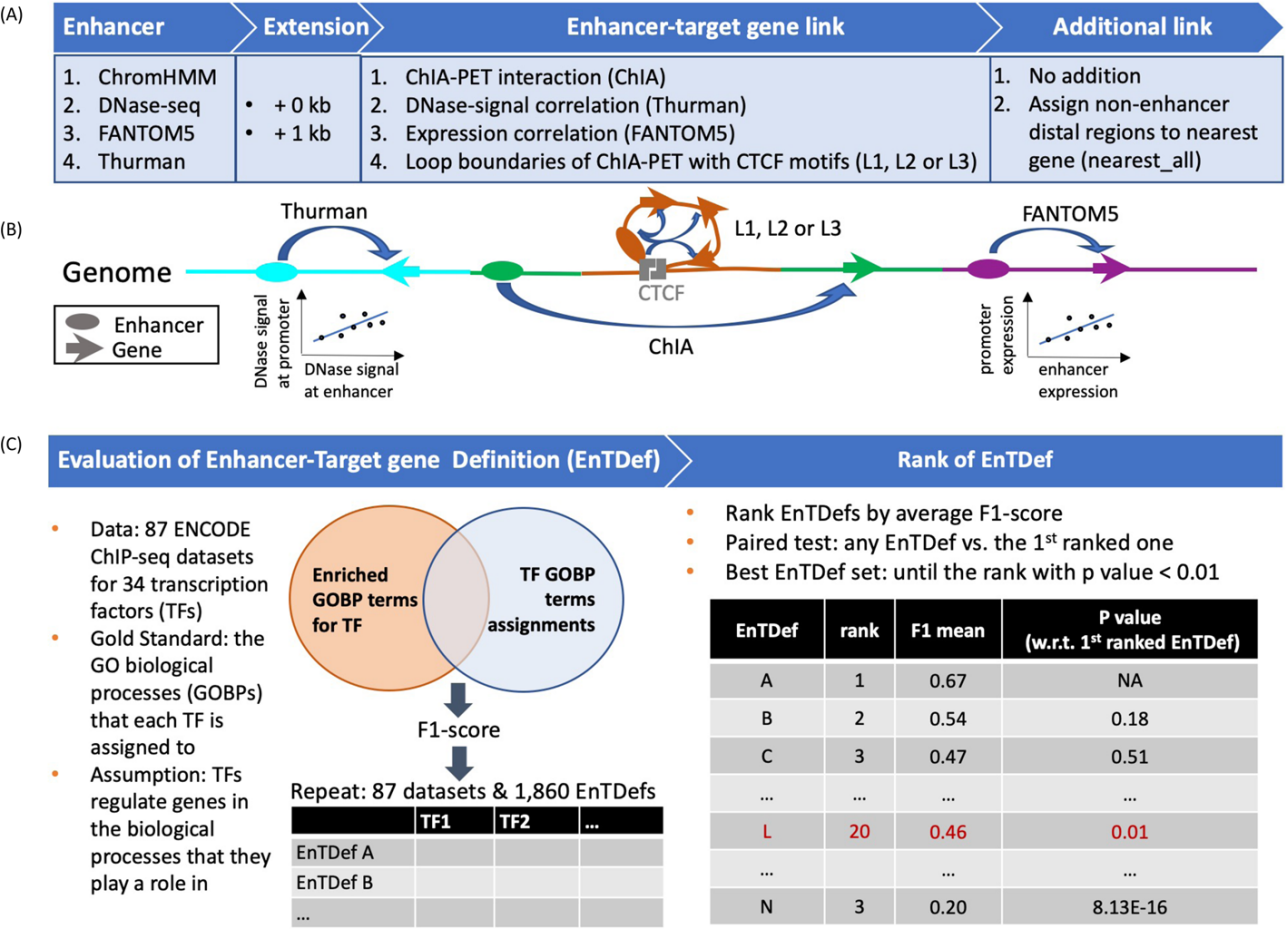
Workflow for generating and evaluating EnTDefs. **A** Enhancers were defined by ENCODE ChromHMM UCSC tracks, ENCODE DNase-seq hypersensitive sites (DHSs), Cap Analysis Gene Expression (CAGE) experiment-derived enhancers from the FANTOM5 project, and/or distal and non-promoter DHS within 500 kb of the correlated promoter DHSs from Thurman et al. **B** The enhancer-target gene links were defined by ChIA-PET interactions from ENCODE ChIA-PET data (ChIA), DNase signal correlation-based links from Thurman et al., expression correlation-based interactions from FANTOM5, and/or interactions between enhancers and genes within loop (L) boundaries of ChIA-PET with convergent CTCF motifs (L1 [one gene], L2 [≤ two gene], or L3 [≤ three genes] were allowed). An enhancer can be assigned to multiple genes. To increase the genome coverage, we allowed the extension of enhancers to 1 kb (i.e., enhancer extension), and assigned other regions outside of 5 kb from a TSS to the nearest gene (i.e., “nearest_All” additional links). All combinations of the above, allowing multiple at a time, defined the possible enhancer-to-target gene definitions (EnTDefs). **C** Left: 1860 EnTDefs were generated and GOBP GSE testing was performed on 87 ENCODE TF ChIP-seq datasets using each of the EnTDefs. By comparing the significant GOBP terms identified by GSE with each EntDef to those assigned to the TF by the GO database (“GO annotation”), the F1 score was calculated for each EnTDef-TF pair. Right: the EnTDefs were ranked by average F1 score across TFs in descending order. TF paired Wilcoxon sum-rank test was performed between the top ranked EnTDef and each of the sequential ones to identify the set of best EnTDefs (top 1 until the rank with *p*-value < 0.01)

Several approaches to define human enhancer locations and their target genes have been proposed in the literature, but no systematic study has been performed to evaluate their performance separately or in combination on a genome-wide scale. To determine the best sets of human enhancers and their distal gene targets, we generated a total of 1860 genome-wide Enhancer-Target gene Definitions (EnTDefs) using existing experiments and/or literature-derived data, and systematically evaluated their performance. This was done by applying all possible combinations of methods for defining (1) *enhancer region locations*, identified from four data sources (ChromHMM [43], DNase-seq [38], FANTOM5 [37, 44, 45], and Thurman [38]), and (2) *enhancer-target gene links*, defined by four different methods (ChIA-pet data [“ChIA”] [46, 47], DNAase-signal correlation [“Thurman”] [38], gene expression correlation [“FANTOM5”] [45], and loop boundaries with convergent CTCF motif [“L”] [48]), including combinations using multiple of each (see “14” for details). Overall, these included a total of 1,768,201 possible individual enhancer-target links across >500 cell types by integrating all of the 4 enhancer-defining datasets and all of the 4 enhancer-gene link datasets. These enhancer-target links were defined from 685,921 enhancers and 21,094 linked target genes. Figure 1 demonstrates the workflow for the creation and evaluation of these 1860 EnTDefs. For the “L” enhancer-gene linking method, we evaluated the loops with up to 3 genes (L1: one gene, L2: ≤ two genes, or L3: ≤ three genes), allowing the links between the enhancer to any of the included genes within the loop. Because current knowledge of enhancers is far from complete and the experimental data that assay enhancers to target genes is limited, the genome coverage of EnTDefs defined by the experimentally and/or computationally derived methods (Fig. 1A: four enhancer-defining methods and four enhancer-target gene linking methods) was expected to be low. Therefore, we extended the enhancer regions up to 1 kb and/or assigned regions outside of enhancers and promoters (within 5kb of a transcription start site (TSS)) to the gene with the nearest TSS (Fig. 1A: Extension and Additional link), resulting in 100% coverage of distal genomic regions (>5 kb of TSS). All of the 1860 EnTDefs were evaluated and ranked based on how well they performed in gene set enrichment (GSE) testing with genes’ distal ChIP-seq peaks. Specifically, the Gene Ontology biological process (GO BP) enrichment results from 87 ENCODE ChIP-seq datasets for 34 distinct transcription factors (TFs) were compared with the curated GO BP terms annotated to the same tested TFs (GO annotation by GO database) using F1 scores (see “14”). EnTDefs demonstrating higher concordance ranked higher, as they were better able to identify the known functions of the TFs based on their distal binding regions (non-promoters).

### Overview of the EnTDef characteristics

**Fig. 2:**
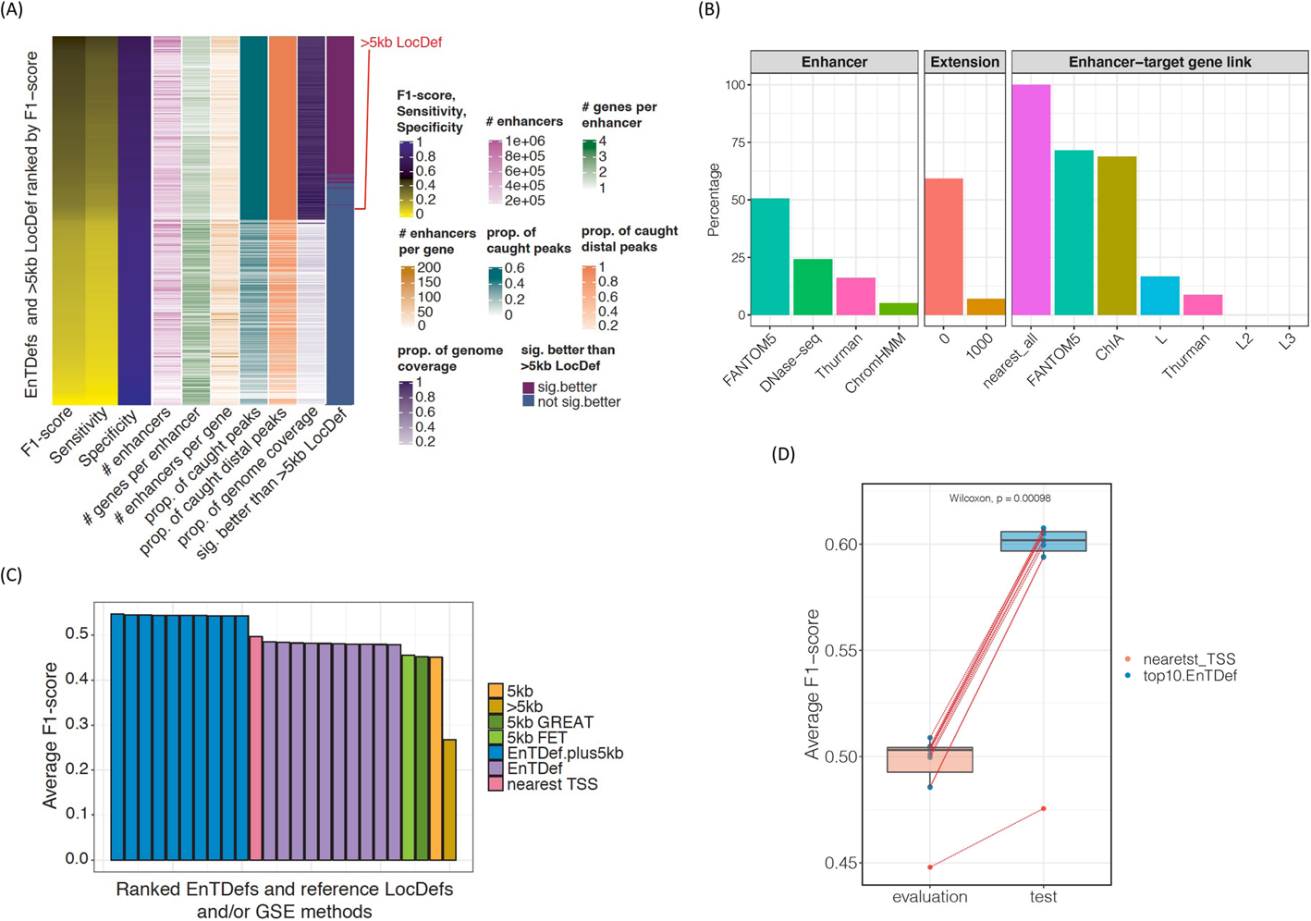
Characteristics of EnTDefs. **A** Overview of the characteristics of 1860 EnTDefs and >5 kb LocDef ranked by F1 score in descending order. F1 score, sensitivity, specificity, number of enhancers, average number of genes per enhancer, average number of enhancers per gene, average proportion of caught TF peaks, average proportion of caught TF peaks outside of 5 kb of TSSs, proportion of genome coverage, and whether the EnTDef was significantly better than the >5 kb LocDef are shown. **B** The percentage of the 741 EnTDefs with a particular method (four enhancer definition methods, with or without enhancer extension, seven enhancer-target gene link methods) that significantly outperformed the EnTDefs excluding that method only. For simplification, the “nearest_all” additional link method was grouped in enhancer-target gene link method. **C** Bar plot of average F1 scores for the top 10 EnTDefs plus 5 kb LocDef (blue bars), top 10 EnTDefs (purple bars), nearest TSS method (pink bar), >5 kb LocDef (mustard bar), and 5 kb LocDef used by PE.Approx (yellow bar), by GREAT (dark green bar) and by Fisher’s exact test (green bar). **D** Distribution of average F1 scores of the top 10 EnTDefs (blue dots) and the nearest_TSS (red dot) among *evaluation* ChIP-seq datasets or *test* ChIP-seq datasets. The dashed lines link the same EnTDefs used in the two different ChIP-seq datasets. The *p*-value of Wilcoxon signed-rank test was shown in the figure

We first investigated the characteristics of the 1860 EnTDefs by comparing them to simply assigning distal genomic regions (i.e., >5 kb from a TSS) to the genes with the nearest TSS (>5 kb Locus Definition [LocDef]) (Fig. 2A, Additional file 1: Fig. S1). The EnTDefs were ranked in decreasing order by their average F1 score across 34 TFs, and the top 741 EnTDefs (~40%) were found to significantly outperform the >5 kb LocDef (Wilcoxon signed-rank test, FDR < 0.05). The best-performing EnTDef (No. 1 ranked) was defined by DNase-seq plus FANTOM5 enhancers and ChIA, Thurman, and FANTOM5 enhancer-target gene link methods with the “nearest_All” addition. For the top 741 EnTDefs, the percentage of genome covered and percent of distal peaks caught (outside of 5 kb regions around TSSs) was as high as 100% (89–100%), the median number of genes assigned to each enhancer was 2 (range of 1–2), and the median number of enhancers assigned to each gene was 20 (range of 2–98). Out of the 741 EnTDefs, those ranked 2 through 19 were not significantly worse than the best-performing EnTDef (Wilcoxon signed-rank test, *p* > 0.01. Additional file 2: Table S1), suggesting that these 19 EnTDefs performed equally well. This finding was robust to the specific set of GO biological process (GOBP) annotations used (i.e., with or without IEA-based GO to gene annotations; see “14,” data not shown).

To assess the relative benefit of each method used for enhancer definition, enhancer extension, and enhancer-target gene link assignments, we compared the F1 scores of each of the top 741 EnTDefs containing a particular method to the F1 scores of other EnTDefs excluding that method only (keeping everything else the same). Using paired Wilcoxon tests, we calculated the percent of EnTDefs for each tested method that showed significantly increased F1 scores across the ChIP-seq datasets (Fig. 2B). Adding FANTOM5 significantly improved the performance of >50% of EnTDefs, whereas adding the ChromHMM method only enhanced the performance for ~5%. DNase-seq and Thurman methods ranked in the middle, improving ~24 and 16% of EnTDefs, respectively. EnTDefs without enhancer region extension significantly increased the F1 scores for ~60% of EnTDefs, while the ones with 1k bp extension only improved ~7% of EnTDefs. It is not surprising that all of the top 741 EnTDefs included the “nearest_all” addition, since this addition significantly increased genome coverage by assigning all regions outside enhancers and promoters to the closest gene (>5 kb LocDef), leading to improved sensitivity and thus F1 score (Fig. 2A). On the other hand, the fact that these 741 EnTDefs outperformed the >5 kb LocDef suggests that the “smart” enhancer to target gene assignments more accurately capture real biological regulatory elements for distal enhancer regions as compared to the simplistic assignment to nearest genes. FANTOM5 and ChIA enhancer-gene assignment methods significantly improved ~70% of EnTDefs, while the L and Thurman methods only improved ~1.7 and 9% of EnTDefs. Including more than one gene in the CTCT ChIA-PET loops (L2/L3 methods) failed to improve the performance. In addition, 70% of the 741 EnTDefs were generated using combinations of at least two different methods for enhancer definitions (ChromHMM, DNase-seq, FANTOM5, and/or Thurman) and 100% of them contained at least two enhancer-gene assignment methods (ChIA, FANTOM5, L, and/or Thurman), illustrating the importance of the integration of multiple data sources and methods to improve the performance of enhancer to target gene assignments.

### EnTDefs plus promoter regions outperform the nearest TSS method

Our analyses thus far have focused on the assessment of distal gene regulation. However, often the goal is to assess the functional regulation from anywhere in the genome, including binding both distal and proximal to TSSs. One commonly used method for ChIP-seq GSE testing is to link all peaks to the gene(s) with the nearest TSS, hereinafter referred to as the “nearest TSS” method (Additional file 1: Fig S1 “nearest TSS” LocDef), resulting in all peaks having at least one assigned gene. EnTDefs were generated for distal regions (outside the 5-kb windows around TSSs) and any regions within 5 kb of a TSS were ignored, whereas the “nearest TSS” method includes all genomic regions. Thus, to compare fairly with the “nearest TSS” method, we added promoter regions to the top 10 ranked EnTDefs, referred to as “EnTDef_plus5kb.” That is, peaks within 5 kb of a TSS were assigned to the nearest gene (Additional file 1: Fig. S1 “5 kb” LocDef), while distal peaks were assigned according to the EnTDef. All ten of the EnTDef_plus5kbs significantly outperformed the “nearest TSS” method (~0.05 increase in average F1 score, Wilcoxon signed-rank test, *p* < 0.0001) (Fig. 2C), using the same evaluation method based on F1 scores as used above (see “14”). Since the only difference between these is how distal binding events were defined, the improved performance of our EnTDef_plus5kbs is directly attributable to the distal enhancer-target gene links, and this comparison demonstrates that these distal links provide regulatory information beyond that provided by promoters and nearest genes.

We next determined if our “smart” EntDefs using only distal binding events could even outperform the use of all peaks (promoter and enhancer) with naïve assignments to the genes with the nearest TSS. When compared with the “nearest TSS” method, the top 10 best-performing EnTDefs showed slightly lower F1 scores (~0.03 lower), but the difference among the top half of them were not significantly different from “nearest TSS” (Wilcoxon signed-rank test, *p* > 0.05). Thus, although they did not outperform it, the best were not significantly worse. This illustrates the great importance of regulation from promoters in GSE testing.

Two other commonly used GSE methods for genomic regions, GREAT [39] and Fisher’s exact test (FET) using peaks within 5 kb of a TSS (Additional file 1: Fig. S1 “5kb” LocDef), were also evaluated using the same scheme. Notably, the three GSE testing methods (Poly-Enrich, GREAT and FET using 5 kb LocDef to assign peak to gene) performed equally well (Friedman test, *p* = 0.91), but significantly worse than the top 10 EnTDefs (distal regions only) (Fig. 2C, average F1 = 0.45 vs 0.47, Wilcoxon rank-sum test, *p* < 0.007). In addition, both the top 10 EnTDefs and 5 kb LocDef (i.e., assigning promoters to the nearest gene) significantly outperformed the >5 kb LocDef (i.e., the naïve approach of assigning distal regions to the nearest gene) (average F1 = 0.47, 0.45, vs 0.27, Wilcoxon signed-rank test, *p* = 2.37 × 10^−14^ and 1.32 × 10^−8^ respectively). In summary, although the naïve approach of linking distal regions to the nearest gene (>5 kb LocDef) did not outperform the use of promoter data only (5 kb LocDef), the use of distal binding events with “smart” gene assignments (EnTDefs) did outperform the use of promoter data only. Incorporation of these 5-kb promoter regions (5 kb LocDef) into the top 10 EnTDefs (“EnTDefs_plus5kb”) significantly further improves their performance (better than “nearest_TSS” approach), indicating both promoter and distal regions provide non-overlapping, independent evidence for regulatory programs. These findings illustrate the importance of accurately modeling regulation from enhancers and that when done well, enhancers have the potential to provide more regulatory information than promoters. We conclude that GSE testing using our top EnTDefs exceeds the commonly used nearest distance-based and promoter-only-based GSE approaches.

### Our EnTDefs are generalizable to different cell lines

Next, we sought to investigate whether the EnTDefs (which were selected based on their performance in GM12878, H1-HESC and K562 cell lines) can perform equally well testing ChIP-seq data from different cell lines (A549, HEPG2, HUVEC, and NB4). Surprisingly, the average F1 score in *test* ChIP-seq datasets (different cell lines) was significantly higher than that from the *evaluation* ChIP-seq datasets (original cell lines; average F1 = 0.59 vs. 0.50, Wilcoxon sum-rank test, *p* = 0.00098) (Fig. 2D, and Additional file 1: Fig. S2). This may be due to the *test* ChIP-seq datasets containing more peaks than the *evaluation* datasets (Additional file 1: Fig. S3A, Wilcoxon sum-rank test, *p* = 0.092), and indeed we found that the *F1 scores* were significantly correlated with the number of peaks (Additional file 1: Fig. S3B, Pearson’s correlation *r* = 0.65, *p* = 4.57 × 10^−6^). After correcting for the number of peaks, the association between the F1 score and dataset type was decreased, although *test* dataset F1 scores still remained higher than the *evaluation* F1 scores (*p* < 0.05; linear model with log_10_ peak number as covariate). Furthermore, the average F1 scores of the top10 EnTDefs and nearest_TSS in the *evaluation* dataset were strongly correlated with those in the *test* dataset (*r* = 0.94, *p* = 1.23 × 10^−5^), illustrating that an important variable in determining F1 score is the TF and/or antibody. The findings indicate that the performance of the top selected EnTDefs are independent of the cell types of ChIP-seq datasets, but likely strongly influenced by the quality of the datasets themselves (e.g., the specificity and efficiency of an antibody, ChIP quality, number of peaks). We reasoned that the EnTDefs were created based on the combinations of diverse data sources stemming from >500 different cell types, resulting in a consensus set of enhancer and gene assignments across various cell types, and therefore representative of the background interactions between enhancer and target genes across many cell types. The high generalizability of our top EnTDef makes it feasible to integrate with GSE testing in a cell-type-independent manner.

In addition, we applied our EnTDefs on a completely independent set of ChIP-seq experiments in GSE testing and evaluated their performance using a different metric. That is, we used data that are both from completely different, non-overlapping transcription factors (TFs) and completely different, non-overlapping cell types. The new datasets include 31 ENCODE ChIP-seq experiments of 9 cell lines and 14 transcription factors (TFs) (Additional file 2: Table S2), which all passed quality controls according to the Cistrome project (http://cistrome.org/db/#/about). The receiver operating characteristic (ROC) and precision-recall (PR) curves were generated for each ChIP-seq dataset when comparing the significant GOBP terms with the assigned ones for the tested TF (see “16”) at a series of GSE *p*-value cutoffs, and the area under PRC (AUPRC) and area under ROC (AUROC) were calculated. As compared to the baseline methods (nearest TSS and >5 kb), the top10 EnTDefs with or without plus 5 kb locus definition had both higher overall AUPRC and AUROC across the 31 ChIP-seq datasets (Additional file 1: Fig. S3C). This provides independent evidence of the outperformance of our EnTDef compared to the commonly used “nearest TSS” method, illustrating the robustness of our top EnTDefs across a broad range of datasets.

### General EnTDefs perform comparably to cell-type-specific EnTDefs

**Fig. 3:**
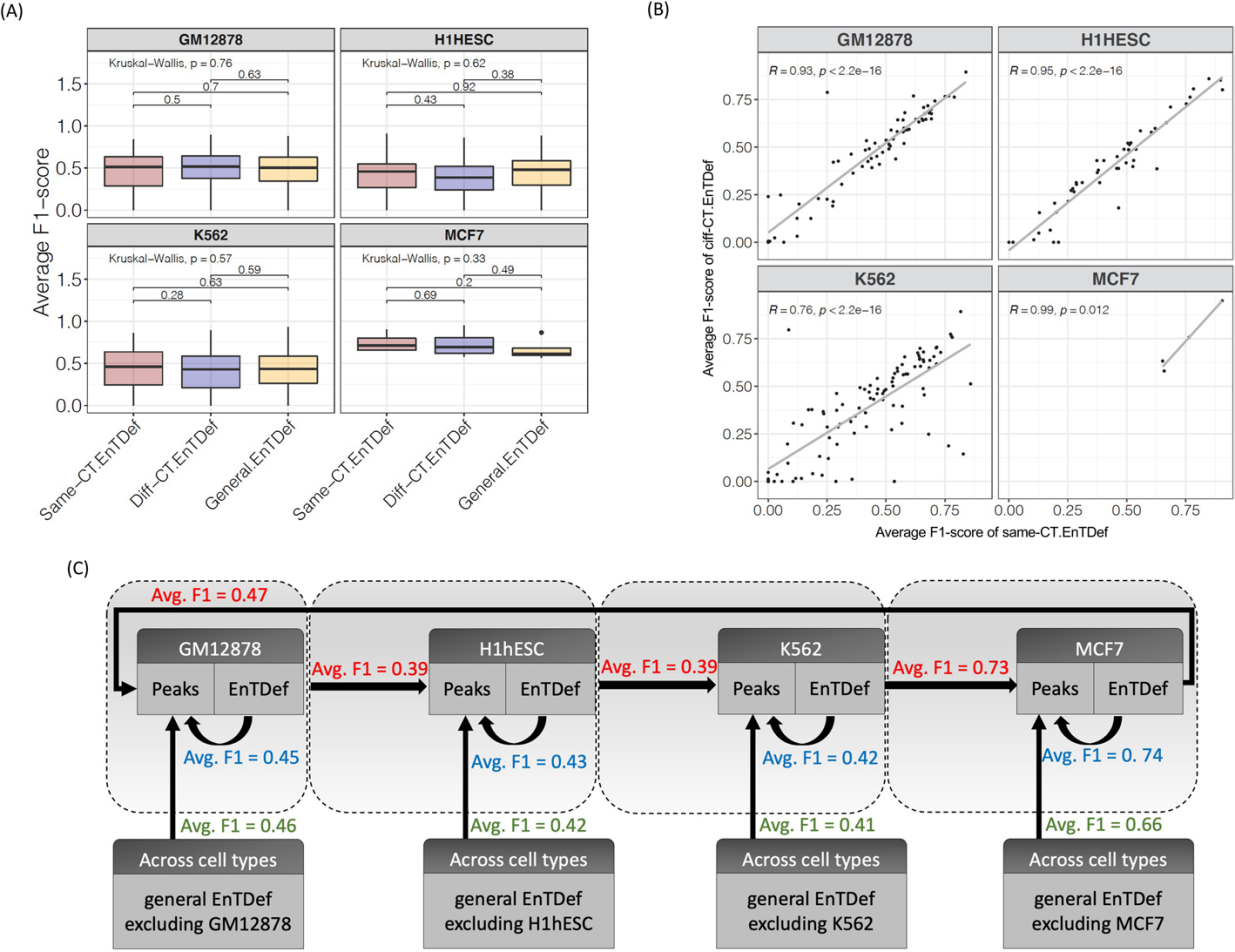
Evaluation of cell-type-specific (CT)-EnTDefs and general EnTDefs. **A** Distribution of the average F1 scores of same-CT EnTDefs, diff-CT EnTDefs, and general EnTDefs that were applied on the same TF ChIP-seq data. **B** Correlation between average F1 scores calculated on a TF in a particular cell type using CT-EnTDefs of the matched cell type (“same-CT.EnTDef” on the *x*-axis) and the ones calculated on the same TF using CT-EnTDefs of a different cell type (“diff-CT.EnTDef” on the *y*-axis). Each dot represents an average F1 score of a TF across the top 10 EnTDefs, and each panel is one of four cell types (GM12878, H1HESC, K562, and MCF7) for which the CT-EnTDefs were created and evaluated, respectively. **C** Evaluation summary of different types of EnTDefs (i.e., same-CT EnTDefs, diff-CT EnTDefs, and general EnTDefs) in four different cell types. Comparative average F1 scores associated with the TF ChIP-seq datasets of a particular cell type are grouped in a grey dashed box: blue refers to using the CT-EnTDef of the same cell type (same-CT.EnTDef), green refers to using the general CT-EnTDef excluding the enhancer-gene pairs from that cell type, and red refers to using the CT-EnTDef of a different cell type (diff-CT.EnTDef)

To contrast with the EnTDefs generated by integrating data for many cell types, hereafter called “general EnTDefs,” we created 420 “cell-type-specific EnTDefs” (CT-EnTDef) for each of the four cell types (GM12878, H1hESC, K562, and MCF7) using ChIA-PET datasets of a particular cell type, and ranked the CT-EnTDefs by average F1 scores of the evaluation ChIP-seq datasets from the same cell type (see “14”). Since many enhancers and regulatory relationships between enhancer and target genes are considered to be tissue and cell-type-specific, we sought to examine how the general EnTDefs perform when compared with CT-EnTDefs. For each tested TF (the average number of TFs tested in each cell type is ~55, ranging from 4 to 96, see Additional file 2: Table S3), the average F1 scores were calculated across the top 10 CT-EnTDefs of each cell type, or across the corresponding general EnTDefs with the same combinations of enhancer definition and enhancer-gene link methods. To prevent bias due to the incorporated cell-type-specific enhancer-gene pairs in the general EnTDefs, the ChIA-PET datasets of the particular cell type were excluded from the comparative general EnTDefs (see “14,” Fig. 3C). Three types of comparisons were performed for each TF of a particular cell type: (i) general EnTDef vs. CT-EnTDef using the same cell type (same-CT-EnTDefs), (ii) general EnTDef vs. CT-EnTDef using a different cell type (diff-CT-EnTDefs), and (iii) same CT-EnTDefs vs. different CT-EnTDefs. Notably, there was no significant difference in the average F1 scores among the three comparative EnTDefs (i.e., same CT-EnTDefs, different CT-EnTDefs, and general EnTDefs) for any cell type (Fig. 3A, Wilcoxon sum-rank test, *p* ≥ 0.2; three groups: Kruskal-Wallis test, *p* ≥ 0.3). We also observed that the average F1 scores were significantly correlated between the same-CT-EnTDef and diff-CT-EnTDef for all four cell types with Pearson’s correlation (in GM12878, H1hESC and MCF7, *R* ≥ 0.9, while in K562, *R* = 0.76) (Fig. 3B and Additional file 1: Fig. S4, *p* < 0.0001), consistent with our finding above that TF or antibody used for ChIP-seq explains a high degree of variation in F1 scores. This correlation trend still held when looking across individual TFs and EnTDefs rather than averages (i.e., F1 score per TF per EnTDef, Additional file 1: Fig. S5).

As shown in Fig. 3C, regardless of the type of EnTDef (general EnTDefs, same-CT-EnTDefs, and diff-CT-EnTDefs) used for evaluation, the average F1 score across TFs and EnTDefs were similar, with the difference ranging from 0 to 0.13. Taken together, these findings suggest that CT-EnTDefs are overall comparable to general EnTDefs, and the benefit of using CT-EnTDefs is minor and depends on the quality and quantity of data for a particular cell type (e.g., K652 vs. others, Fig. 3B). This is good news since it is costly and difficult to generate cell-type-specific ChIA-PET experiments, which are required to create the corresponding CT-EnTDef. In contrast, the general EnTDefs, which capture real enhancer and target gene interactions in a similar way to CT-EnTDefs, are more practically and economically favorable for GSE testing.

### Independent validation of our EnTDef ranking approach

**Fig. 4:**
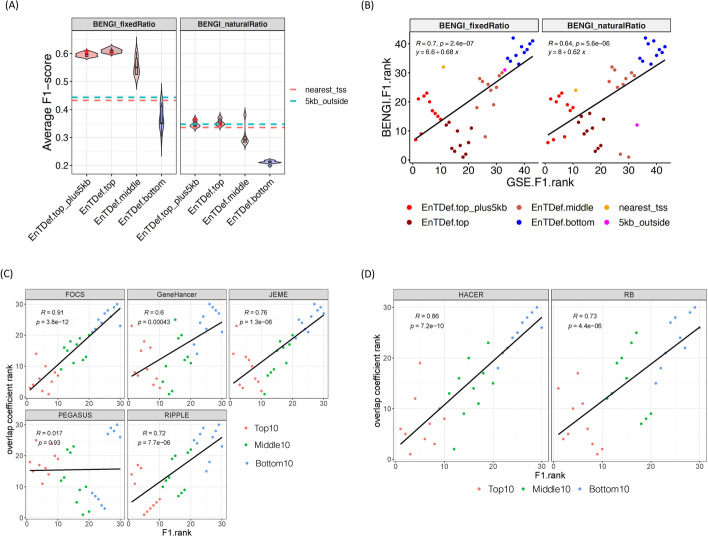
External validations of our EnTDef ranking. **A** Violin plots of average F1 scores of different types of EnTDefs (top 10 EnTDefs with 5 kb locus definition [EnTDef.top_plus5kb], top10 EnTDefs, middle 10 EnTDefs [ranked at 732–741] and bottom 10 EnTDefs) in BENGI datasets with “ambiguous pairs” removed (“BENGI_fixedRatio”: the positive and negative pairs in 1:4 ratio, “BENGI_naturalRatio”: originally generated negative pairs). The values of the GSE F1 ranked top1 EnTDefs with or without 5 kb locus definition are annotated by a red star, and the values of baseline locus definitions (nearest TSS [nearest_tss] and >5 kb [5kb_outside]) were annotated by the dashed lines (red: nearest TSS, blue: >5 kb). ANOVA test was performed between top10 EnTDefs and nearest TSS in “BENGI_fixedRatio”: *p*-value = 2.74 × 10^−104^, and in “BENGI_naturalRatio”: *p*-value = 5.48 × 10^−3^. **B** The ranking comparison of the top 10 with 5 kb (red), top 10 (dark red), middle 10 (ranked at 732–741) (coral), bottom 10 (blue) EnTDefs, nearest TSS (orange) and >5 kb (magenta) based on GSE testing-derived F1 score (*x*-axis) vs. BENGI benchmarking-derived F1 score (*y*-axis). Pearson’s correlation (R), regression equation, and the *p*-values are shown for each type of BENGI benchmark datasets. **C** The ranking comparison of the top 10 (red), middle 10 (ranked at 732–741) (green), and bottom 10 (blue) EnTDefs based on average F1 score vs overlap coefficients as compared to five computationally derived enhancer-gene pair datasets (FOCS, GeneHancer, JEME, PEGASUS, and RIPPLE) **D** and two experimental datasets (HACEER, RB)

We sought to further evaluate our EnTDef ranking using a curated benchmark of enhancer-gene interactions (BENGI) which include both true positive and true negative pair [49]. By overlapping the enhancer-gene pairs of our top 10, middle 10 (ranked at 732–741), and bottom 10 EnTDefs, as well as top10 EnTDefs with 5 kb locus definition (“EnTDef.top_plus5kb”) and the baseline methods (nearest TSS and >5 kb locus definitions), with BENGI, we calculated the F1 score, sensitivity, specificity, and precision (see “14”). The “nearest TSS” method is directly comparable to (“EnTDef.top_plus5kb”), while the “5kb_outside” method is directly comparable to (“EnTDef.top”). Consistent with our ranking approach, the top 10 EnTDefs showed the highest average F1 scores, with the values decreasing for the middle and bottom 10 EnTDefs sequentially (Fig. 4A). The average F1 scores of the top10 EnTDef were significantly higher than “nearest TSS” (EnTDef vs nearest_TSS ANOVA test in BENGI with fixed positive/negative ratio [1:4]: *p*-value = 2.74 × 10^−104^, in BENG with natural positive/negative ratio [much more negative than positive pairs]: *p*-value = 5.48 × 10^−3^), although the extent of the increase became smaller in the BENGI natural ratio dataset. The ranks of our top/middle/bottom EnTDefs and baseline methods based on the BENGI-derived F1 scores vs. those based on our original GSE testing-derived F1 scores were highly correlated (Fig. 4B), indicating general concordance between the two main benchmarks used. However, a difference is that BENGI consistently ranked methods without the 5-kb promoter regions (EnTDef.top and 5kb_outside) higher than the ones with those regions (EnTDef.top_plus5kb and nearest_tss), whereas the GSE benchmark did the opposite.

To examine the influences on the F1 scores, we assessed sensitivity and specificity separately. The overall sensitivity of the top1 EnTDef showed a 91% increase compared to that of the “nearest TSS” method (0.61 vs 0.32), while the specificity and precision decreased by ~20 and ~14%, respectively. Notably the average sensitivity of the top10 EnTDefs and EnTDefs_plus5kb were significantly increased as compared to “nearest TSS” and “>5 kb” in all BENGI subsets, while the specificity showed a small decrease (Additional file 1: Fig. S6A). The same trend can be observed in individual EnTDefs plotted as sensitivity versus (1-specificity) (Additional file 1. Fig. S6B).

Next, we directly compared the 30 EnTDefs with independent enhancer-gene pair datasets, including 5 computationally derived datasets (FOCS [50], GeneHancer [51], JEME [52], PEGASUS [53, 54], and RIPPLE [55]) and 2 experiment-based datasets (HACER [56] and the dataset from Jung et al. (referred to as RB) [57]) (Additional file 2: Table S4). The overlap coefficient (i.e., the number of enhancer-gene pairs shared between two datasets divided by the number of pairs in the smaller dataset [49]) was used to rank the EnTDefs (see “14”). The overlap coefficient-based ranks of the 30 EnTDeFs were significantly correlated with their original F1 score-based ranks in four out of the five computationally derived datasets, and both of the two experimental datasets (Pearson’s correlation ranges from 0.6 to 0.9, *p* < 0.0001, Fig. 4C, D). Moreover, we evaluated the GSE performance of the same set of top, middle, and bottom ranked EnTDefs for two dataset pairs: genome-wide DNA methylation (WGBS) and RNA-seq data for the same tumor samples comparing two subtypes of HPV-associated head and neck cancer, and ATAC-seq and RNA-seq datasets studying overexpression of the transcription factor Sox17 in the same cells (see “14”). In both cases, the top ranked EnTDefs with the regulome data were better able to recapitulate the biological processes changed in the gene expression data performed for the same experiment than the middle or bottom ranked EnTDefs (see “14” and Additional file 1: Fig. S7). These findings validate that the GSE-derived F1 score-based ranking captures true biological signal and is a valid approach to prioritize the EnTDefs.

### Comparison of the top EnTDef with other enhancer-gene pair datasets using GSE

**Fig. 5:**
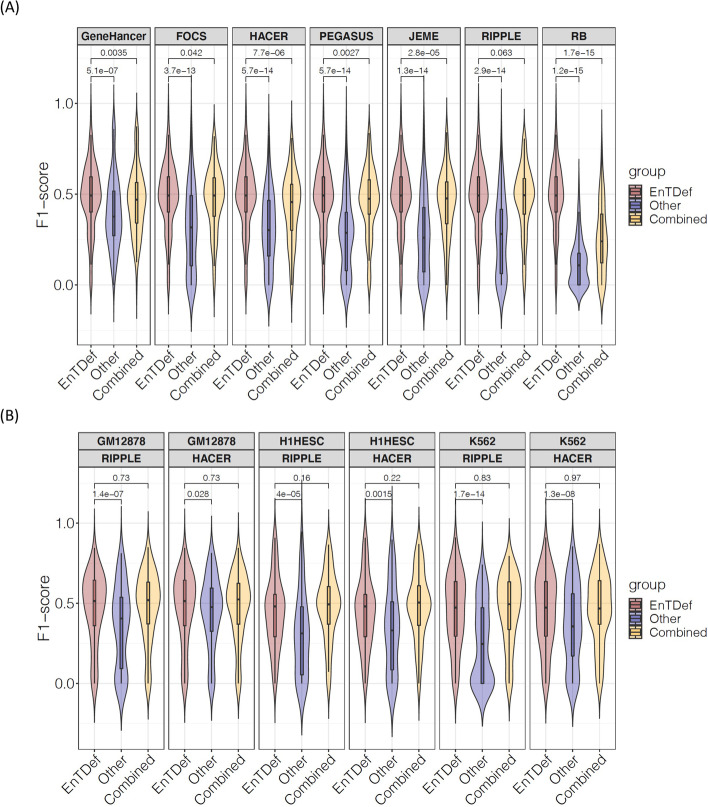
GSE performance comparison between the top ranked EnTDefs and other datasets. **A** Violin plot of F1 scores of the top EnTDef, five computational-derived enhancer-gene pair datasets (FOCS, GeneHancer, JEME, PEGASU, and RIPPLE), two datasets (HACER and RB), and the combined EnTDef with each of the seven datasets (best EnTDef + dataset). Wilcoxon signed-rank test *p*-values were shown between the top EnTDef vs the comparative dataset, or the combined dataset (best EnTDef + dataset). **B** Violin plot of F1 scores of the best CT-EnTDef, cell-type-specific RIPPLE (CT-RIPPLE), CT-HACER, and the combined dataset (best CT-EnTDef + CT-RIPPLE, or best CT-EnTDef + CT-HACER) in cell type GM12878, H1hESC, and K562. Wilcoxon signed-rank test *p*-values are shown for the best CT-EnTDef vs the comparative dataset (CT-RIPPLE, or CT-HACER), or the combined dataset (best CT-EnTDef + CT-RIPPLE, or best CT-EnTDef + CT-HACER) of the same cell type

Next, we compared the GSE performance between the best EnTDef and the aforementioned seven independent enhancer-gene pair datasets. Using the same ChIP-seq GSE evaluation method, we calculated the F1 scores of the 87 ChIP-seq datasets for each of the comparative enhancer-gene pair datasets and the combined datasets (best EnTDef + comparative dataset) (see “14”). The best EnTDef significantly outperformed the independent datasets and the combined ones (Wilcoxon signed-rank test, *p* < 0.05, Fig. 5A). Remarkably, integrating the EnTDef into the comparative datasets improved their performance, but in every case failed to outperform our top EnTDef itself. Similarly, the best CT-EnTDef performed significantly better than the two cell-type-specific datasets (CT-RIPPLE and CT-HACER) in all three investigated cell types (GM12878, H1hESC and K562) (Wilcoxon signed-rank test, *p* < 0.05, Fig. 5B). Interestingly, the performance of the combined cell-type-specific datasets (best CT-EnTDef + CT-RIPPLE, or best CT-EnTDef + CT-HACER) were comparable to that of the best CT-EnTDef. This suggests that the best EnTDef leverages sufficiently comprehensive enhancer-gene interactions based on the state-of-the-art knowledge in this field, and that coupled GSE is able to capture the biological regulatory programs from regulome data.

### Incorrect gene assignments by nearest distance method are not random

**Fig. 6:**
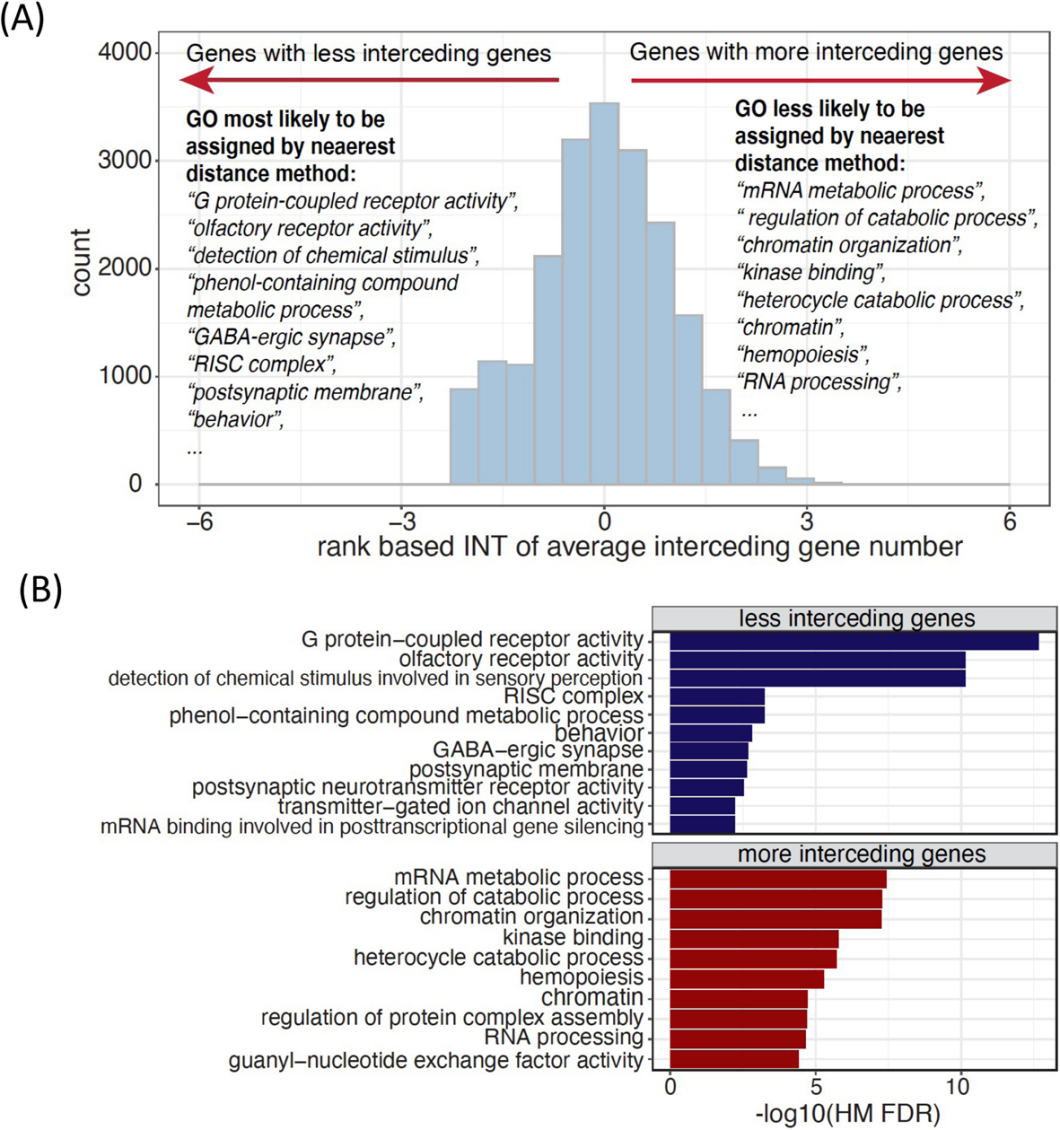
GO terms often missed or falsely identified by the nearest distance method of assigning genomic regions to target genes. **A** Distribution of the rank-based inverse normal transformation (INT) of average interceding gene numbers for the best EnTDef without the “nearest_all” addition. The top ranked enriched GO terms most likely or less likely to be identified by nearest distance method were listed. **B** The enriched GO terms in the genes with fewest interceding genes and the ones with the most interceding genes across the top 10 EnTDefs and their associated −log10 Harmonic Mean (HM) FDR

Since enhancers are known to be located up to 1 Mbp away from their regulatory genes [14, 58], several interceding genes can reside between a TF binding site (peak) in an enhancer and its target gene(s), as modeled by our EnTDefs (Additional file 1: Fig. S8). In contrast, the nearest distance method simply links a peak to the gene with the nearest TSS without accounting for interceding genes. By ranking the genes based on the average number of interceding genes across the enhancers that target them, we investigated whether the number of interceding genes is randomly distributed across genes and GO terms, or if there are GO terms significantly enriched with genes having more or fewer interceding genes [59]. We investigated the best-performing EnTDef excluding the “nearest_all” addition, in order to assess the “smart” enhancer-target links only. The genes least likely to have interceding genes were found to be significantly enriched in *G protein-coupled receptor activity* (FDR = 1.41 × 10^−14^), *olfactory receptor activity* (FDR = 6.21 × 10^−12^), *detection of chemical stimulus* (FDR = 3.23 × 10^−11^)*, phenol-containing compound metabolic process* (FDR = 1.91 × 10^−4^)*, GABA-ergic synapse* (FDR = 2.35 × 10^−4^), *RISC complex* (FDR = 2.39 × 10^−4^), *postsynaptic membrane* (FDR = 4.13 × 10^−4^), and *behavior* (FDR = 4.71 × 10^−4^) (Fig. 6A). These GO terms enriched with genes least likely to have interceding genes (lower-ranked genes) are most likely to be correctly assigned by the nearest distance method (Additional file 1: Fig. S1: >5 kb LocDef), and thus most easily detectable by current GSE testing. Conversely, the GO terms enriched with higher numbers of interceding genes (upper ranked genes) were *mRNA metabolic process* (FDR = 8.09 × 10^−8^), *regulation of catabolic process* (FDR = 8.40 × 10^−8^), *chromatin organization* (FDR = 2.53 × 10^−7^), *kinase binding* (FDR = 1.75 × 10^−6^), *heterocycle catabolic process* (FDR = 3.22 × 10^−6^), *chromatin* (FDR = 7.25 × 10^−6^), *hemopoiesis* (FDR = 9.47 × 10^−6^), and *RNA processing* (FDR = 2.27 × 10^−5^) (Fig. 6A). Those GO terms are least likely to be assigned by the nearest distance method, and most likely missed using current methods for GSE testing.

To determine if this observation is robust to different EnTDefs, we performed the same analysis on all top 10 best-performing EnTDefs without the “nearest_all” addition, and combined the results by calculating FDR-adjusted harmonic mean *p*-values, followed by removing redundant terms (see “14”). Consistently, *G protein-coupled receptor activity*, *olfactory receptor activity*, *RISC complex*, and *postsynaptic membrane* were still the top 5 enriched terms for the genes with fewer interceding genes, and similarly, *regulation of catabolic process*, *chromatin organization*, *kinase binding*, and *heterocycle catabolic process* were the top 5 enriched terms in upper ranked genes with more interceding genes (Fig. 6B). These findings indicate that both the genes with the most and fewest interceding genes are not random: chemical stimulus and neuron-related genes can be easily assigned with the nearest distance method, whereas metabolic processing and chromatin organization genes may be frequently missed. It is concordant with the knowledge that enhancers regulate genes via long-range chromatin interactions, which are able to be captured by our EnTDefs.

### Guidance for selecting a peak-to-gene assignment method in GSE analysis

**Fig. 7:**
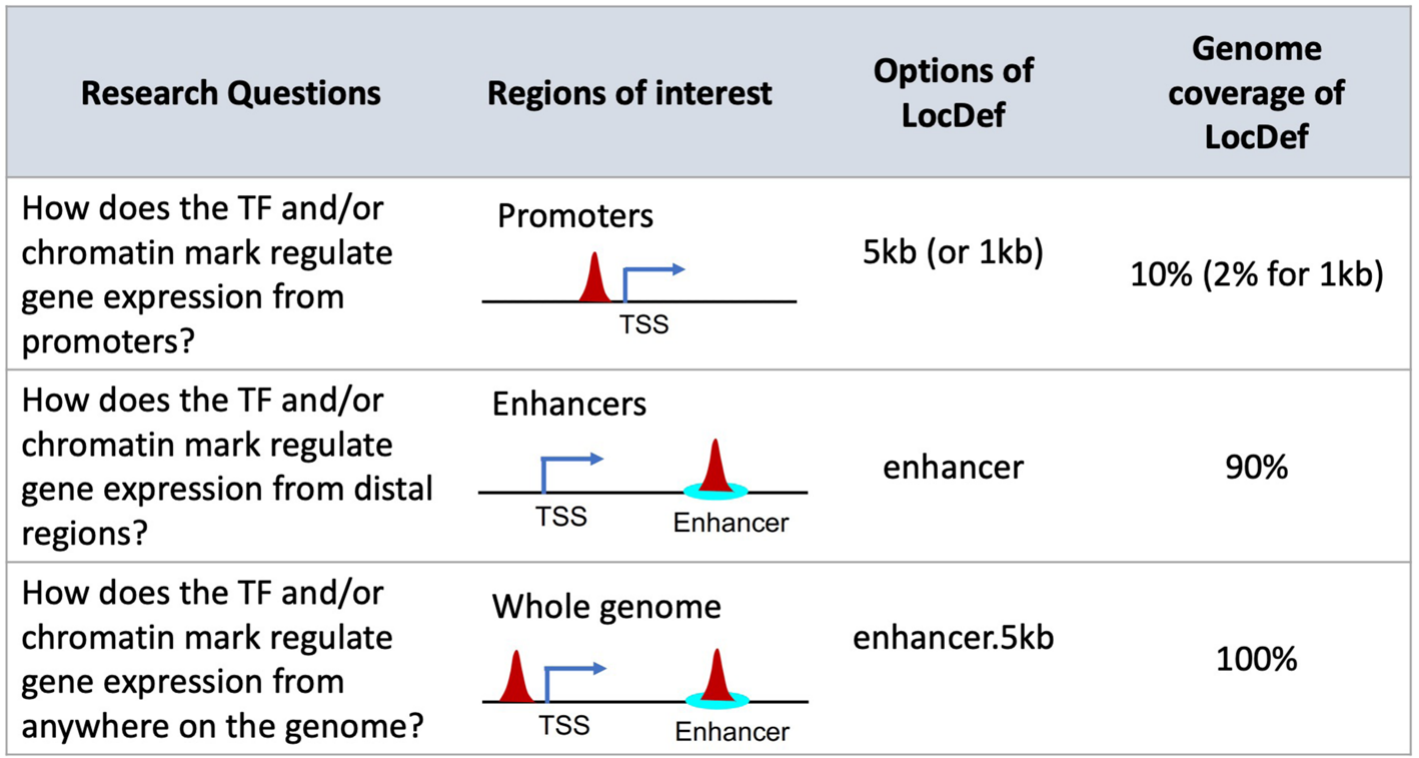
User guidelines for selecting an appropriate enhancer-to-gene assignment method (LocDef) for GSE testing. Depending on the specific research questions, three types of LocDefs can be selected for GSE testing from the *chipenrich* R package: (1) “5 kb” or “1 kb” for promoter regulation, (2) “enhancer” for distal regulation, and (3) “enhancer.5kb” for whole genome regulation. Different LocDefs have different genome coverages as shown in the last column. Options in other GSE testing software for genomic regions will differ. We no longer recommend using nearest TSS method for Poly-Enrich analysis

The first step in GSE testing of cis-regulome data, such as TF binding sites or chromatin marks from ChIP-seq, is to assign the genomic regions or peaks to their target genes. The different assignment methods can lead to variable enrichment results and FP and/or FN findings, as discussed above (nearest distance method vs. EnTDef). To avoid misinterpretation of genome-wide regulatory data, we need to select an appropriate LocDef method with care, which should be specific to the particular research question and the genomic regions of interest. Figure 7 summarizes three general categories of research questions and the corresponding regions of interest: (i) the 5 kb or 1 kb LocDef should be selected when interested in how a TF and/or chromatin mark regulates gene expression from promoters; (ii) the EnTDef (enhancer) should be selected when interested in how a TF and/or chromatin mark regulates gene expression from distal regions; and (iii) when the comprehensive regulatory signature is of interest, including both promoter and distal regions, our EnTDef plus 5kb LocDef (enhancer.5kb) should be selected. The promoter LocDef has the lowest genome coverage (10% for <5 kb LocDef and 2% for <1 kb LocDef), while the EnTDef plus 5 kb has 100% genome coverage, and the EnTDef has intermediate genome coverage (90%). We incorporated our top-performing EnTDef and EnTDef.plus5kb into the Bioconductor package *chipenrich* [42] and the ChIP-Enrich website (chip-enrich.med.umich.edu), allowing users to select the most suitable genomic region-gene assignment methods, gene sets, and GSE method to correctly interpret their genome-wide regulatory data. In addition, we provide a peak-to-gene assignment functionality in our GSE Suite (gsesuite.dcmb.med.umich.edu), by which users can select any possible combination of enhancer location and enhancer-to-gene target methods (as described in this study) and obtain the gene assignments for a user uploaded list of genomic regions, based on the selected EnTDef, or other method (e.g., promoters, exons, introns or anywhere in the genome).

## Discussion

A greater appreciation of the central role that distal regulatory elements play in genetic diseases and cancers has motivated a multitude of enhancer studies. As a result of the increasing availability of functional genomics data, growing attention has been paid to matching Enhancer-Target Gene pairs (ETG) in the field of computational biology and genomics. Over the past decade, a variety of algorithms and tools have been developed by leveraging multiple genomic features and functional data, as recently reviewed in [60]. Briefly, they can be categorized into four groups: (1) correlation-based (e.g., Thurman et al. [38], PreSTIGE [61], ELMER [62, 63]); (2) supervised learning-based (e.g., IM-PET [64], TargetFinder [65], McEnhancer [66]); (3) regression-based (e.g., RIPPLE [55], JEME [52], FOCS [50]); and (4) score-based methods (e.g., EpiTensor [67], GeneHancer [51], and PEGASUS [53, 54]). Although these algorithms have significantly advanced our knowledge of ETGs, they are affected by one or more of the following issues: (1) the lack of a genome-wide exhaustive reference list of enhancers; (2) the lack of a large gold standard which is required for supervised learning algorithms, i.e., experimentally validated true positive and true negative enhancer-target gene pairs, and (3) the lack of a systematic evaluation of their reliability and generalization in various cell types. To overcome these issues, we developed a gold standard-free approach to generate and prioritize comprehensive sets of ETGs based on their performance in the interpretation of regulome data. We further validated our results by comparing to an experimentally derived gold standard ETG set [49], and compared our top ranked enhancer-target gene pair dataset to others. The benchmarking analysis based on the BENGI dataset revealed a remarkable increase in recall (sensitivity) and a moderate decrease in specificity and precision of our top EnTDef when compared with the “nearest TSS” approach. Thus, our top EnTDef approach is likely not optimal for those using an enhancer-target gene links database where it is more important to limit false positive links than to identify true ones (i.e., specificity is much more important than sensitivity). However, for gene set enrichment, identifying likely enhancer-target links that will be validated by experiment, or other situations where it is important to have high sensitivity, our top EnTDef has been well optimized and clearly outperforms the nearest TSS approach.

In this study, we identified a best set of enhancer-to-target gene definitions (EnTDefs) by investigating and evaluating all possible combinations of existing reliable sources for human enhancer location definitions and enhancer-target gene pair definitions across various cell types. Purposely, we coupled EnTDefs with GSE testing to systematically evaluate their performance when interpreting regulome data. By carefully selecting datasets of high quality and resolution, we explored ENCODE ChromHMM, DNase-seq, FANTOM5, and Thurman datasets for enhancer regions and ENCODE ChIA-PET interactions, Thurman DHS correlation-based, FANTOM5, and ENCODE ChIA-PET CTCF loop-based enhancer-target gene interactions. We also systematically evaluated the performance of all possible combinations of datasets when applied on ENCODE TF ChIP-seq data in GO GSE testing and compared the enriched GO terms with the curated TF GO annotations (TF-annotated GO BP terms by the GO database). In contrast to the statistical model-based or machine learning-based algorithms as described above, our approach integrates various data sources and directly couples the EnTDefs with GSE testing for a systematic evaluation, resulting in an EnTDef with maximally balanced sensitivity and specificity (assessed by F1 score). Our approach to generating EnTDefs is assumption-free and independent of true positive/negative pairs, but based on a systematic evaluation using GSE testing. The results demonstrate that the DNase-seq and FANTOM5 enhancers with the integrated enhancer-target gene pairs from ChIA-PET, Thurman, and FANTOM5 interactions performed best, suggesting that both chromosome accessibility and conformation, as well as transcriptional correlation, are beneficial for identifying enhancer-target regulatory relationships.

The nearest distance method, which naively assigns genomic regions of interest to the nearest gene, is commonly used in GSE for regulome data. Our analysis showed that this naïve approach will commonly fail to identify genes in certain functions that tend to have one or more interceding genes, such as mRNA metabolic process and chromatin organization. This is concordant with our results showing that our top EnTDef has nearly twice the sensitivity as the nearest TSS approach using the BENGI benchmark. However, in spite of its shortfall, linking genomic regions outside known enhancers to the nearest gene significantly improved GSE results, illustrating that the field still has far to go in defining all human enhancer locations and their gene targets, and the importance of comprehensive coverage in GSE testing. By comparing with BENGI, an experimentally derived gold standard ETG set, and seven independent computational and experimentally derived ETS sets, we validated our EnTDef prioritization approach and showed that our best general and cell-type-specific EnTDefs significantly outperformed the alternative datasets.

Our EnTDefs were generated by leveraging different genomic data across >500 cell types and can be applied to different cell types, demonstrating performance comparable to their cell-type-specific counterparts. Our top integrated EnTDef based on many cell types represents a comprehensive set of enhancer regions (only a subset of which will be active in any one cell type); our data indicate this performs well because current cell-type-specific enhancer-target genes (ETGs) are not yet sufficiently comprehensive (except for a few cell types such as GM12878). Research performed on cancer samples, less commonly used cell lines, and other complex tissue samples will greatly benefit from this integrated EnTDef. While cell-type-specific ETGs are important for studying regulation at specific locations, our results demonstrate that for genome-wide approaches such as GSE, the comprehensiveness outweighs the need for specificity.

Besides DNase-seq, ChIA-PET, CAGE-seq, and RNA-seq data, Hi-C and eQTL data are also used to infer ETG [51, 68]. However, we found that current Hi-C data often have insufficient resolution, with genomic windows being a few to several kilobases wide due to low coverage, and high-quality Hi-C data is not available for nearly as many cell types as the other approaches. Although eQTL data is available for many tissues and cell types, it is similarly restricted by limited population diversity and low resolution. The tissue-specific eQTL data from the GTEx project [69] is widely used; however, it was generated for only 49 tissues from <1000 donors with the majority being Caucasian (84.6%), making it difficult to apply to other tissues/populations. In addition, eQTL data is highly correlated with the linkage disequilibrium, and thus its resolution is associated with the size of haplotype blocks, which is highly variable across populations (on average ~10 kb) [70], whereas enhancers are usually short genomic regions (50–1500 bp). Due to this low resolution of Hi-C and eQTL data, we excluded them from our analysis. However, given the rapid scientific and technological advances, exponential accumulation of more accurate and comprehensive ETG datasets can be expected. In future work, we will update the EnTDef by incorporating additional high-quality datasets and further boost our EnTDef performance when coupled with GSE.

In conclusion, we identified a best set of enhancer-target gene pairs (EnTDef) by leveraging existing data sources of chromosome accessibility and/or conformation and transcriptome data across numerous cell types, which significantly improved the biological interpretation of distal regulation in GSE compared to assigning genomic regions to the nearest gene. Our approach performs well across a wide range of cell types, making it feasible to apply on extensive genomic data sets. The limitations of our EnTDef are inherited from the existing data sources, including low genome coverage, low resolution, and small number of cell types with good-quality ChIA-PET data. With the continued growth in volume of functional genomics data and advances in data quality and resolution, we expect further improvement of our EnTDef in the future.

## Conclusions

In summary, we provide an optimized enhancer-to-target gene assignment approach, which is critical for interpreting genome-wide regulatory data. This study has important implications for which type of enhancer-target gene methods are most accurate, and the relative importance of comprehensiveness versus cell-type-specific accuracy. To the best of our knowledge, there is currently no such comprehensive resource of distal regulatory region-to-target gene links which are feasible to apply on various types of regulome data (e.g., ChIP-seq, ATAC-seq, WGBS) regardless of cell types.

## Methods

### Generation of general enhancer-target gene definitions

We generated genome-wide definitions of human distal enhancer locations and their target gene assignments for the hg19 genome using all possible combinations of the below enhancer location methods and enhancer-gene linking data (Fig. 1A: Enhancer, Extension, Enhancer-target gene link, and Additional links). These are based on enhancers from: (1) “ChromHMM”: ENCODE ChromHMM UCSC tracks (9 cell types) [43], (2) “DNase-seq”: DNase hypersensitive sites (DHSs) from 125 cell types processed by ENCODE [38], (3) “FANTOM5”: Cap Analysis Gene Expression (CAGE) experiment-derived enhancers across 421 distinct cell lines/tissue/primary cells from FANTOM5 project [37, 44, 45], and/or (4) “Thurman”: distal and non-promoter DHS within 500 kb of the correlated promoter DHSs from 79 cell types, referred to as the first author of the publication [38]. Since our motivation was to identify the target genes of distal regulatory elements that do not have clear target genes based on close proximity to a TSS, we constrained the enhancer regions to be outside of 5 kb from a transcription factor start site (TSS) by trimming the bases from the above defined enhancers overlapping with the 5-kb windows of TSSs. The hg19 TSS locations were obtained from the Bioconductor *chipenrich* package version 3.5.0 [42]. To identify target genes, we used: (1) “ChIA” method: enhancer and gene interactions identified by ChIA-PET2 using default parameters [71] from 10 ChIA-PET datasets of 5 cell types (Additional file 2: Table S5) [46, 47], (2) “Thurman” method: the enhancer and promoter interactions identified by Thurman et al., which were defined by high correlation (*r* > 0.7) between cross-cell-type DNase I signal at each DHS position and all promoters within ±500 kb [38], (3) “FANTOM5” method: the regulatory targets of enhancers predicted by correlation tests using the expression profiles of all enhancer-promoter pairs within 500 kb [45], and (4) “Loop” method: any possible interactions between enhancers and genes that are encompassed within in a RAD21, cohesin, and/or CTCF ChIA-PET loop with convergent CTCF motifs [48], and depending on the number of genes included in the loop, this method was referred to as “L1” (one gene), “L2” (≤ two gene), or “L3” (≤ three genes) (Fig. 1B).

All possible combinations of the enhancer definition datasets (*n* = 4) and enhancer-gene pair datasets (*n* = 4), allowing multiple at a time, defined 465 of the enhancer-to-target gene definitions (EnTDefs) (Fig. 1A, B), i.e., the combinations of 15 enhancer definitions (4C4 + 4C3 + 4C2 + 4C1 = 15; C denotes “choose”) and the 31 enhancer-gene pair methods, which include the combinations without “L” method (3C3 + 3C2 + 3C1 = 7), the combinations with L1, L2, or L3 (7 × 3 = 21), and the L1/L2/L3 method only (*n* = 3), resulting in the 15 × 31 = 465 core EnTDefs. In addition, to increase the genome coverage, we tested extending the enhancer regions to 1 kb (i.e., “enhancer extension,” 500 bp extension at both sides of the midpoint) (resulting in 465 ×2 = 930), and assigning regions outside of enhancers and promoters (within 5 kb of a TSS) to the gene with the nearest TSS (i.e., “nearest_all” addition) (for a total of 930 ×2 = 1860 distinct EnTDefs).

### Evaluation of enhancer-target gene definitions

To evaluate the performance of each individual EnTDef, we performed Gene Ontology (GO Biological Processes [GOBP]) enrichment testing using Poly-Enrich [72] in the *chipenrich* Bioconductor package [42] on 87 ChIP-seq datasets of 34 TFs selected from the tier 1 ENCODE cell lines (Additional file 2: Table S6). To minimize runtime for the initial pass analysis, we used the PE. Approx method (an approximate version of Poly-Enrich [72], see “14” below and Additional file 1: Fig. S9). We then compared the significantly enriched GOBP terms with the GO BP annotations of each TF (i.e., the GOBP terms assigned to the 34 TFs by the GO database, excluding the terms with <15 or >2000 assigned genes) (Fig. 1C: Evaluation of the Enhancer-Target gene Definition), to identify the EnTDefs with greatest concordance. The assumption of this approach, used previously in [72, 73], is that TFs tend to the regulate genes in the biological processes to which they belong, and thus greater overlap with TF GO BP annotation indicates more accurate enrichment results, and thus more accurate peak-to-gene assignments. We used the Gene Ontology Biological Process (GO BP) terms that each of the 34 TFs were assigned to by the GO database [74, 75], and extracted the GOBP-to-gene assignments from the human annotation Bioconductor package org.Hs.eg.db [76]. We reasoned that TFs tend to regulate genes in the biological processes to which they are assigned in GO [77–80]. Since the GO BP terms describe “the larger processes, or ‘biological programs’ accomplished by multiple molecular activities” [75] and the function of a TF is to regulate genes that coordinate a common biological process, it is logical to assume that TFs tend to regulate genes in the GO BP to which they belong. Although a TF may not regulate all of their assigned GO terms in every cell type, we assume that higher concordance with this set (in terms of sensitivity and specificity) corresponds to superior results. To alleviate the bias caused by the unbalanced positive and negative assignments (i.e., each TF only regulates a very small percent of the total GO terms), we generated the same number of true negative assignments for each TF as there were positive by randomly selecting GOBP terms from the set that were not assigned to the particular TF, and excluding the offspring terms and their siblings of the assigned terms (hereafter called “true negative” terms, depicted in Additional file 1: Fig. S10A). In order to control for the confounding of GOBP size (i.e., the number of assigned genes to each GOBP term), random sampling was performed among the negative terms of comparable size to the corresponding true positive term (bin size = 20). In each sampling, the PE results were assessed by the number of true positive (TP), false positive (FP), true negative (TN), and false negative (FN) GOBP terms according to the following definitions: (1) TP: the number of GOBP terms that were significantly enriched (FDR < 0.05) and assigned to the TF by the GO database; (2) FP: the number of GOBP terms that were significantly enriched (FDR < 0.05), but not assigned to the TF by the GO database; (3) TN: the number of GOBP terms that were not significantly enriched (FDR > 0.05 or “depleted”) and also not assigned to the TF; and (4) FN: the number of GOBP terms that were not significantly enriched (FDR > 0.05, or “depleted”), but assigned to the TF. The *F1 score* ($F1\ score=2\times \frac{Precision\times Recall}{Precision+ Recall}$) was calculated to measure the overall performance of an EnTDef for a TF. We repeated the sampling process 10 times, and took the average *F1 score* for each EnTDef and TF. The average of these F1 scores across TFs provided the final ranking for each EnTDef.

To assess the robustness of our approach, we also evaluated the performance of EnTDefs using more conservative GO annotations, in which GOBP assignments based on “automatically assigned, inferred from Electronic Annotation” (IEA) were excluded, thus minimizing false annotations in GOBP. For the positive GO annotations, we used only the leaf GO terms (the lowest level in the GO hierarchical tree) and their parent and grandparent GO terms, while the negative terms were sampled from all other terms, excluding positive terms, and ancestors of positive terms, siblings of ancestors of positives, and offspring of positives (depicted in Additional file 1: Fig. S10B).

After ranking all EnTDefs in descending order by their average F1 scores (Fig. 1C: Rank of EnTDef), we identified the set of best EnTDefs. Paired Wilcoxon signed-rank tests were performed to compare the F1 score of the 1st ranked EnTDef with each of the sequential ones, and the rank at which the EnTDef showed significantly lower F1 score than the 1st ranked one (*p* < 0.01) was selected as the cutoff. The EnTDefs ranking above the cutoff were defined as the best set of EnTDefs. In addition, we performed the same F1 score evaluation of previously defined methods for genomic region-to-gene assignments, termed gene Locus Definitions (LocDefs, see Additional file 1: Fig. S1 for details) that do not use “smart” enhancer-target links (i.e., “>5kb”: distal regions assigned to the gene with the nearest TSS; “<5kb”: regions within 5 kb of a TSS assigned to the gene with that TSS; and “nearest TSS”: all regions assigned to the gene with the nearest TSS). These LocDefs are used by Poly-Enrich in the *chipenrich* R Bioconductor package [42] and represent the current standard practice for enhancer-to-gene assignments for gene set analysis. The F1 scores were compared between each EnTDef and the distal nearest distance (“>5kb”) LocDef by Wilcoxon signed-rank tests. We also evaluated and compared two commonly used Gene Set Enrichment (GSE) testing methods, Fisher’s exact test (FET) and GREAT [39], which were implemented by the R *chipenrich* package using the FET and binomial method respectively, coupled with the “5kb” LocDef. To obtain a final assessment, a second round of GSE testing using Poly-Enrich (*PE.Exact* method; see “14” for details) was applied on the subset of EnTDefs which significantly outperformed the nearest distance assignments (>5 kb LocDef), and the average F1 scores were calculated and used to refine the final ranking of EnTDefs.

#### Comparison between Poly-Enrich approximate (PE.Approx) and exact (PE.Exact) methods in GSE testing

For the first round of evaluating enhancer-to-target gene definitions (EnTDefs), we applied a faster, approximate version of Poly-Enrich (PE) test, which utilized the score test instead of likelihood ratio test. Compared to the Poly-Enirch likelihood ratio test (i.e., PE.Exact method), the Poly-Enrich score test (i.e., PE.Approx method) requires the least amount of computation time in exchange for lacking power in negative binomial families [81]. However, the score test is a good approximate test for situations when one needs a large amount of preliminary results. We used the *glm.scoretest* function from the *statmod* [82] package to compute the score test for PE.Approx method. The PE.Approx runs over 30 times faster and is reasonably concordant with the PE.Exact for enriched GO terms, but less similar for depleted GO terms (Additional file 1: Fig S9A). The *F1 score* of the top 19 best EnTDefs derived from the PE.Approx GSE method was highly correlated to that derived from the PE.Exact method (Pearson’s correlation *r* = 0.97, *p* < 0.00001, Additional file 1: Fig S9B).

### Validation of the EnTDefs with ChIP-seq data from different cell types

To further evaluate the performance of EnTDefs in different cell lines, we selected 13 additional ENCODE ChIP-seq datasets from four non-tier 1 ENCODE cell lines (A549, HEPG2, HUVEC, and NB4), which contain ChIP-seq experiments for at least three TFs in each cell line (Additional file 2: Table S7). In comparison to the 87 *evaluation* ChIP-seq peak sets from ENCODE tier 1 cell lines (GM12878, H1HESC, and K562), these 13 datasets are the *test* datasets. The six TFs (*C-JUN, C-MYC, CEBPB, CTCF*, *MAX*, and *NRSF*) assayed by the 13 *test* datasets were also included in the 87 *evaluation* datasets. The top 10 best EnTDefs were evaluated using the *PE.Exact* method as described above and these 13 test ChIP-seq datasets. For each EnTDef, the average F1 score across the 13 ChIP-seq datasets was calculated and compared with the average F1 score generated using the evaluation ChIP-seq datasets (*n* = 16) of the corresponding TFs.

### Generation of cell-type-specific EnTDefs

We used “ChIA” and/or “L”-derived enhancer-to-gene assignment methods (Fig. 1B) to generate cell-type-specific enhancer-target gene definitions, hereafter called CT-EnTdefs. Since the enhancer-gene linking data defined by Thurman and FANTOM5 datasets were non-cell-type specific, we did not include these. The cell types were selected based on the availability and quality of cell-type-specific ChIP-seq and ChIA-PET data in ENCODE. As shown in Additional file 2: Table S3, four cell types were selected: GM12878 (tier 1), H1-hESC (tier 1), K562 (tier 1), and MCF7 (tier 2). The multiple ChIA-PET datasets were combined for each cell type. All combinations of enhancer location definitions, along with ChIA, L1 (or L2 or L3) enhancer-gene assignment methods, with or without enhancer location extension and “nearest_all” addition, were used to generate the CT-EnTDefs, resulting in a total of 420 CT-EnTDefs for each of the 4 cell types.

### Evaluation of CT-EnTDefs

To evaluate the performance of the CT-EnTDefs, we performed GSE testing of Gene Ontology (GO Biological Processes [BOBP]) using Poly-Enrich [72] on the TF ChIP-seq peak sets of the same cell type from which each CT-EnTDef was generated (Additional file 2: Table S3. See details as described above), and ranked the CT-EnTDefs using average F1 scores across the evaluation ChIP-seq datasets in descending order. For comparison, we also applied GSE testing on the same TF ChIP-seq peak sets using the corresponding general EnTDefs (i.e., not cell-type specific, using the same enhancer regions and target gene link methods as those of the comparative CT-EnTDef, but excluding the enhancer-gene pair datasets from the same cell type), as well as the CT-EnTDef from a different cell type (Fig. 3C, i.e., MCF7 CT-EnTDefs were applied on GM12878 TF ChIP-seq peaks, GM12878 CT-EnTDefs on H1hESC peaks, H1hESC EnTDefs on K562 peaks, K562 EnTDefs on MCF7 peaks). For each TF, the average *F1* scores across the top 10 CT-EnTDefs (ranked by average F1 scores of all TFs) or their 10 general EnTDef counterparts were calculated and compared. For each cell type, Pearson’s correlation test was used to evaluate the pair-wise correlation among the F1 scores, and Wilcoxon rank-sum test was used to compare their differences. Finally, the overall performance of CT-EnTDefs, general EnTDefs, and different cell-type CT-EnTDefs were assessed using the average F1 scores across all evaluated EnTDefs and TFs in each cell type.

### EnTDef ranking by comparing with other enhancer-gene pair datasets

Moore et al. published a curated benchmark of enhancer-gene interactions for evaluating enhancer-target gene prediction methods in 2020 [49]. In their Benchmark of candidate Enhancer-Gene Interactions (BENGI) dataset, they integrated the recently developed Registry of candidate cis-regulatory elements (cCREs) with experimentally derived genomic interactions (positive pairs), and generated the corresponding negative pairs using the nearest distance methods. For the positive pairs, they either kept all original experimentally derived interactions, (“allPairs”) or removed the pairs with the ends within 2 kb of the TSSs of multiple genes (“removeAmbiguousPairs”). For the negative pairs, they either kept all originally generated negative pairs (“naturalRatio”) or fixed the positive and negative pairs in 1:4 ratio (“fixedRatio”). For simplicity, we show the results for “removeAmbiguousPairs” since results and figures were nearly identical; however, we show results for both natural and fixed ratio, since these made a noticeable difference. Thus, two types of benchmark datasets are shown: “fixedRatio” (the negative pairs in 1:4 ratio) and “naturalRatio” (the originally generated negative pairs). For each type of the benchmark datasets, we compared the top 10, middle 10 (ranked at 732–741 which was the point at which the EnTDefs no longer significantly outperformed the naïve nearest gene approach), and bottom 10 EnTDefs, as well as the top10 EnTDef with 5 kb locus definition (“EnTDef.top_plus5kb”) and baseline locus definitions (nearest TSS and > 5kb), with each of the cell type and experiment-specific BENGI subsets, and identified overlapped enhancer-gene pairs, defined by enhancer regions overlapping by at least 1 bp and having the same linked genes. The sensitivity, specificity, precision, and F1 score were calculated (see “14”) and compared between the top/middle/bottom EnTDefs (as well as the top EnTDefs with 5 kb locus definition [EnTDef.top_plus5kb]) and baseline methods (nearest TSS and >5 kb). The average F1 scores among all BENGI subsets was used to rank the 30 EnTDefs in descending order.

In addition, we compared the enhancer-gene pairs between the 30 EnTDefs and seven independent datasets, including 5 computationally derived datasets (FOCS [50], GeneHancer [51], JEME [52], PEGASUS [53, 54], and RIPPLE [55]) and 2 experiment-based datasets (HACER [56] and the dataset from Jung et al. (RB) [57]), and calculated overlap coefficients as used by Moor et al. [49]. To make a fair comparison, all the datasets were pre-processed by excluding the 5-kb regions around a TSS, and merging enhancer regions. The 30 EnTDefs were ranked by their overlap coefficients in descending order as compared to each of the seven datasets, and the ranks were correlated with their average F1 score-based ranks. Pearson’s correlation test was performed.

#### Benchmarking EnTDefs using BENGI dataset

Firstly, we identified the overlapped enhancer regions between our EnTDefs and each of the cell type and experiment-specific BENGI sub-datasets, and then called true positive (TP), false positive (FP), true negative (TN), and false negative (FN) for each of the BENGI pairs according to the following criteria: (i) TP call when BENGI pair was positive and the assigned gene was also assigned to the overlapped enhancers in the comparative EnTDef or baseline methods; (ii) FP call when BENGI pair was negative while the assigned gene was assigned to the overlapped enhancers in the comparative EnTDef or baseline methods; (iii) TN call when BENGI pair was negative and the assigned gene was not assigned to the overlapped enhancers in the comparative EnTDef or baseline methods; and (iv) FN call when BENGI pair was positive while the assigned gene was not assigned to the overlapped enhancers in the comparative EnTDef or baseline methods. Using the number of the BENGI pairs in each of the four categories (TP/FP/TN/FN), we calculated and reported the average true positive rate (i.e., sensitivity or recall, TF/[TF+FN]), false positive rate (i.e., 1-specificity, 1-TN/[TN+FP]), precision (positive predictive value, TP/[TP+FP]), and F1 scores (i.e., the harmonic mean of precision and sensitivity, 2TP/[2TP+FP+FN]). The average scores were taken across the BENGI cell type and experiment-specific sub-datasets for each EnTDefs and baseline locus definitions and compared between them.

### Case studies of GSE using EnTDefs for WGBS or ATAC-seq experimental data with transcriptional data in parallel

To further evaluate the performance of EnTDefs on real experimental datasets, we performed GSE testing using our top10, middle10 (ranked at 732–741) and bottom 10 EnTDefs on two types of regulome datasets: whole genome bisulfite sequencing (WGBS) [83] (GEO series GSE180260) and ATAC-seq [84], both of which had corresponding RNA-seq data generated in the same experiments.

The RNA-seq/WGBS study compared two HPV-associated head and neck squamous cell carcinoma subtypes (IMU vs KRT) from Zhang et al. [83]. The adaptors were trimmed from raw WBGS sequences by Trim Galore (v0.4.1) (https://github.com/FelixKrueger/TrimGalore) and Cutadapt (v1.10) [85]. The trimmed sequences were aligned to human genome (hg19) and the percentage of methylated reads at each CpG was calculated using Bismark (v0.16.1) [86] with the “--directional -q --score-min L,0,-0.2 --ignore-quals” options. The significant (FDR<0.05) differentially methylated regions (DMRs) were calculated using MethylSig (v0.99) [87] with 100 bp tiling window, “min.group.num >= 80%” and controlling for sex, age, stage, and smoking status. In total, 270,326 DMRs were identified between IMU and KRT subgroups. The Gene Ontology (GO) GSE testing was performed on those DMRs by *polyenrich* [72] with “weight” option using the methylation differences as the weights. All GO terms (GOBP, GOMF, and GOCC) with 10 to 1500 assigned genes were tested.

The RNA-seq/ATAC-seq data [84] was designed to study the overexpression of the transcription factor SOX17. The original ATAC-seq fastq files were downloaded from GEO (GSE140341), Fastqc (0.11.9) [88] was used to check the quality, cutadapt (3.4) [85] to trim the adapters, and then Bowtie2 (2.4.4) [89] to align the reads to hg19 reference genome. Differential peak calling was performed by *Genrich* with “ATAC-seq” option [90]. *Chipenrich* (2.1.0) was used for GO GSE testing with the 5651 differential ATAC-seq peaks (*p*-value < 0.05). For both experiments, the RNA-seq data were processed using edgeR (3.34.0) for differential gene expression analysis and RNA-Enrich [91] for GO GSE testing.

For both studies, the GO terms (GOBP, GOMF, and GOCC) significantly enriched for differential genes were defined as the benchmark for interpretation of biological changes between the comparative groups. The top 100 significant GO terms (*q*-value < 0.05) and 100 randomly selected insignificant GO terms (*p*-value > 0.5) with comparable gene set size (i.e., the number of signed genes) from RNA-seq GSE results were used as the true positives (TPs) and true negatives (TNs), respectively. The significant GO terms identified by WGBS or ATAC-seq GSE testing were compared with those TPs and TNs, and the true positive/false positive rates were calculated at different FDR cutoffs. For each tested EnTDefs, we repeated the random sampling of the TNs for 100 times, and reported the average AUC values (area under the receiver operating characteristic curves), with a larger average AUC value representing a more accurate recapitulation of the true biological signals which were identified in transcriptional data.

### Comparisons between the top EnTDefs and other enhancer-gene pair datasets using GSE

To compare the GSE performance between the top EnTDef and the seven aforementioned datasets, the “nearest_all” method was added in each of the datasets and the same GSE evaluation method was applied as described above in the “Evaluation of enhancer-target gene definitions” section. We also tested the union of the top EnTDef with each of the other 7 datasets (combined datasets). The F1 scores of the 87 evaluation ChIP-seq datasets were calculated and compared by Wilcoxon signed-rank test between the best EnTDef and one of the comparative datasets, or the combined dataset (best EnTDef + dataset). The best CT-EnTDef was also compared with the alternative datasets of the same cell type using the same method as described in the “Evaluation of CT-EnTDefs” section. Since only RIPPLE and HACER datasets provided cell-type-specific enhancer-gene pairs, the best CT-EnTDef was compared with RIPPLE and HACER data subsets specific to GM12878, H1hESC, and K562. For each cell type, the paired F1 scores were compared by Wilcoxon signed-rank test between the best CT-EnTDef and RIPPLE/HACER or the combined CT-dataset (best CT-EnTDef + RIPPLE and best CT-EnTDef + HACER) for that cell type.

### Testing for functions that have significantly more or fewer interceding genes between enhancers and their target genes

We investigated the number of interceding genes between an enhancer and its target gene(s) (i.e., genes between the entire region of an enhancer and the target gene in an EnTDef, depicted in Additional file 1: Fig. S8), and ranked all target genes based on their average number of interceding genes. By definition of nearest distance enhancer-target gene assignment (e.g., >5 kb LocDef), the bottom genes with low numbers of interceding genes are most likely to be correctly assigned to their enhancers, while the top ranked genes with high numbers of interceding genes are least likely assigned to their true enhancers. We used the best-performing EnTDef without “nearest_all” addition, defined by DNase-seq plus FANTOM5 enhancers and ChIA, Thurman, and FANTOM5 enhancer-target gene link methods, as an example for this analysis. Gene Ontology (GO) enrichment testing was performed by *LRpath* [59] using GO Cellular Component (CC), Biological Process (GOBP), and Molecular Function (MF) terms of size ranging from 10 to 1000 genes. The rank-based inverse normal transformation (INT) implemented by the *rankNorm* function in R package *RNOmni* [92] was applied to the average number of interceding genes to have approximately normally distributed scores. *LRpath* took the genes that were linked to at least one enhancer and their exponential transformed INT scores as the input (the input scores were log transformed internally by *LRpath* program) and performed logistic regression-based enrichment testing on each GO term. The significant GO terms (FDR < 0.05) with positive coefficients indicate the functions enriched in genes with less interceding genes (lower ranked), while those with negative coefficients are functions enriched in genes with more interceding genes (higher ranked). For reporting purposes, we filtered out closely related GO terms, using the *GO.db* R package [93] to determine relationships among significant terms. A GO term was filtered if one or more of its parents, children, or siblings had a higher rank in the list [94].

To determine the robustness of the results, we performed the same analysis for all top 10 best-performing EnTDefs without “nearest_all” addition. The enrichment results were combined across EnTDefs for each GO term by taking the Harmonic Mean (HM) *p*-values [95]. The significant terms were extracted using FDR-adjusted HM *p*-values (HM FDR < 0.05), followed by redundant term filtering as described above.

## Supplementary Information

**Additional file 1: Figure S1**. Illustration of different types of Locus Definitions (LocDefs) used in this study. ChIP-seq peaks (top) are assigned to genes if they are located within a chosen LocDef, including: “nearest TSS”, “<5kb to TSS” and “>5kb to TSS”. **Figure S2**. Bar plots of *F1 scores* for each cell type and TF among the evaluation and testing ChIP-seq data sets. Each panel represents one of the top 10 EnTDefs. Cell types in the evaluation dataset are greyish, while those in the testing dataset are bluish. **Figure S3**. Characteristics of testing ChIPseq dataset and the performance of EnTDef on a completely different ChIPseq dataset. (A) Boxplots of the number of peaks in the evaluation and testing ChIP-seq data. (B) The correlation between the number of peaks in evaluation/testing ChIP-seq datasets (log2 scale) and the average *F1 score* across the top 10 best EnTDefs. (C) Scatter plot of average AUPRC (aura under the precision-recall curve) vs AUROC (aura under the receiver operating characteristic curve) of the top 10 EnTDef, top 10 EnTDef_plus5kb and baseline locus definitions (nearest_tss and 5kb_outside) in the gene set enrichment (GSE) testing on the 31 independent ChIP-seq datasets from 14 transcription factors in 9 cell lines, which were completely different from the ones used in the EnTDef evaluation analysis. **Figure S4**. Correlation of average *F1 scores* for a TF across EnTDefs. (A) The correlation between average F1-scores calculated on a TF in a particular cell type using CT-EnTDefs of the matched cell type (“same-CT.EnTDef” on x-axis) and the ones calculated on the same TF using general EnTDefs (“general.EnTDef” on y-axis). (B) The correlation between average F1-scores calculated on a TF in a particular cell type using CT-EnTDefs of a different cell type (“diff-CT.EnTDef” on x-axis) and the ones calculated on the same TF using general EnTDefs (“general.EnTDef” on y-axis). Each dot represents an average F1-score of a TF across EnTDefs, and each panel is one of four cell types (GM12878, H1HESC, K562 and MCF7) for which the CT-EnTDefs were created and evaluated respectively. **Figure S5**. Correlation of *F1 scores* for each TF and EnTDef pair. (A) The correlation between F1-scores calculated on a TF in a particular cell type using a CT-EnTDef of the matched cell type (“same-CT.EnTDef” on x-axis) and the ones calculated on the same TF using general EnTDefs (“general.EnTDef” on y-axis). (B) The correlation between F1-scores calculated on a TF in a particular cell type using a CT-EnTDef of a different cell type (“diff-CT.EnTDef” on x-axis) and the ones calculated on the same TF using a general EnTDef (“general.EnTDef” on y-axis). (C) The correlation between average F1-scores calculated on a TF in a particular cell type using a CT-EnTDef of the matched cell type (“same-CT.EnTDef” on x-axis) and the ones calculated on the same TF using a CT-EnTDef of a different cell type (“diff-CT.EnTDef” on y-axis). Each dot represents a F1-score of a TF and EnTDef pair, and each panel is one of four cell types (GM12878, H1HESC, K562 and MCF7) for which the CT-EnTDefs were created and evaluated respectively. **Figure S6**. Different views of sensitivity and specificity of the selected EnTDefs and baseline locus definitions as compared to the BENGI dataset. (A) Violin plots of average sensitivity and specificity of EnTDefs (top 10 EnTDefs.plus5kb [EnTDef.top_plus5kb], top10 EnTDefs, middle 10 EnTDefs and bottom 10 EnTDefs) in each type of BENGI datasets. The values of the top1 EnTDef were annotated by a red star and the values of baseline locus definitions (nearest TSS [nearest_tss] and >5kb [5kb_outside]) were annotated by the dashed lines (red: nearest TSS, blue: >5kb). ANOVA tests were performed between top10 EnTDefs and nearest TSS: sensitivity in BENGI with fixed positive/negative ratio (BENGI _fixedRatio): *p* value = 3.92×10^−226^; sensitivity in BENGI with natural positive/negative ratio (BENGI _naturalRatio): *p* value =8.56×10^−237^; specificity in BENGI _fixedRatio: *p* value = 2.28×10^−156^; specificity in BENGI _naturalRatio: *p* value = 4.48×10^−218^ (B) Scatter plots of overall false positive rate (1-specificity) vs. true positive rate (sensitivity) of each type of EnTDefs and baseline locus definitions in BENGI _fixedRatio and BENGI _naturalRatio dataset separately. **Figure S7**. Comparisons of the GSE performance for the top10, middle 10 (ranked at 732 to 741) and bottom EnTDefs in two case studies. (A) Distribution of average AUC values of EnTDefs in each group when recapitulating the biological process changes between IMU and KRT HPV(+) subgroups in head and neck cancer patients which were identified in the transcriptional data generated in parallel. (B) Distribution of average AUC values of EnTDefs in each group when recapitulating the biological process changes by SOX17 overexpression in hemogenic endothelium which were identified in the transcriptional data generated in parallel. **Figure S8**. Illustration of interceding gene definition. The interceding genes were defined as the genes with any part of the gene body falling in the query region (i.e., the genomic regions between the farthest positions of an enhancer and its target gene pair). **Figure S9**. Comparison between Poly-Enrich approximate and exact methods. (A) The representative GSE result of transcription factor (TF) *EGR1* in the K562 cell line: all GO terms are generally concordant with the score test (PE.Approx method) being slightly more conservative, but the depleted GO terms tend to deviate more from the PE.Exact method. (B) The correlation of *F1 score* of the top 19 best EnTDefs between the GSE result derived from the PE.Approx method and that derived from the PE.Exact method. Each dot represents a GSE result of a particular TF using one of the 19 EnTDefs. **Figure S10**. Illustration of positive and negative GOBP terms assigned to TFs by GO with the number of annotated genes ≥ 15 and ≤ 2,000. (A) Positive terms include the lowest level of assigned GOBP (leaf terms) and all of their ancestors; negative terms include the terms outside of positive ones, excluding the leaf terms and their siblings and offspring of the assigned terms. (B) More conservative sets of positive and negative terms: positive terms include assigned leaf terms and their parent and grandparent terms, excluding the ones assigned by IEA (automatically assigned, inferred from Electronic Annotation); negative terms include the terms outside of the positive ones, excluding the ancestors of positives, siblings of positives’ ancestors, and offspring of positives.

**Additional file 2: Table S1**: Overview of the top 19 EnTDefs, including the ranks, enhancer/enhancer-gene link methods, and basic summary statistics. **Table S2**: The 31 ENCODE ChIP-seq datasets from 9 completely different cell lines and 14 completely different transcription factors. **Table S3**: The nine ChIA-PET datasets used for generating cell-type-specific EnTDefs (CT-EnTDefs) and number of TFs assayed by ENCODE ChIP-seq in each particular cell type, which were used to evaluate the performance of the CT-EnTDefs. **Table S4**: Overview of the seven independent datasets used for the comparative analysis. **Table S5**: ChIA-PET datasets used by “ChIA” and “Loop” methods to assign enhancer to target genes in a cell-type independent manner (general EnTDefs). **Table S6**: The 87 ENCODE ChIP-seq datasets used for EnTDef evaluation (evaluation ChIP-seq) (tab 1) and the TF vs. cell type matrix (tab 2). **Table S7**: The 13 ENCODE ChIP-seq datasets from 4 different cell lines (testing ChIP-seq).

**Additional file 3.** Peer review history.

## Data Availability

The top-performing EnTDef and EnTDef.plus5kb have been included in the Bioconductor package *chipenrich* [42] and the ChIP-Enrich website (chip-enrich.med.umich.edu). The peak-to-gene assignment functionality provided by our GSE Suite (gsesuite.dcmb.med.umich.edu) allows users to select all possible combinations of enhancer and/or enhancer-to-gene link methods (as described in this study) and obtain the gene assignments for user uploaded genomic regions based on the selected sources and methods. Genomic regions can also be assigned to target genes based on other approaches (e.g., promoters, exons, or anywhere in the genome using the nearest distance method).
