# Supplementary figures and images for: Comprehensive enhancer-target gene assignments improve gene set level interpretation of genome-wide regulatory data
Source: Genome Biol. 2022 Apr 26;23:105. doi: 10.1186/s13059-022-02668-0 (PMC9044877; doi:10.1186/s13059-022-02668-0)

Fig S1

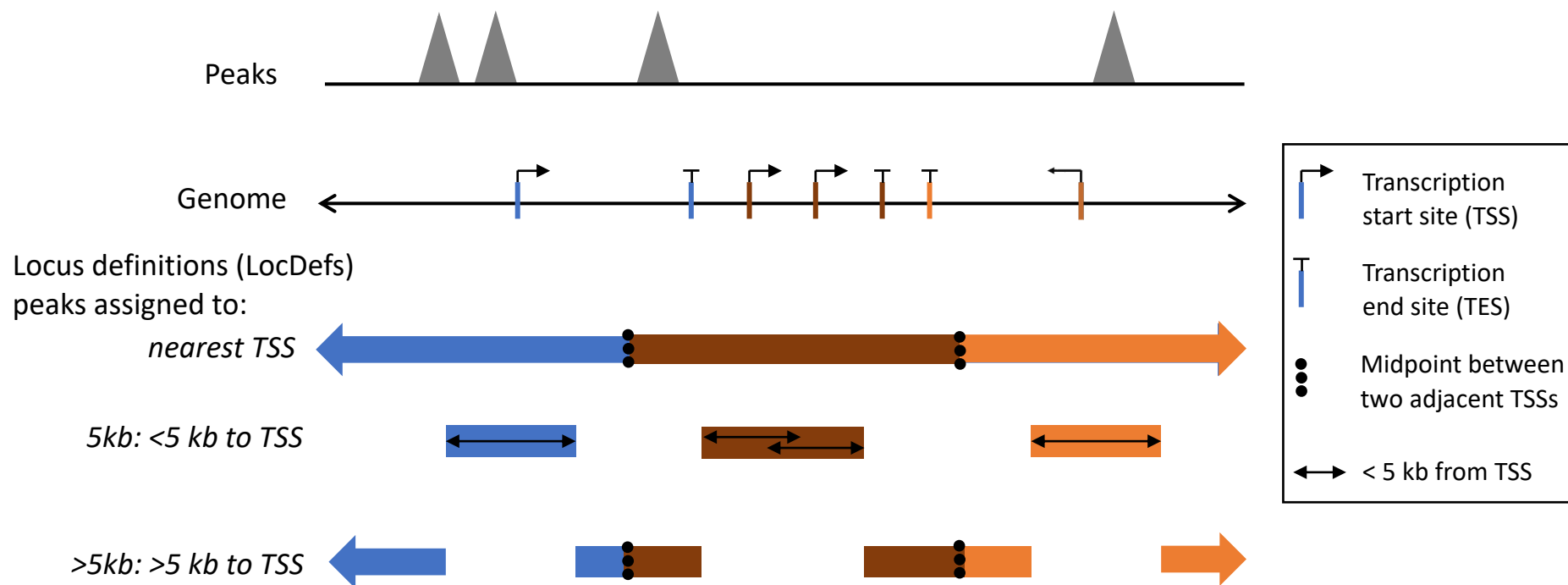

Validation of top 10 enhancer ldefs on new cell lines

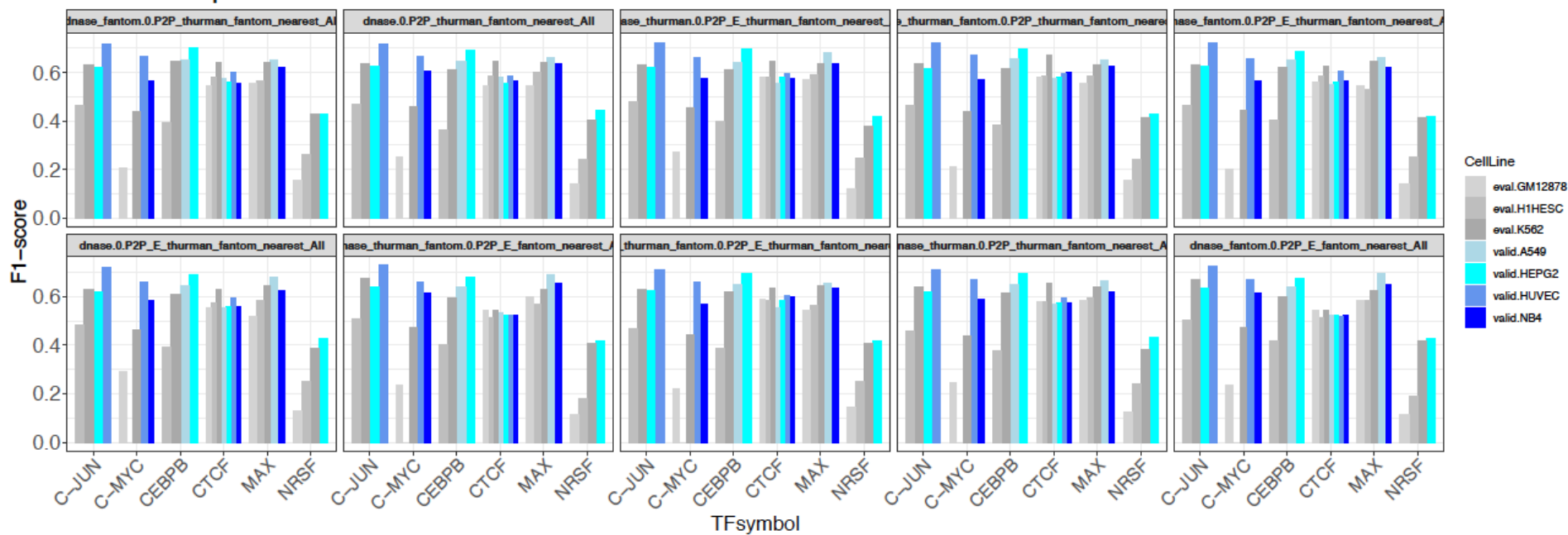

(A)

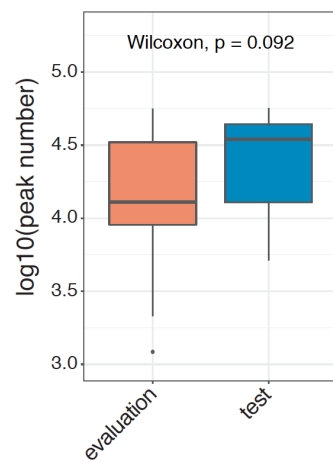

(B)

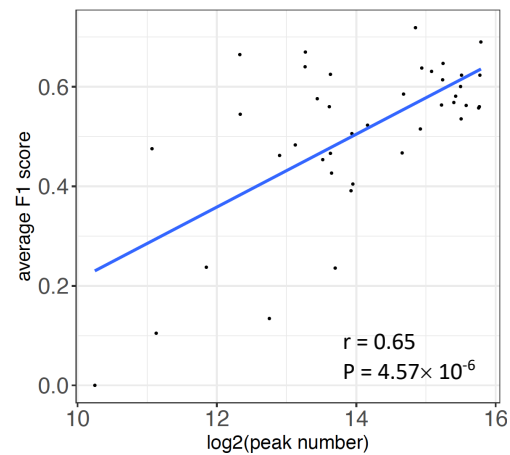

(C)

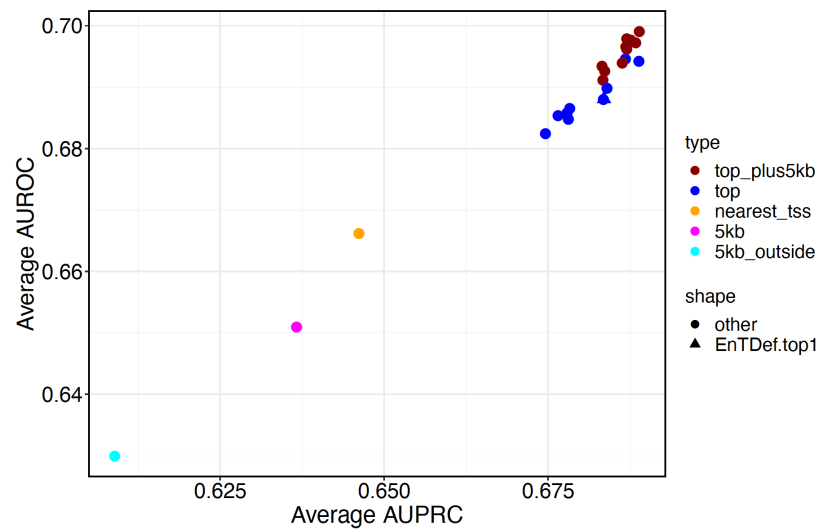

(A)

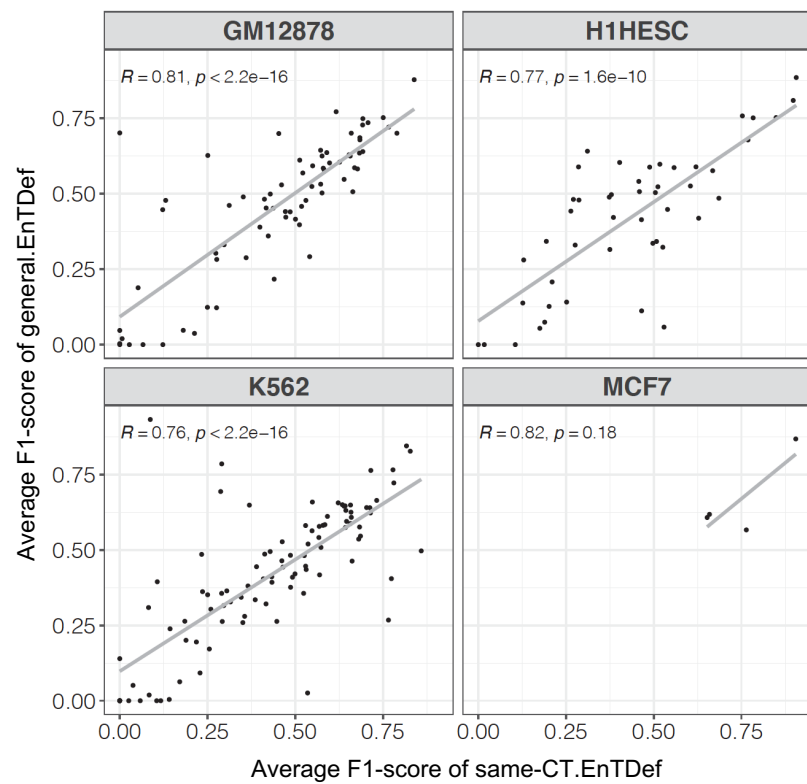

(B)

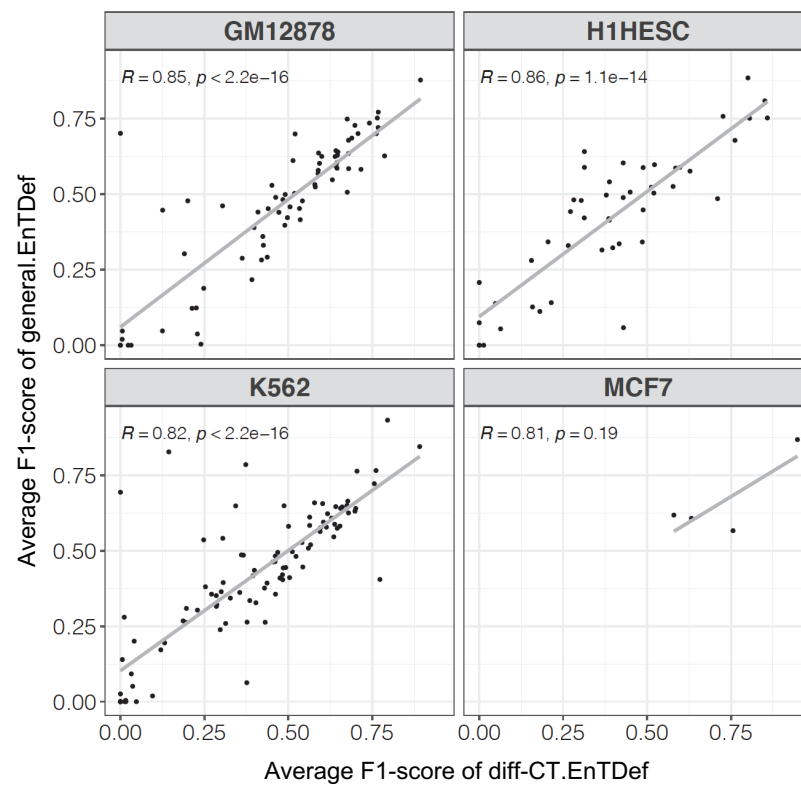

(A)

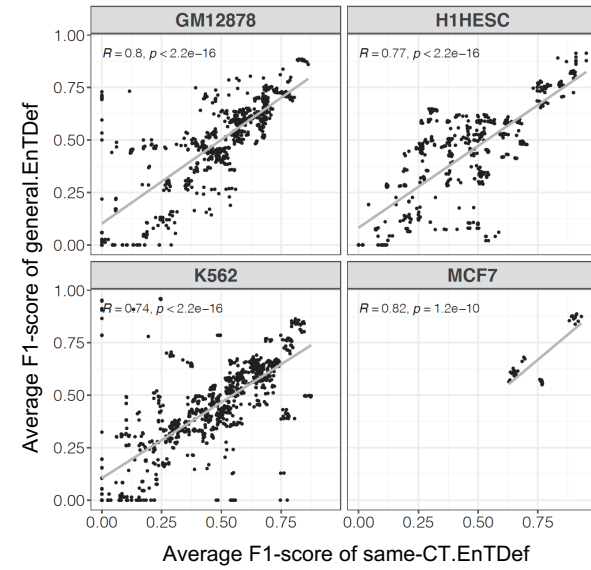

(B)

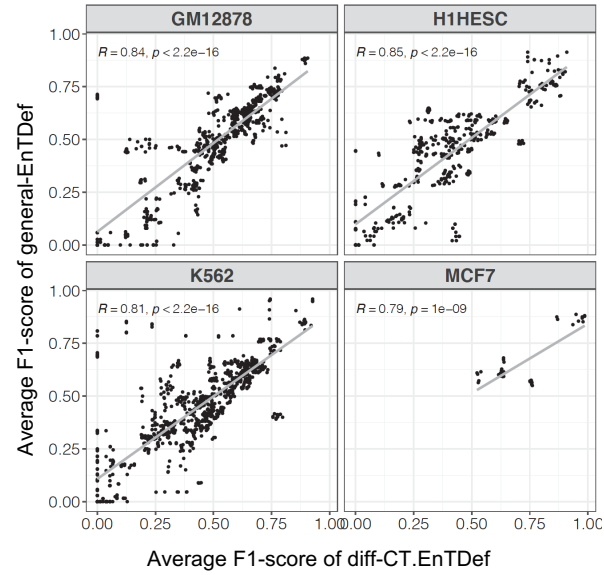

(C)

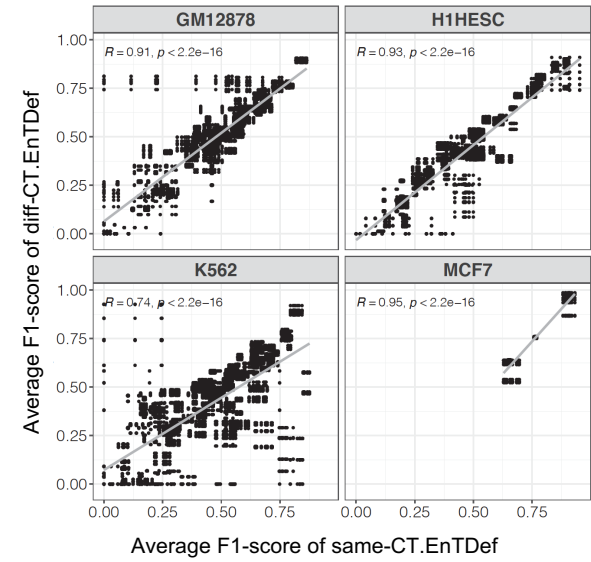

(A)

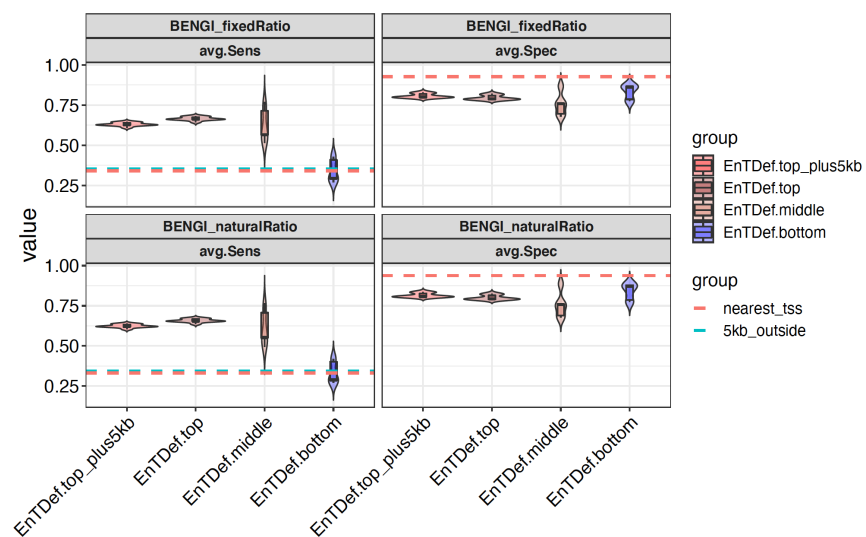

(B)

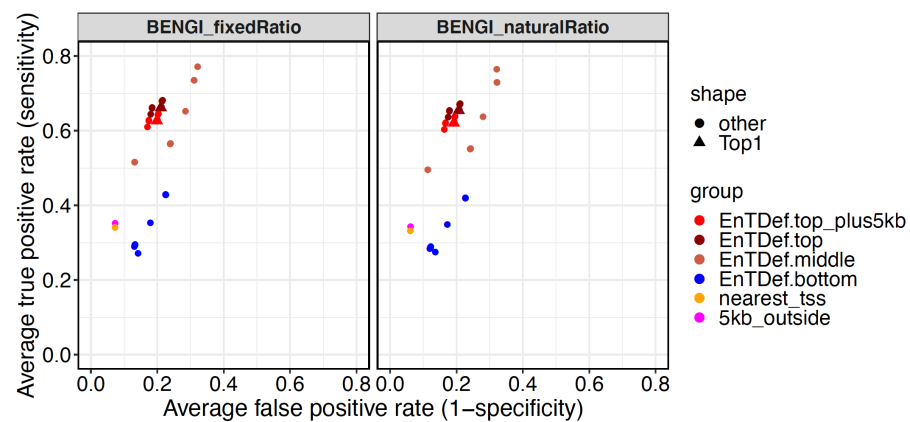

(A)

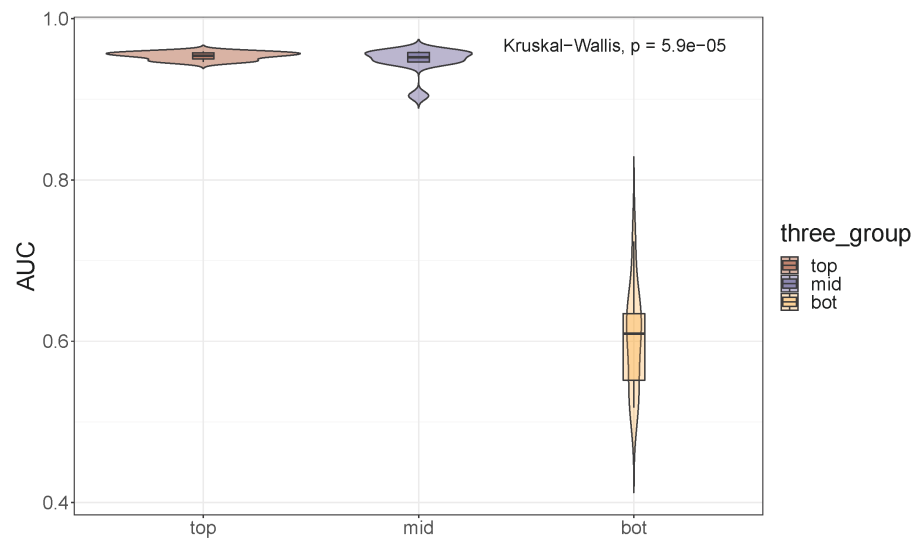

(B)

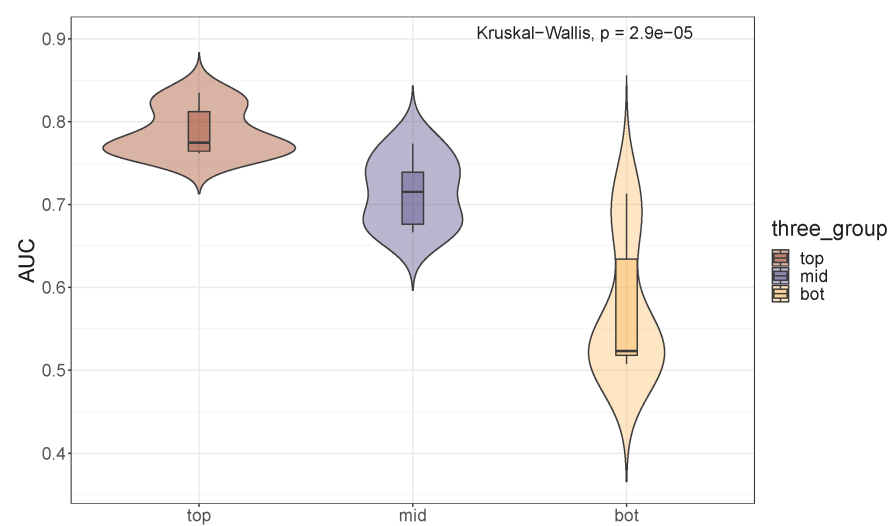

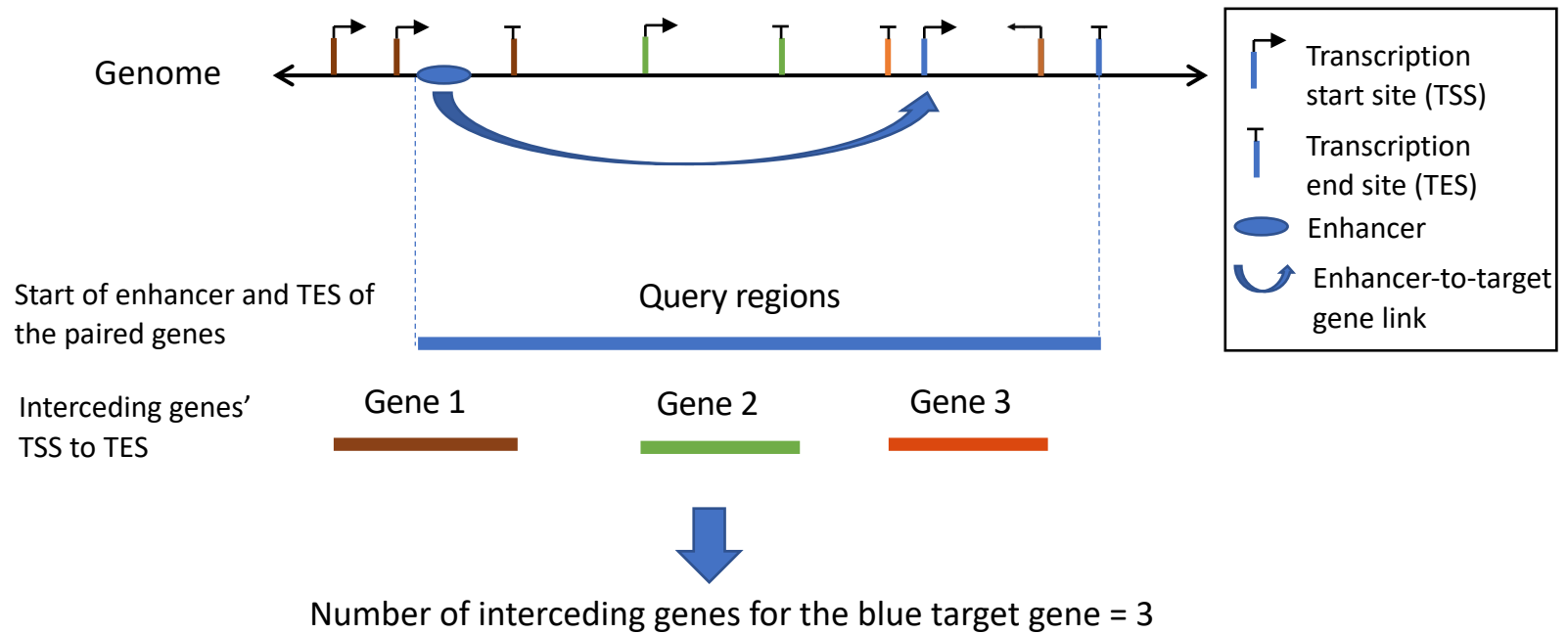

(A)

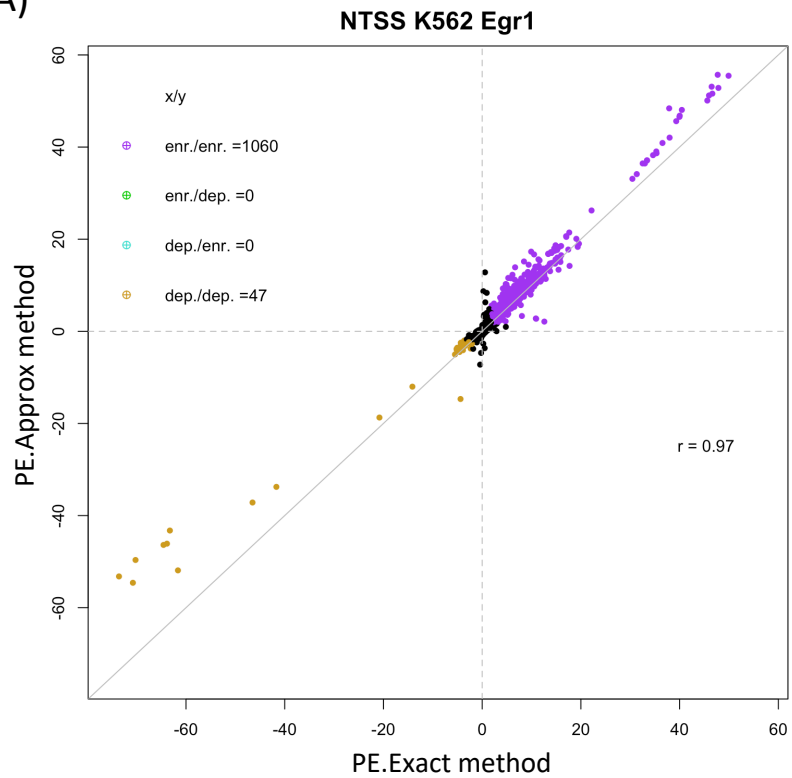

(B)

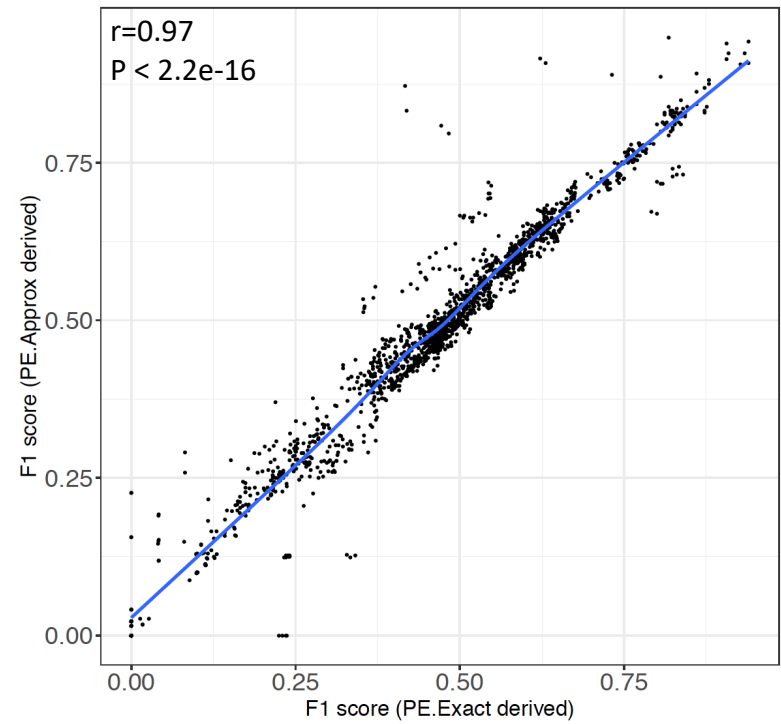

(A)

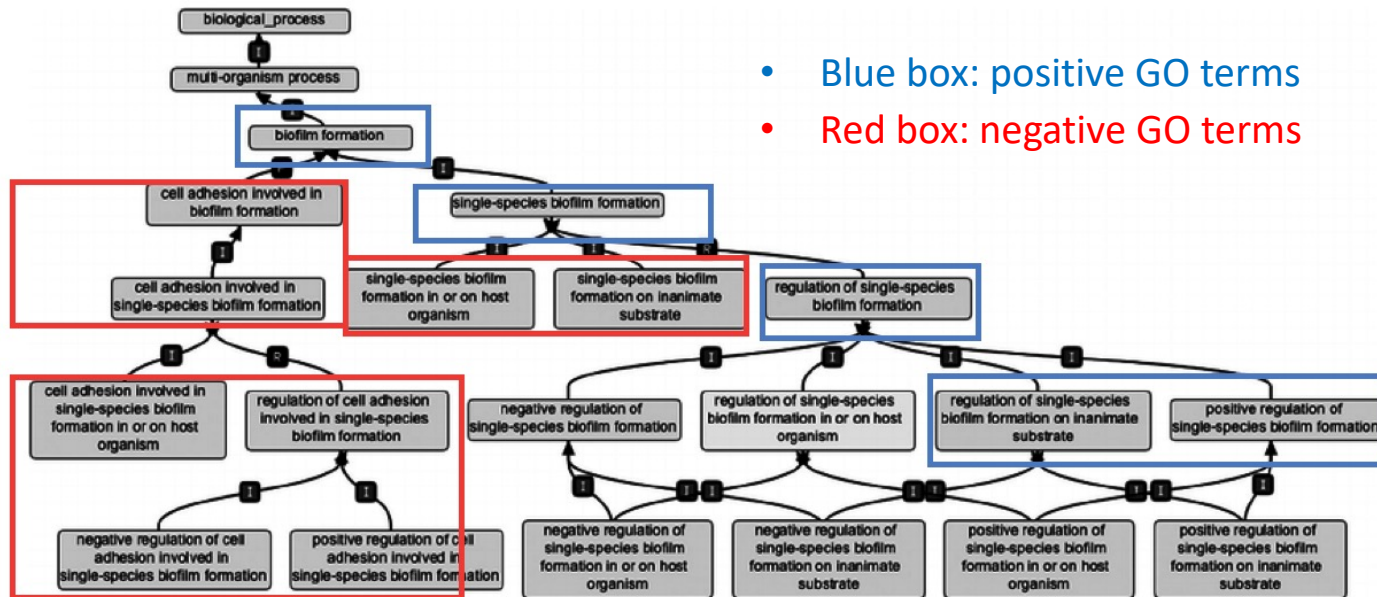

(B)

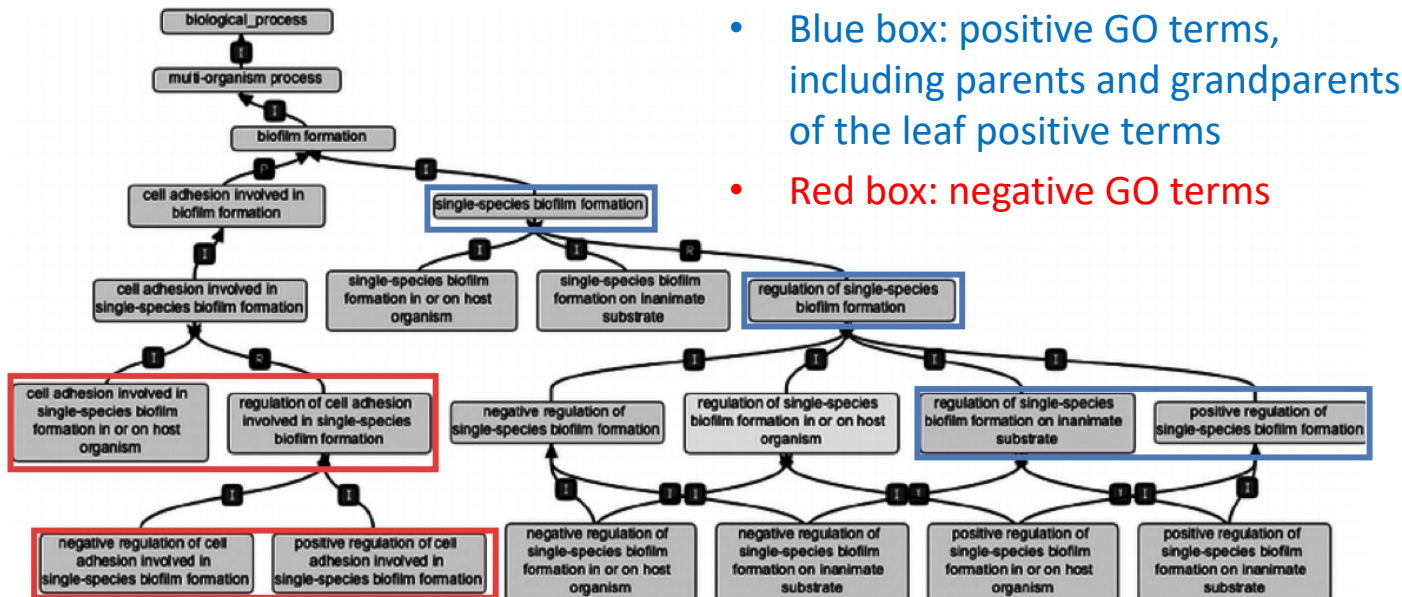

Supplement: Supplementary file 1 — Additional file 1: Figure S1. Illustration of different types of Locus Definitions (LocDefs) used in this study. ChIP-seq peaks (top) are assigned to genes if they are located within a chosen LocDef, including: “nearest TSS”, “<5kb to TSS” and “>5kb to TSS”. Figure S2. Bar plots of F1 scores for each cell type and TF among the evaluation and testing ChIP-seq data sets. Each panel represents one of the top 10 EnTDefs. Cell types in the evaluation dataset are greyish, while those in the testing dataset are bluish. Figure S3. Characteristics of testing ChIPseq dataset and the performance of EnTDef on a completely different ChIPseq dataset. (A) Boxplots of the number of peaks in the evaluation and testing ChIP-seq data. (B) The correlation between the number of peaks in evaluation/testing ChIP-seq datasets (log2 scale) and the average F1 score across the top 10 best EnTDefs. (C) Scatter plot of average AUPRC (aura under the precision-recall curve) vs AUROC (aura under the receiver operating characteristic curve) of the top 10 EnTDef, top 10 EnTDef_plus5kb and baseline locus definitions (nearest_tss and 5kb_outside) in the gene set enrichment (GSE) testing on the 31 independent ChIP-seq datasets from 14 transcription factors in 9 cell lines, which were completely different from the ones used in the EnTDef evaluation analysis. Figure S4. Correlation of average F1 scores for a TF across EnTDefs. (A) The correlation between average F1-scores calculated on a TF in a particular cell type using CT-EnTDefs of the matched cell type (“same-CT.EnTDef” on x-axis) and the ones calculated on the same TF using general EnTDefs (“general.EnTDef” on y-axis). (B) The correlation between average F1-scores calculated on a TF in a particular cell type using CT-EnTDefs of a different cell type (“diff-CT.EnTDef” on x-axis) and the ones calculated on the same TF using general EnTDefs (“general.EnTDef” on y-axis). Each dot represents an average F1-score of a TF across EnTDefs, and each panel is [file 13059_2022_2668_MOESM1_ESM.pdf]
