# Supplementary material for: Comprehensive enhancer-target gene assignments improve gene set level interpretation of genome-wide regulatory data
Source: Genome Biol. 2022 Apr 26;23:105. doi: 10.1186/s13059-022-02668-0 (PMC9044877; doi:10.1186/s13059-022-02668-0)
Supplement: Supplementary file 3 — Additional file 3. Peer review history. [file 13059_2022_2668_MOESM3_ESM.docx]

**Review history**

**First round of review**

**Reviewer 1**

The manuscript "Comprehensive enhancer-target gene assignments improve gene set level interpretation of genome-wide regulatory data" by Tingting Qin and colleagues focuses on identifying gene targets of distal regulatory elements in the human genome. The authors propose generating 1860 distal Enhancer to Target gene Definitions (EnTDefs) across the human genome, by integrating available data of enhancer locations and enhancer-target gene links from different methods across multiple cell types. They evaluated these EnTDefs based on their performances in gene set enrichment (GSE) testing with ChIP-seq data of 34 distinct transcription factors (TFs). F1 scores were used to quantify the performance of EnTDefs by comparing the results from Gene Ontology Biological Processes (GO BP) enrichment testing and the GO databases through all the TFs. The authors showed that their top EnTDefs outperformed the nearest TSS assignment methods with significantly higher average F1 scores. They observed that general EnTDefs, which were derived from combined datasets in multiple cell types, had comparable performance to the cell-type-specific EnTDefs (CT EnTDefs) derived from a particular cell type. They also demonstrated that assignments of certain GO terms by nearest distance method are biased and could lead to incorrect GSE results, which could be correctly identified by their EnTDefs approach.


Major concerns:

The authors showed that little differences are found in the EnTDefs between the general and a specific cell line and among different individual cell lines. Does this conclusion depend on the dataset used for enhancer definition and enhancer-gene linking, that is, will different initial definition lead to different conclusions? Please demonstrate that the datasets used in this study are broad enough to include most scenarios? It might be helpful if authors could provide additional evidence/analyses to address this point.

Is there a difference in enhancer-target gene assignment between housekeeping and cell type-specific genes, in a particular cell line and across different cell lines?

Evaluation of the top EnTDefs performance using additional experimental data should be helpful, such as the chromatin interactions between promoters and regulatory elements. For example, in the recent study of Ren's lab (Nature Genetics, 51, 1442-1449 (2019), PMID: 31501517), they assigned target genes for 27k SNPs.

The authors mentioned multiple different approaches to match the enhancer-gene pairs (line 400). It would be good to have examples showing direct comparison between the best EnTDef and some of these approaches with improved assignment accuracy.

Could the approach in this study be applied to prediction of new/unknown enhancer-target gene identification, by showing some case studies?

Minor points:

Line 172, please show the details of the top EnTDefs, such as enhancer sets and link sets used, number of enhancers/genes involved, number of cell types and so on. It is hard to interpret from Figure 2A. It is not clearly described in the Methods section how these 465 EnTDefs are generated and expand to 1860 EnTDefs using "nearest_all" links.
What is the common and different parts among the EnTDefs with top performance, for example the top 5?
Line 208, how to reject the possibility that outperformance of EnTDef_plus5kbs is mainly the contribution from promoter regions?
There are 87 chip-seq datasets for 34 TFs to calculate F1-scores. How to deal with the redundancy? What is the standard to select these 34 TFs?
Line 516, what is the purpose to add the regions outside the enhancers and promoters using simple "nearest_all" method? How to identify the target gene linked to an enhancer which does not belong to the initial enhancer datasets used to define EnTDefs?

**Reviewer 2**

An important aspect of whole-genome omic (cistromic or epigenomic) profiling today is to map peaks from the profile to associated genes. This is commonly done using the "nearest gene" scheme, whereby a peak is deemed to be relevant to the nearest gene. This allows downstream gene set enrichment analyses to be conducted for any given set of peaks, and is a popular paradigm in bioinformatics. The literature has repeatedly noted that this simplistic scheme may be flawed, since peaks, often found in enhancers, may not be functionally associated with their nearest genes. However, no obvious replacement of this strategy has emerged so far. The authors examine this issue in great detail, systematically examining several reasonable alternatives, and coming up with an objective way to evaluate them. The report some schemes that appear to be generally better and suggest that researchers should use them in place of the nearest TSS scheme. The paper presents sound statistical evidence in favor of the authors' claim.

General comment:

The main concern is that a general claim about enhancer-gene interactions is based on data from a small set of TFs and a highly indirect methodology. The methodology itself stems from the reasonable assumption "TFs tend to the regulate genes in the biological processes to which they belong", but the quantitative extent to which this assumption is reflected in the Gene Ontology annotations is unknown, and deviations of various sorts (e.g., missing or false GO BP annotations) can impact the present study's claims in unknown ways. The evaluation pipeline is quite complex, with many parts, each with its own parameter choices, and this complexity makes the claims of this work harder to judge and accept in generality.

Specific comments:

The authors state that "the fact that these 741 EnTDefs outperformed the >5kb LocDef suggests that the "smart" enhancer to target gene assignments more accurately capture real biological regulatory elements for distal enhancer regions when compared to the simplistic assignment to nearest genes." To me, it seems like a large space of possible "solutions" (enhancer-gene assignment schemes) is examined and about half of them "score" better than the baseline and the other half did worse. This doesn't seem surprising, nor is it in itself convincing evidence that the more complex schemes more accurately capture real biology.

It is hard to understand the significance or meaning of the claims made in lines 176 - 185, characterizing properties of the top 741 EnTDefs. For instance, the "L" method based on loop boundaries with convergent CTCF motifs was used only 26% times. Does that argue that using this method is a bad idea somehow, and does not accurately capture enhancer-gene interaction biology? But this claim refers to the set of EnTDefs that supposedly did well (better than the baseline). So, if 26% of those "smart" EnTDefs used the "L" method, then the method must have some merit to it? So then what is the point of the claim?

The authors claim through Fig 2C that "EnTDefs.plus5Kb" outperform the "nearest TSS" scheme. Strictly speaking, this is indeed supported by their data. But another way of looking at the figure is that despite taking the 10 best of a large number of complex schemes, we only get a ~0.05 improvement in the objective score (F1) over a very simple "nearest TSS" scheme. It seems to suggest that the often criticized nearest-TSS scheme is not so bad after all. The section ends with a rather unsurprising observation about "importance of accurately modeling regulation from enhancers" and that "enhancers have the potential to provide more regulatory information than promoters". It is good to see this widely believed claim supported by the authors' work.

How does the "nearest TSS" scheme's average F1 score vary between the evaluation set of cell lines and the test set? What does the answer to this question say about the observations around Figure 2D?

It is indeed good to see that the "general"EnTDefs are as accurate as cell type-specific EnTDefs in the authors' evaluations. It is also interesting to see the analysis of Figure 4A,B - that when an enhancer-gene relationship jumps over many interceding genes, such relationships tend to pertain to genes of certain GO categories.

The analysis of Figure 5A and 5B should be done using "nearest TSS" as baseline rather than the ">5 Kb LocDef", since the former is a more commonly used strategy for assigning peaks to genes, even though the difference from EnTDefs is only in the distal enhancers. The analysis should also report (at least the count of) the false positive and false negative GO terms called by the EnTDef (e.g., the best EnTDef) where the nearest TSS made the right call. (I hope this suggestion makes sense - at this point the analysis is rather contrived and it is hard to have all the facts of the analysis straight in one's mind.)

In addition to the statistical evidence in favor of the new EnTDef schemes, the paper also presents several GO enrichment analyses that are somewhat tangential to the main point of the work and it is sometimes hard to understand the take-home message from those analyses.

**Authors’ response to reviewers**

**Reviewer #1:** The manuscript "Comprehensive enhancer-target gene assignments improve gene set level interpretation of genome-wide regulatory data" by Tingting Qin and colleagues focuses on identifying gene targets of distal regulatory elements in the human genome. The authors propose generating 1860 distal Enhancer to Target gene Definitions (EnTDefs) across the human genome, by integrating available data of enhancer locations and enhancer-target gene links from different methods across multiple cell types. They evaluated these EnTDefs based on their performances in gene set enrichment (GSE) testing with ChIP-seq data of 34 distinct transcription factors (TFs). F1 scores were used to quantify the performance of EnTDefs by comparing the results from Gene Ontology Biological Processes (GO BP) enrichment testing and the GO databases through all the TFs. The authors showed that their top EnTDefs outperformed the nearest TSS assignment methods with significantly higher average F1 scores. They observed that general EnTDefs, which were derived from combined datasets in multiple cell types, had comparable performance to the cell-type-specific EnTDefs (CT EnTDefs) derived from a particular cell type. They also demonstrated that assignments of certain GO terms by nearest distance method are biased and could lead to incorrect GSE results, which could be correctly identified by their EnTDefs approach.


**Reviewer #1:** Major concerns:

1. The authors showed that little differences are found in the EnTDefs between the general and a specific cell line and among different individual cell lines. Does this conclusion depend on the dataset used for enhancer definition and enhancer-gene linking, that is, will different initial definition lead to different conclusions? Please demonstrate that the datasets used in this study are broad enough to include most scenarios? It might be helpful if authors could provide additional evidence/analyses to address this point.

*Response to reviewer:*

We thank the reviewer for these insightful questions. To address this, we compared our best EnTDef with other general and cell-type specific enhancer-to-gene assignment datasets, including 5 machine learning based approaches (FOCS[1], GeneHancer[2], JEME[3], PEGASUS[4, 5], and RIPPLE[6]), an atlas of Human Active Enhancer to Regulatory variants (HACER)[7] and the experimental dataset from Bing Ren’s lab (RB) mentioned in comment #3 below [8]. These additional datasets were generated either from computationally derived target genes for the known (experimentally validated) enhancers, or from experimentally validated enhancers and/or their paired genes (4C, Hi-C, eQTL, etc.). For each of the seven datasets, we excluded the regions within the 5kb of a TSS from the enhancers and merged the overlapped enhancers. For a fair comparison with our best EnTDef, the “nearest_all” addition was added into the processed enhancer-gene pairs of those datasets. In addition, each of the datasets were incorporated into the best EnTDef as combined datasets. This resulted in 14 new enhancer-gene pair datasets. The GSE performance of these datasets was investigated using F1-scores on the 87 ENCODE TF ChIP-seq peak dataset as we discussed in the main text. The best EnTDef significantly outperformed all the comparative datasets, with the performance of most combined datasets closer to that of best EnTDef (**Revised Figure 5A**).

For the cell-type specific definitions (CT-EnTDefs), we took the cell type-specific datasets from HACER and RIPPLE (the other methods did not offer any cell-type specific datasets for the same cell types as we used). We created 12 CT-specific enhancer-gene pair datasets as described above, including four types of datasets (HACER, HACER + best CT-EnTDef, RIPPLE, and RIPPLE + best CT-EnTDef) in each of the GM12878, H1HESC, and K562 cell types. The same GSE evaluation method was used to access the performance of those CT-specific pairs. Similar to our top EnTDef, our best CT-EnTDef performed significantly better than the new datasets, and the combined ones were comparable to our best CT-EnTDef (Revised Figure 5B). These findings suggest that the datasets that our best EnTDef currently incorporate are sufficiently comprehensive to capture the real biological enhancer-gene interactions, and due to the overlap and/or limited scale of the new datasets, the integration of other datasets into our best EnTDef failed to further improve the biological interpretation of the regulome data.

In addition, the F1-score comparison between the general vs. cell type-specific datasets showed that both cell type-specific datasets (HACER and RIPPLE) significantly outperformed their general counterparts in the GM12878 cell line, especially for the GM12878 HACER dataset whose performance was significantly improved (and comparable to that of our top general EnTDef) (**Response to reviewers Figure 1**). Although the difference in other cell types is not significant, the performance of the other cell type-specific datasets tended to be better than the general ones, but still worse than our general EnTDefs. The general EnTDef and the combined datasets (general EnTDef + HACER or +RIPPLE) were comparable to their cell type-specific counterparts in all cell types, indicating that the current conclusion that the general EnTDef can be applied in a cell type-independent manner still holds.

We have added this analysis in the main text as a new section: “**Comparison of the top EnTDef with other enhancer-gene pair datasets using GSE**”.

**Response to reviewers Figure 1.** F1-score comparisons between cell type-specific and general enhancer-gene pair datasets in different cell types (GM12878, H1hESC and K562). The compared datasets include the best EnTDef (“EnTDef”), HACER or RIPPLE (“Other”), and the combined datasets of best EnTDef and HACER or RIPPLE (“Combined”). The other datasets and cell types are denoted in the title of each panel. Wilcoxon rank-sum test p values are denoted.

2. Is there a difference in enhancer-target gene assignment between housekeeping and cell type-specific genes, in a particular cell line and across different cell lines?

*Response to reviewer:*

We thank the reviewer for this interesting question. To address this, we downloaded a housekeeping gene list and cell-type specific gene lists for five different cell lines (K562, MCF7, HepG2, A549, and Hela “enhanced expressed genes”) from HRT Atlas[9] and protein atlas (<https://www.proteinatlas.org/humanproteome/cell/cell+line>)[10]. Our top ranked general EnTDef and the top-ranked cell type-specific EnTDef (CT-EnTDef) for two cell lines (K562, MCF7) were used to compare enhancer-target gene assignment characteristics. We examined the log number of enhancers per gene and the average log distance of enhancers to the transcription start sites of all assigned target genes. The results are visualized in two density histograms and four violin plots (**Response to reviewers Figure 2**), and the summary statistics are shown in the following table (**Response to reviewers Table 1**). The table shows that when using the best general EnTDef excluding “neareset_all”, both the number of enhancers per gene (Num) and their distances from the TSS (Mean_Dist) are higher for housekeeping genes than for cell-type specific genes. This result is also observed, with p-values included, in **Response to reviewers Figure 2A and 2B**.

**Response to reviewers Table 1.** Average log number of enhancers per gene (Mean_Num) and average log distance of enhancers to the transcription start sites of all assigned target genes (Mean_Dist) assigned by the best general EnTDef excluding “neareset_all” addition. The genes were categorized into housekeeping or cell type-specific ones.

| Gene type | Mean Num | Mean_Dist |
| --- | --- | --- |
| Housekeeping | 5.51998 | 19.9769 |
| A549 | 5.17188 | 19.2445 |
| HeLa | 4.72219 | 19.4747 |
| HepG2 | 5.09463 | 19.2561 |
| K-562 | 4.77969 | 19.1215 |
| MCF7 | 5.47661 | 19.396 |

However, we wondered if this difference could be due to the more comprehensive coverage of enhancers for housekeeping genes since they have many more chances to be discovered in a general EnTDef that combined data from many cell types. Hence, we also examined housekeeping vs cell-type specific genes using our K562 and MCF7-specific EnTDefs, where enhancers for housekeeping and cell-type specific genes have an equal likelihood of being identified.

In the cell type-specific EnTDefs, the cell-type specific genes have significantly more enhancers (**Response to reviewers Figure 2C and 2E)**. However, there was little to no difference in the distance of these enhancers to their target genes (**Response to reviewers Figure 2E and 2F)**. This suggests that the general EnTDef does have more comprehensive coverage of enhancers for housekeeping genes, as one would expect, but it has no bias in relation to the distance of enhancers to their target genes.

**Response to reviewers Figure 2.** Distributions of number of enhancers per gene (log2 scale; 0.25 was used if the number of enhancers was 0) and the average distance from enhancers to the linked TSS (log2 base pairs between enhancers to gene) for housekeeping genes or cell type-specific genes. The best general EnTDef or cell type-specific EnTDefs, excluding the “nearest_all” addition, were used in the analysis (indicated in the titles). The genes were categorized into housekeeping and cell type-specific ones denoted by different colors. Wilcoxon sum-rank tests were performed between housekeeping genes and the cell type-specific ones and the p values were denoted. (A) and (B): Density plots of the number of enhancers assigned to each gene (A) and the average distance of enhancers to the TSSs of all assigned target genes (B). (C)-(F): Violin plots of the number of enhancers per gene among housekeeping genes or K562 (C) and MCF (E) specific genes; violin plot of the average distance of enhancers to the TSSs of all assigned target genes among housekeeping genes or K562 (D) and MCF (F) specific genes.

3. Evaluation of the top EnTDefs performance using additional experimental data should be helpful, such as the chromatin interactions between promoters and regulatory elements. For example, in the recent study of Ren's lab (Nature Genetics, 51, 1442-1449 (2019), PMID: 31501517), they assigned target genes for 27k SNPs.

*Response to reviewer:*

We thank the reviewer for this suggestion. To address this, we used the data suggested from Ren’s lab (RB dataset)[8] as well as another experimental dataset from HACER[7], and compared the enhancer-gene pairs in RB or HACER datasets with our EntDefs (using our top 10, middle 10 that performed similar to the nearest TSS (ranked at 732 - 741), and bottom 10). The number of enhancer-gene pairs with overlapped enhancers (at least 1 bp overlap) and the same assigned genes were counted as the shared interactions between the comparative datasets. The overlap coefficient , i.e. the number of enhancer-gene pairs shared between two datasets divided by the number of pairs in the smaller dataset (as defined and used by Moore et al[11]), was used to rank the EnTDefs in descending order. The results showed that our Top EnTDefs outperformed the Middle EnTDefs, which outperformed the bottom EnTDefs, and their original F1 score-based ranks were highly correlated with the overlap coefficient-based ones as compared to both HACER (Pearson’s R = 0.86, p = 7.2×10^-10^) and RB (Pearson’s R = 0.76, p = 4.4×10^-6^) datasets (Revised Figure 4D).

Moreover, we compared the same set of 30 EnTDefs with a curated enhancer-gene interaction benchmark dataset (BENGI), which provides a gold standard dataset of both true positive and true negative enhancer-target pairs[11] (please see Reviewer #3, point 1). The top 10 EnTDefs showed the largest difference between the percentage of positive vs. negative pairs, and the difference decreased in the middle and bottom 10 EnTDefs sequentially (Revised **Figure 4A**). The ranks based on the difference were significantly correlated with the original F1 score-based ranks (Pearson’s correlation = 0.9, p < 0.0001, **Revised Figure 4B**).

In addition, we compared the GSE performance of our best EnTDef with the RB dataset, and showed that our best EnTDef significantly outperformed the RB dataset (**Revised Figure 5A**). Notably the performance of the RB dataset by itself was poorer than other datasets, and incorporation of the RB dataset into the best EnTDef reduced the performance further. This indicates that this capture Hi-C experiment-derived enhancer-gene pair dataset of 27 human cell/tissue types for 27k SNPs is not sufficiently comprehensive to capture the general interactions across cell types. Similarly, our best EnTDef significantly outperformed HACER and the combined datasets (best EnTDef + HACER) (Revised Figure 5A).

4. The authors mentioned multiple different approaches to match the enhancer-gene pairs (line 400). It would be good to have examples showing direct comparison between the best EnTDef and some of these approaches with improved assignment accuracy.

*Response to reviewer:*

We thank the reviewer for bringing up this question. To address this, we compared our top 10, middle 10 (ranked at 732 to 741) and bottom 10 EnTDefs with five computationally-derived datasets, including FOCS[1], GeneHancer[2], JEME[3], PEGASUS[4, 5], and RIPPLE[6]). A supplementary table (**Supplementary Table S3**) has been added containing the numbers of enhancers and enhancer-target pairs for each. The direct overlap comparison by overlap coefficients (defined and used by Moore et al[11]) showed that the overlap coefficient-based ranks of the 30 EnTDeFs were significantly correlated with their original F1 score-based ranks in four (FOCS, GeneHancer, JEME and RIPPLE) out of the five computationally-derived datasets (Pearson’s correlation ranges from 0.6 to 0.9, p < 0.0001, Figure 4C), and the top ranked EnTDefs had higher overlap coefficients with FOCS, GeneHancer, and JEME (see Response to reviewers Figure 3). The GSE performance comparison also showed that our best EnTDef outperformed the five computational-derived datasets (**Revised Figure 5A and B**).

**Response to reviewers Figure 3**. Violin plots of the overlap coefficients between the top 10, middle 10 (ranked at 732 to 741) and bottom 10 EnTDefs with five computationally-derived datasets.

5. Could the approach in this study be applied to prediction of new/unknown enhancer-target gene identification, by showing some case studies?

*Response to reviewer:*

This is an interesting question. The aim of this study is to leverage all available enhancer-to-target gene assignments using current state-of-the-art knowledge from experiments/correlation analyses across various cell types, and identify the best assignments that can appropriately interpret genome-wide regulome data. It is therefore not capable of predicting completely novel enhancer-target gene pairs. However, it is possible to identify novel interactions in new cell types, i.e. enhancer-gene pairs known from a different cell type, then found in a new cell type to contribute to the enrichment of a pathway. This type of inference could indeed generate novel hypotheses in specific cell types, but would require experimental validation.

Minor points:

6. Line 172, please show the details of the top EnTDefs, such as enhancer sets and link sets used, number of enhancers/genes involved, number of cell types and so on. It is hard to interpret from Figure 2A. It is not clearly described in the Methods section how these 465 EnTDefs are generated and expand to 1860 EnTDefs using "nearest_all" links.

*Response to reviewer:*

We are sorry for the confusion, and we now include the information about the top 19 EnTDefs that showed comparable performance in the Supplementary Table S1.

We also clarified in the Methods section how the 465 EnTDefs were generated and expanded to totally 1860 EnTDefs (line 511): “All possible combinations of the enhancer definition datasets (n=4) and enhancer-gene pair datasets (n=4), allowing multiple at a time, defined 465 of the Enhancer to Target gene Definitions (EnTDefs), i.e. the combinations of 15 enhancer definitions (4C4 + 4C3 + 4C2 + 4C1 = 15; C denotes “choose”) and the 31 enhancer-gene pair methods, which include the combinations without “L” method (3C3 + 3C2 + 3C1 = 7), the combinations with L1/L2/L3 ($7 \times3=21$), as well as the L1/L2/L3 method only (n=3), resulting the $15 \times31=465$ core EnTDefs. In addition, to increase the genome coverage, we tested extending the enhancer regions to 1kb (i.e. “enhancer extension”, 500 bp extension at both sides of the midpoint) (i.e. the “enhancer extension”, the number of EnDefs:), and assigning regions outside of enhancers and promoters (within 5kb of a TSS) to the gene with the nearest TSS (i.e. “nearest_all” addition). The additional combinations using these options brought us to a total of 1,860 distinct EnTDefs. Specifically, extending the enhancer regions of the 465 core EnTDefs resulted in 465 more EnTDefs, upon which the option of the “nereast_all” addition doubled the total to arrive at 1860 ($465 \times2 \times2 =1860$)”.

7. What is the common and different parts among the EnTDefs with top performance, for example the top 5?

*Response to reviewer:*

We have added the detailed combinations of the enhancer definitions and enhancer-gene pair datasets of the top 19 EnTDefs which showed comparable performance in the Supplementary Table S1. For the top 5 EnTDefs in particular, the common elements were DNase-seq for enhancer definition; ChIA, Thurman and FANTOM5 methods for enhancer-gene interactions; and “nearest_all” addition; and the different parts are FANTOM5 and/or Thurman (in top 1, 4 and 5 ), and L1 method for enhancer-gene interactions (in top 3 and 4).

8. Line 208, how to reject the possibility that outperformance of EnTDef_plus5kbs is mainly the contribution from promoter regions?

*Response to reviewer:*

We thank the reviewer for bringing up this important question. As shown in Figure 2C, the top 10 EnTDefs outperformed the “5kb” approach by themselves (i.e. ‘smart’ linking of distal regions alone outperforms promoters alone). Including these 5kb promoter regions (5kb) in the EnTDefs (“EnTDefs_plus5kb”) (i.e. using both ‘smart’ linking of distal regions and promoters) improves their performance and is better than the naïve “nearest_TSS” approach using both distal and promoter regions.

This illustrates that promoters and distal regions individually showed moderate performance (worse than the “nearest_TSS” approach), however, their combination significantly improves the overall performance, indicating they both provide independent information not provided by the other. This makes sense, since often we would expect the regulation of a biological process to involve both binding in promoter and distal enhancer regions, and not always for the same genes. Given the outperformance of our top EnTDefs compared to “5kb” (i.e. promoters), it is possible that the regulatory contribution from distal regions (EnTDefs) is greater than that from the promoter regions in general.

9. There are 87 chip-seq datasets for 34 TFs to calculate F1-scores. How to deal with the redundancy? What is the standard to select these 34 TFs?

*Response to reviewer:*

We have now clarified this point. There were no redundant cell-type-specific transcription factor (TF) datasets (i.e. replicates). Rather, the same TF was assessed in up to 3 different cell lines. ChIPseq of the same TF in different cell types might be correlated, but this will not have a big impact on the F1-score. Since the 34 TFs have at least two datasets and over half of them have 3 datasets, no subset of TFs will dominate the results. We included the TF vs. cell type matrix in the 2^nd^ tab of Supplementary Table S4. We selected the 87 ChIP-seq datasets based on the following criteria: 1) performed in at least two ENCODE Tier 1 cell lines (GM12878, K562 and H1-hESC); 2) the ChIP-seq dataset contains greater than 1K TF peaks.

10. Line 516, what is the purpose to add the regions outside the enhancers and promoters using simple "nearest_all" method? How to identify the target gene linked to an enhancer which does not belong to the initial enhancer datasets used to define EnTDefs?

*Response to reviewer:*

We thank the reviewer for pointing out this was unclear. Since the naïve “nearest TSS” method without considering the real enhancer-target gene interactions has been widely accepted in GSE approaches (as noted by Reviewer 2), we intended to compare our EnTDefs with the “nearest TSS” assignments and hypothesized that the experimental data-based EnTDefs can significantly improve the GSE performance. However, the current knowledge about enhancer-target gene interactions is limited to a portion of the distal regions, leading to a significantly lower portion of the total genome covered in the distal enhancer-target pair definitions compared to the “nearest TSS” method. To increase the genome coverage and make a fair comparison, we used the links in the “>5kb” LocDef to assign target genes to the distal regions (>5kb) outside the original enhancer-gene pairs. To our knowledge this is the best objective way to make the gene assignments for the missed distal regions without prior knowledge.


**Reviewer #2:** An important aspect of whole-genome omic (cistromic or epigenomic) profiling today is to map peaks from the profile to associated genes. This is commonly done using the "nearest gene" scheme, whereby a peak is deemed to be relevant to the nearest gene. This allows downstream gene set enrichment analyses to be conducted for any given set of peaks, and is a popular paradigm in bioinformatics. The literature has repeatedly noted that this simplistic scheme may be flawed, since peaks, often found in enhancers, may not be functionally associated with their nearest genes. However, no obvious replacement of this strategy has emerged so far. The authors examine this issue in great detail, systematically examining several reasonable alternatives, and coming up with an objective way to evaluate them. The report some schemes that appear to be generally better and suggest that researchers should use them in place of the nearest TSS scheme. The paper presents sound statistical evidence in favor of the authors' claim. 

General comment:

The main concern is that a general claim about enhancer-gene interactions is based on data from a small set of TFs and a highly indirect methodology. The methodology itself stems from the reasonable assumption "TFs tend to the regulate genes in the biological processes to which they belong", but the quantitative extent to which this assumption is reflected in the Gene Ontology annotations is unknown, and deviations of various sorts (e.g., missing or false GO BP annotations) can impact the present study's claims in unknown ways. The evaluation pipeline is quite complex, with many parts, each with its own parameter choices, and this complexity makes the claims of this work harder to judge and accept in generality.  

*Response to reviewer:*

We thank the reviewer for raising this comment, and we understand the concern. To further evaluate the accuracy and GSE performance of the EnTDefs, we conducted a series of direct comparisons with other enhancer-gene pair datasets, including 5 machine learning based approaches (FOCS[1], GeneHancer[2], JEME[3], PEGASUS[4, 5], and RIPPLE[6]), an experimentally-derived atlas of Human Active Enhancer to Regulatory variants (HACER) 7] and the dataset from Bing Ren’s lab at UCSD (RB) [8] (please see the Response to reviewer #1, point 1, 3 and 4; Revised Figure 4 and 5). We also now include a gold standard benchmarking dataset (BENGI)[11] to evaluate our top/middle/bottom ranked EnTDefs, and found that the ranking of our EnTDefs is highly concordant (please see Response to reviewer #3, point 1). In addition, as added convincing evidence, we applied GSE testing using our top/middle/bottom ranked EnTDefs for a pair of RNA-seq and WGBS (or ATAC-seq) datasets which were generated in the same experiment. Using the RNA-seq GSE results as the gold standard, we evaluated how well those EnTDefs performed in GSE to recapture the same biological processes observed with RNA-seq (please see the Response to reviewer #2, point 1).

Specific comments:

1. The authors state that "the fact that these 741 EnTDefs outperformed the >5kb LocDef suggests that the "smart" enhancer to target gene assignments more accurately capture real biological regulatory elements for distal enhancer regions when compared to the simplistic assignment to nearest genes." To me, it seems like a large space of possible "solutions" (enhancer-gene assignment schemes) is examined and about half of them "score" better than the baseline and the other half did worse. This doesn't seem surprising, nor is it in itself convincing evidence that the more complex schemes more accurately capture real biology.

*Response to reviewer:*

We thank the reviewer for raising this concern. Our goal of this study is to prioritize the generated EnTDefs which leveraged the state-of-the-art knowledge of enhancer-gene assignments across a large number of cell types, and ultimately select one that can provide the best biological interpretation of regulome data. Therefore, the number of EnTDefs that showed better or worse than the baseline is not of concern, but we understand that we did not adequately portray *how much biologically and statistically significant the differences were (as opposed to random fluctuations)*.

We showed that those 741 EnTDefs were statistically significantly better than the baseline (“>5kb”), arguing that this statistical significance must be linked to capturing more real biology. To further prove this, we compared our top 10, middle 10 that performed similar to the nearest TSS (ranked at 732 - 741), and bottom 10 EnTDefs to an experimentally-derived gold standard dataset, and in two case studies with RNA-seq performed in parallel with either WGBS or ATAC-seq.

1) We compared the top10/middle10/bottom10 ranked EnTDefs with a curated enhancer-gene interaction benchmark dataset (BENGI), which provides a gold standard dataset of both true positive and true negative enhancer-target pairs[11] (please see Reviewer #3, point 1). The top 10 EnTDefs showed the largest difference between the percentage of positive vs. negative pairs, and the difference decreased in the middle and bottom 10 EnTDefs sequentially (Revised **Figure 4A**). The ranks based on the difference were significantly correlated with the original F1 score-based ranks (Pearson’s correlation = 0.9, p < 0.0001, Revised **Figure 4B**).

2) We applied GSE testing using the top10/middle10/bottom10 ranked EnTDefs to the two case studies mentioned above: a pair of RNA-seq and WGBS (or ATAC-seq) datasets as described below.

Case study 1: Comparison of two subtypes of HPV-associated head and neck cancer, with the RNA-seq results published in Zhang Y, et al [12] and the differential DNA methylation regions from WGBS data newly uploaded to GEO.

Case study 2: A recent study aiming at identifying SOX17 functionality during hemopoiesis provides RNA-seq and ATAC-seq for samples with overexpression or non-overexpression of SOX17, available at  [GSE140341](https://www.ncbi.nlm.nih.gov/geo/query/acc.cgi?acc=GSE140341)[13]. The RNA-seq data were processed by edgeR and GSE results by RNA-Enrich were used as the gold standard.

As seen in the **Supplementary Figure S6**, the top 10 ranked EnTDefs performed better than the middle 10, which performed better than the bottom 10. This provides additional evidence supporting the accuracy of our F1 score-based ranking of the EnTDefs, using regulatory data types other than ChIP-seq.

2. It is hard to understand the significance or meaning of the claims made in lines 176 - 185, characterizing properties of the top 741 EnTDefs. For instance, the "L" method based on loop boundaries with convergent CTCF motifs was used only 26% times. Does that argue that using this method is a bad idea somehow, and does not accurately capture enhancer-gene interaction biology? But this claim refers to the set of EnTDefs that supposedly did well (better than the baseline). So, if 26% of those "smart" EnTDefs used the "L" method, then the method must have some merit to it? So then what is the point of the claim?

*Response to reviewer:*

We apologize for the confusion. To avoid the misleading statement, we re-analyzed the importance of the methods individually by evaluating the percentage of the EnTDefs with a particular method (for enhancer, extension, or enhancer-target gene link method) that showed significantly increased F1-scores as compared to those removing only that particular method (i.e. pairwise comparisons removing just one variable at a time). Revised Figure 2B has been updated, showing the relative importance of each method as integrated in the EnTDefs, and the methods and main text has been modified accordingly (section “**Overview of the EnTDef characteristics**”, 2^nd^ paragraph, line 176-202). Briefly, the methods used in the best performing EnTDef showed higher ranked contribution, including FANTOM5 and DNase-seq for the enhancer definition methods, and “nearest_all”, FANTOM5 and ChIA ranked as the top 3 for linking to target genes. The majority of EnTDefs without the enhancer extension showed significantly better performance than their 1k bp extension counterparts.

3. The authors claim through Fig 2C that "EnTDefs.plus5Kb" outperform the "nearest TSS" scheme. Strictly speaking, this is indeed supported by their data. But another way of looking at the figure is that despite taking the 10 best of a large number of complex schemes, we only get a ~0.05 improvement in the objective score (F1) over a very simple "nearest TSS" scheme. It seems to suggest that the often criticized nearest-TSS scheme is not so bad after all.  The section ends with a rather unsurprising observation about "importance of accurately modeling regulation from enhancers" and that "enhancers have the potential to provide more regulatory information than promoters". It is good to see this widely believed claim supported by the authors' work.

*Response to reviewer:*

We thank the reviewer for the comment. We agree that the nearest TSS method actually works quite well in GSE testing, and it is a reasonable choice when the real enhancer-gene interactions are not available. However, we indeed proved that including experiment-derived enhancer-gene assignments can significantly improve the GSE performance and lead to better biological interpretation for regulome data.

4. How does the "nearest TSS" scheme's average F1 score vary between the evaluation set of cell lines and the test set? What does the answer to this question say about the observations around Figure 2D? 

*Response to reviewer:*

We thank the reviewer for this thoughtful question. We have added the average F1-score of the “nearest_TSS” method in Revised Figure 2D. The performance of the top 10 EnTDefs and “nearest_TSS” was highly correlated between the evaluation and test ChIP-seq datasets (r=0.94, p = 1.23×10^-5^), and the test datasets showed significantly higher F1 scores for EnTDefs and nearest_TSS (Wilcoxon rank-sum test, p=0.00098). In addition, the top 10 EnTDefs outperformed the nearest_TSS method consistently in both the evaluation and test ChIP-seq datasets. These results suggest that there is an unknown, independent variable explaining at least part of the increased performance of test datasets compared to evaluation datasets, for example, potentially better ChIP-seq pull downs for the test datasets.

5. It is indeed good to see that the "general"EnTDefs are as accurate as cell type-specific EnTDefs in the authors' evaluations. It is also interesting to see the analysis of Figure 4A,B - that when an enhancer-gene relationship jumps over many interceding genes, such relationships tend to pertain to genes of certain GO categories. The analysis of Figure 5A and 5B should be done using "nearest TSS" as baseline rather than the ">5 Kb LocDef", since the former is a more commonly used strategy for assigning peaks to genes, even though the difference from EnTDefs is only in the distal enhancers. The analysis should also report (at least the count of) the false positive and false negative GO terms called by the EnTDef (e.g., the best EnTDef) where the nearest TSS made the right call. (I hope this suggestion makes sense - at this point the analysis is rather contrived and it is hard to have all the facts of the analysis straight in one's mind.)

*Response to reviewer:*

We thank reviewer for the comments. Given the new analyses comparing our approach to other machine learning approaches, the benchmarking analysis using BENGI, the already complicated nature of this paper, and the fact that this specific analysis was somewhat contrived (we agree with the reviewer), we decided to remove this section (“*Nearest distance assignment method leads to false positive and false negative GSE results*”) for the sake of simplicity. We did, however, also perform the analysis of this section using “nearest TSS” as the baseline, and found similar GO terms (e.g. “regulation of chromosome organization”, and “covalent chromatin modification”) to be common false negatives using nearest TSS, and there were much fewer false negatives overall called by our top EnTDef than by nearest TSS (*data not shown*).

6. In addition to the statistical evidence in favor of the new EnTDef schemes, the paper also presents several GO enrichment analyses that are somewhat tangential to the main point of the work and it is sometimes hard to understand the take-home message from those analyses. 

*Response to reviewer:*

We agree with the reviewer that the analysis regarding Figure 5 (section “*Nearest distance assignment method leads to false positive and false negative GSE results*”) is somewhat contrived, so removed this section for the sake of simplicity. Instead, we added new sections (“**Independent validation of our EnTDef ranking approach**” and “**Comparison of the top EnTDef with other enhancer-gene pair datasets using GSE**) comparing our EnTDefs with other enhancer-gene target pair datasets and the benchmarking analysis using an independent “gold standard” dataset, BENGI. We believe these new sections are more easily understandable and more important, because they show (1) that our ranking is meaningful, and (2) that our final top EnTDef outperforms several other alternatives.

Reviewer #3: In this manuscript the authors seek to evaluate enhancer to gene linkages through a benchmark that uses gene set enrichment as a baseline "truth".  Their approach is an attempt at being comprehensive (e.g. every combination they can conceive is tested) but this is a bit heavy handed and leads to difficult to interpret results.  The comparison it GO is an interesting idea, but this manuscript ultimately fall short of both providing a convincing benchmark and of asserting the utility of this strategy.  
Ultimately this reviewer is left wondering what was learned from this exercise. 


1. (*) This paper is a benchmark of a variety of approaches but using an unusual validation strategy: best gene set enrichment.   There is a certain circularity to this and bias — as more well studied systems / TFs will have deeper annotation in GSE.  They don't consider other evaluation techniques or recent benchmarks (such as Moore et. al. 2020), e.g. evaluating this benchmark in light of numerous similar recent efforts is absent.

*Response to reviewer:*

We thank the reviewer for raising this concern. As suggested, we now use the benchmarking dataset “BENGI” [11] to evaluate the enhancer-gene pairs in EnTDefs, illustrating that our ranking approach is working as expected (**Revised Figure 4A and 4B**). In addition, we compared our method with five other computationally-derived methods and two additional experiment-based datasets (please see the Response to reviewer #1, point 1, 3 and 4). We also removed the section that we thought was most difficult to interpret to simplify the already complicated manuscript (section “*Nearest distance assignment method leads to false positive and false negative GSE results*”). The new analyses are included in the main text as sections “**Independent validation of our EnTDef ranking approach**” (**Revised Figure 4**) and “**Comparison of the top EnTDef with other enhancer-gene pair datasets using GSE**” (**Revised Figure 5**). Briefly, our methods significantly outperformed the other datasets and the combined datasets (EnTDef + other dataset) based on different evaluation methods, including direct comparison using overlap coefficients (**Revised Figure 4C and 4D**) and GSE on the 87 evaluation ChIPseq datasets (**Revised Figure 5A and 5B**). We also validated our EnTDef ranking using two case studies comparing GSE on paired RNA-seq and WGBS (or ATAC-seq) datasets generated from the same experiment (**Supplementary Figure S6**).

2. (*) They make a point of saying they tried "ALL" combinations of methods for ... (line 133-134 pg 7), but really they identified 4 papers from the literature and then built every combination of enhancer identification and linkage method across those papers.  They then augment these combinations with some heuristics (extension length, assigning non-enhancers to nearest gene).

*Response to reviewer:*

When we performed this study, those 4 papers were the studies introducing the most comprehensive experiment-based enhancer-target gene datasets. We did “All” possible combinations of the 4 datasets of enhancer definitions (ChromHMM, DNase-seq, FANTOM5 and Thurman), and 4 methods defining enhancer-gene pairs (ChIA-PET interaction, Thurman DNase-signal correlation, FANTOM5 expression correlation and loop boundaries of ChIA-PET with CTCF motifs), with or without enhancer extension (0 or 1k bp), and/or the “nearest_all” addition, resulting in 1,860 EnTDefs (see Methods section “Generation of general enhancer-target gene definitions”, 2^nd^ paragraph). To further evaluate whether the included datasets are broad enough, we now compare our top EnTDef with other datasets, including five machine learning-derived datasets and two experiment-based datasets, and the combined datasets (EnTDef + other dataset) (please see the new added sections “**Independent validation of our EnTDef ranking approach**” and “**Comparison of the top EnTDef with other enhancer-gene pair datasets using GSE**”). The results showed that our methods significantly outperformed the other methods and performed as well or better than the combined datasets (EnTDef + other dataset) based on different evaluation methods, including comparing with the gold standard BENGI, direct enhancer-gene interaction comparison using overlap coefficients, GSE on the 87 evaluation ChIPseq datasets, and GSE on paired RNA-seq and WGBS (or ATAC-seq) datasets generated from the same experiment (**Revised Figure 4 and 5**). This suggests that the datasets included in the study are able to comprehensively capture the current knowledge of enhancer-gene interactions.

3. (*) The caption to Figure 1 suggests they allow multiple definitions for enhancers and gene links (necessary to get to 1860 EnTDefs -- as exclusivity would be closer to 100 distinct combinations).  This introduces structure in their EnTDefs (e.g. many are subsets or combinations of others) which is not ultimately considered in their evaluation metrics.  Aggregation stats ("54% of top ranking included FANTOM5" pg 9) only provide one perspective on what drives these rankings.   Additionally, the definitions in Figure 1 may already overlap -- meaning performance could be driven by specific features or sets of info.   The result is that they struggle to decipher what specifically adds to performance of any specific EnTDef -- and spend tremendous real estate in the paper trying to decipher what (if anything) they actually learned.

*Response to reviewer:*

We thank the reviewer for pointing out the shortcoming of the aggregation analysis deciphering the importance of each method included in the EnTDefs. Since the structure of EnTDefs are correlated and the impact of the combinatory methods on the EnTDefs’ performance is synergistic (we agree with the reviewer), we re-analyzed the relative importance of each method by evaluating the percentage of the EnTDefs with a particular method (e.g., enhancer, extension, or enhancer-target gene link) that showed significantly increased F1-score compared to those removing that method only (please see the response to reviewer #2, point 2; **Revised Figure 2B**). The methods used in the best performing EnTDef showed higher ranked contribution, including FANTOM5 and DNase-seq for the enhancer definition methods, and nearest_all, FANTOM5 and ChIA ranked as the top 3 for linking to target genes. The majority of EnTDefs without the enhancer extension showed significantly better performance than their 1k bp extension counterparts. The main text and methods have been modified accordingly (section “**Overview of the EnTDef characteristics**”, 2^nd^ paragraph, line 176-202).

4. (*) I find the cell type specific vs general comparison unconvincing.  CT specific will be limited by available data whereas general comparisons have more data underlying (including the specific CT being compared to).  A better comparison would be a general model that held out a specific CT compared to that CT's model.

*Response to reviewer:*

We thank the reviewer for the insightful suggestion. For a fair comparison, we now exclude the datasets of the particular cell type from the general EnTDefs, and compared them with the CT-EnTDefs of the same cell type. Figure 3 has been updated and the corresponding text has been modified. Briefly, the updated results are similar to what we observed, and the conclusion that the performance of general EnTDefs are comparable to CT-EnTDefs still holds.

The reviewer is correct, however, that cell type-specific EnTDefs (CT-EnTDefs) are more limited, especially for genes expressed in many tissue types. We see this in the **Response to reviewers Figure 2**, which shows that when using our general EnTDef, housekeeping genes have more enhancers than cell type-specific genes. However, when using a CT-EnTDef, cell-type specific genes have more enhancers.

5. (*) If GSEA makes systematic mistakes, these would be detectable by the frequency with which particular terms are enriched.  For example consider line 302: "and thus most easily detectable by current GSE testing. " If this is true they should show up more often than other terms because of the bias.  So are these commonly disregarded or down weighted by the GSEA-InContext approach which looks at the frequency of particular term enrichments showing up?  Likewise if these are non-random then you'd expect the same results in a distant related species (say mouse) where ample data is available but the genome is not similarly structured (e.g. only syntenic blocks remain).

*Response to reviewer:*

We thank the reviewer for the suggestion. Unfortunately, we cannot use GSEA-InContext weights to assess this, because these weights are determined based on their frequency of detection in gene expression data. While they do show there is bias for which genes are most likely detected as differentially expressed and which are least likely, these bias sources are different from what we have examined using regulome data. The bias we examined is due to how difficult it is to accurately link a gene to their enhancer regions. To identify this bias, GSEA-InContext would have had to use regulatory data such as ChIP-seq or ATAC-seq, rather than RNAseq data to calculate their gene ranks.

6. (*) It is unclear to what extent performance is based on the quality of the datasets being used, particularly for the ChIP comparisons. 

*Response to reviewer:*

We thank the reviewer for raising this interesting question. To evaluate the impact of ChIP-seq quality on the GSE performance of EnTDefs (F1-scores), we investigated the correlation between the F1 and FRiP (fraction of reads in peaks) scores of the ChIP-seq evaluation dataset. FRiP score, measuring the global ChIP enrichment, is an important metric used by ENCODE for ChIP-seq quality control. For each ChIP-seq dataset, we calculated average F1 score of the top 19 EnTDefs that showed comparable performance and compared with their FRiP scores. No significant correlation was shown between the average F1 scores and the FRiP scores across the 87 ChIP-seq evaluation datasets (*R* = 0.2, p = 0.077; **Response to reviewers Figure 4**). In addition, we calculated the Pearson’s correlation (*R*) between the F1 scores and FRiP scores across the 87 ChIP-seq evaluation datasets for each of the 1680 EnTDefs. Overall, F1 scores were somewhat positively correlated with *FRiP scores*, with an average *R* value of 0.22 (ranging from -0.08 to 0.62; the average *R* of the top19 EnTDefs is 0.19). This confirms the reviewer’s suspicion that performance is affected by the quality of the datasets, although not nearly as strongly as one might predict. These findings demonstrate that the ChIP-seq quality only had a limited influence on the GSE performance of the EnTDefs, and the impact is minimal for the top ranked EnTDefs. Furthermore, since we used the average F1 scores across all ChIP-seq datasets to rank the EnTDefs, the influence of ChIP-seq quality on all EnTDefs are the same.

**Response to reviewers Figure 4**. Correlation between the average F1-score among the top 19 EnTDefs and the FRiP scores of ChIP-seq evaluation datasets.

Minor issues:

*Response to reviewer:*

We thank the reviewer for pointing out the unclear statements or grammar errors. We have fixed all in the main text as the reviewer suggested, and replied to the questions as below.

7. (*) pg 2 line 30 "Ontology (GO) annotations., assuming that"   — extra period. 
8. (*) pg 4 line 96: " …enhancer-promoter interactions are still highly restricted to a small number of cell types, which is probed by Chromatin Interaction .."  should be "…enhancer-promoter interactions are still restricted to a small number of cell types, which have been probed by Chromatin Interaction .."
Though later in this same sentence (line 98) it says, "and the genome-wide interaction map is still limited due to the high cost of Hi-C experiments"  — so is this sentence about genome-wide maps or cell type specific maps? *Response to reviewer:* This sentence talks about genome-wide Hi-C assays in general.

9. (*) pg 6 line 109: " .. and most methods simply do the assignment using the nearest genes regardless of the actual regulatory targets .."  is genes really supposed to be plural here?

*Response to reviewer:* We changed it to singular “gene”.

10. (*) line 118: "We also showed that as opposed to being random, target genes that are often missed or often falsely identified using adjacent gene assignments are biased to specific Gene

Ontology terms. "     What does the "are biased to specific gene ontology terms" here refer to?

*Response to reviewer:* As shown in Revised Figure 6, the adjacent gene assignments led to some GO terms being more likely to be assigned (biased to), e.g. the terms listed on the left side of the histogram in Revised Figure 6A, or the terms had less interceding genes listed in the top panel in Revised Figure 6B.

11. (*) line 266: "performed near significantly better than "  how is one "near" significantly better?
*Response to reviewer:* We updated analysis results for this section (“General EnTDefs perform comparably to cell-type-specific EnTDefs”) as the reviewer suggested (point 4), and the text has been updated accordingly. This unclear sentence has been removed.

**References**

1.	Hait TA, Amar D, Shamir R, Elkon R: **FOCS: a novel method for analyzing enhancer and gene activity patterns infers an extensive enhancer-promoter map.** *Genome Biol* 2018, **19:**56.

2.	Fishilevich S, Nudel R, Rappaport N, Hadar R, Plaschkes I, Iny Stein T, Rosen N, Kohn A, Twik M, Safran M, et al: **GeneHancer: genome-wide integration of enhancers and target genes in GeneCards.** *Database (Oxford)* 2017, **2017**.

3.	Cao Q, Anyansi C, Hu X, Xu L, Xiong L, Tang W, Mok MTS, Cheng C, Fan X, Gerstein M, et al: **Reconstruction of enhancer-target networks in 935 samples of human primary cells, tissues and cell lines.** *Nat Genet* 2017, **49:**1428-1436.

4.	Clement Y, Torbey P, Gilardi-Hebenstreit P, Crollius HR: **Enhancer-gene maps in the human and zebrafish genomes using evolutionary linkage conservation.** *Nucleic Acids Res* 2020.

5.	Naville M, Ishibashi M, Ferg M, Bengani H, Rinkwitz S, Krecsmarik M, Hawkins TA, Wilson SW, Manning E, Chilamakuri CS, et al: **Long-range evolutionary constraints reveal cis-regulatory interactions on the human X chromosome.** *Nat Commun* 2015, **6:**6904.

6.	Roy S, Siahpirani AF, Chasman D, Knaack S, Ay F, Stewart R, Wilson M, Sridharan R: **A predictive modeling approach for cell line-specific long-range regulatory interactions.** *Nucleic Acids Res* 2015, **43:**8694-8712.

7.	Wang J, Dai X, Berry LD, Cogan JD, Liu Q, Shyr Y: **HACER: an atlas of human active enhancers to interpret regulatory variants.** *Nucleic Acids Res* 2019, **47:**D106-D112.

8.	Jung I, Schmitt A, Diao Y, Lee AJ, Liu T, Yang D, Tan C, Eom J, Chan M, Chee S, et al: **A compendium of promoter-centered long-range chromatin interactions in the human genome.** *Nat Genet* 2019, **51:**1442-1449.

9.	Hounkpe BW, Chenou F, de Lima F, De Paula EV: **HRT Atlas v1.0 database: redefining human and mouse housekeeping genes and candidate reference transcripts by mining massive RNA-seq datasets.** *Nucleic Acids Res* 2021, **49:**D947-D955.

10.	Uhlen M, Fagerberg L, Hallstrom BM, Lindskog C, Oksvold P, Mardinoglu A, Sivertsson A, Kampf C, Sjostedt E, Asplund A, et al: **Proteomics. Tissue-based map of the human proteome.** *Science* 2015, **347:**1260419.

11.	Moore JE, Pratt HE, Purcaro MJ, Weng Z: **A curated benchmark of enhancer-gene interactions for evaluating enhancer-target gene prediction methods.** *Genome Biol* 2020, **21:**17.

12.	Zhang Y, Koneva LA, Virani S, Arthur AE, Virani A, Hall PB, Warden CD, Carey TE, Chepeha DB, Prince ME, et al: **Subtypes of HPV-Positive Head and Neck Cancers Are Associated with HPV Characteristics, Copy Number Alterations, PIK3CA Mutation, and Pathway Signatures.** *Clin Cancer Res* 2016, **22:**4735-4745.

13.	Jung HS, Uenishi G, Park MA, Liu P, Suknuntha K, Raymond M, Choi YJ, Thomson JA, Ong IM, Slukvin, II: **SOX17 integrates HOXA and arterial programs in hemogenic endothelium to drive definitive lympho-myeloid hematopoiesis.** *Cell Rep* 2021, **34:**108758.

**Second round of review**

**Reviewer 1**

The authors have addressed all my prior concerns. I have only a few very minor correction suggestions:

Line 286. The reported number of TFs appears intertwined with the citations: “...cell type is ~55 [4 - 96], ...”
Line 730. In the “Data availability” section, the Chip-Enrich website link cannot be opened.

**Reviewer 2**

The authors have done substantial changes and additions in this revised submission and all but one of my major concerns from the previous review have been addressed in the revision.

The concern that is still germane (from the previous review) is that the authors examined a large number of possible schemes for enhancer-gene assignment and found some that do better and some that do worse than a simple baseline (nearest TSS), and this does not assure the reader that they should switch to one of the new EnTDefs in favor of the simpler baseline in their future research. Systematically exploring a large number of schemes and finding that about half of them perform better than a baseline is not convincing evidence that the “smart” schemes are a better idea than the simple baseline. In response, the authors note: “Our goal of this study is to prioritize the generated EnTDefs which leveraged the state-of-the-art knowledge of enhancer-gene assignments across a large number of cell types, and ultimately select one that can provide the best biological interpretation of regulome data. Therefore, the number of EnTDefs that showed better or worse than the baseline is not of concern, …”. But this goal is predicated on the assumption that a more complex scheme and/or one that “leveraged the state-of-the-art knowledge” is desirable over the simpler baseline. I do not subscribe to this assumption a priori, and believe that complexity and/or use of prior knowledge comes at the peril of over-fitting, and its value has to be objectively demonstrated. I had originally interpreted the goal of this work differently: to try and show that there is an alternative to the “nearest TSS” baseline that provides substantial benefits in terms of accuracy (over the commonly used baseline), and hence the readers should use that alternative enhancer-gene assignment scheme in the future. To show this, the authors needed to demonstrate that the best EnTDef from their evaluations is going to retain its value on unseen benchmarks as well. The previous submission had not convinced me that the *extent* of improvement (of the best EnTDef over the nearest TSS baseline) is generalizable and robust to the choices of evaluation scheme and benchmark. The new results added, especially revised Figure 4A, takes an important step towards demonstrating generalizability. However, the results presented are confusing to this reviewer. What would have been convincing is a simple figure showing that F1 or AUPR scores in a cross-chromosome CV (similar to the AUPR scores reported in the BENGI paper) are clearly better for the top EnTDef (where “top” is defined without having seen the BENGI benchmark) compared to the baseline. What is shown instead is that the relative performance of the top 10, middle 10 and bottom 10 EnTDefs generalizes, by and large, to the BENGI benchmark (Figures 4A,B) and to other external benchmarks. What the reader wants to know, in the simplest manner possible, is *how much better* the top EnTDef does in assigning enhancers to genes, in a standard cross-validation type setting. No clear answer is provided for this question, even though Figure 4 is attempting to address it indirectly. I hope the authors can provide a more clear answer to this question.

**Authors’ response to reviewers**

**Reviewer #1:**  The authors have addressed all my prior concerns. I have only a few very minor correction suggestions:

Line 286. The reported number of TFs appears intertwined with the citations: “...cell type is ~55 [4 - 96], ...”

*Response to reviewer:*

We thank the reviewer for pointing out the confusing text. The numbers in the brackets are the minimum and maximum numbers of the TFs tested in each cell types. We have modified the text to be: “(the average number of TFs tested in each cell type is ~55, ranging from 4 to 96, see **Supplementary Table S2**)”

 Line 730. In the “Data availability” section, the Chip-Enrich website link cannot be opened.

*Response to reviewer:*

We are sorry for the inconvenience, there was an issue with using http:// versus https://.

**Reviewer #2:** The authors have done substantial changes and additions in this revised submission and all but one of my major concerns from the previous review have been addressed in the revision.

The concern that is still germane (from the previous review) is that the authors examined a large number of possible schemes for enhancer-gene assignment and found some that do better and some that do worse than a simple baseline (nearest TSS), and this does not assure the reader that they should switch to one of the new EnTDefs in favor of the simpler baseline in their future research. Systematically exploring a large number of schemes and finding that about half of them perform better than a baseline is not convincing evidence that the “smart” schemes are a better idea than the simple baseline. In response, the authors note: “Our goal of this study is to prioritize the generated EnTDefs which leveraged the state-of-the-art knowledge of enhancer-gene assignments across a large number of cell types, and ultimately select one that can provide the best biological interpretation of regulome data. Therefore, the number of EnTDefs that showed better or worse than the baseline is not of concern, …”. But this goal is predicated on the assumption that a more complex scheme and/or one that “leveraged the state-of-the-art knowledge” is desirable over the simpler baseline. I do not subscribe to this assumption a priori, and believe that complexity and/or use of prior knowledge comes at the peril of over-fitting, and its value has to be objectively demonstrated. I had originally interpreted the goal of this work differently: to try and show that there is an alternative to the “nearest TSS” baseline that provides substantial benefits in terms of accuracy (over the commonly used baseline), and hence the readers should use that alternative enhancer-gene assignment scheme in the future. To show this, the authors needed to demonstrate that the best EnTDef from their evaluations is going to retain its value on unseen benchmarks as well. The previous submission had not convinced me that the *extent* of improvement (of the best EnTDef over the nearest TSSbaseline) is generalizable and robust to the choices of evaluation scheme and benchmark. The new results added, especially revised Figure 4A, takes an important step towards demonstrating generalizability. However, the results presented are confusing to this reviewer. What would have been convincing is a simple figure showing that F1 or AUPR scores in a cross-chromosome CV (similar to the AUPR scores reported in the BENGI paper) are clearly better for the top EnTDef (where “top” is defined without having seen the BENGI benchmark) compared to the baseline. What is shown instead is that the relative performance of the top 10, middle 10 and bottom 10 EnTDefs generalizes, by and large, to the BENGI benchmark (Figures 4A,B) and to other external benchmarks. What the reader wants to know, in the simplest manner possible, is *how much better* the top EnTDef does in assigning enhancers to genes, in a standard cross-validation type setting. No clear answer is provided for this question, even though Figure 4 is attempting to address it indirectly. I hope the authors can provide a more clear answer to this question.

*Response to reviewer:*

We thank Reviewer 2 for clarifying their remaining concern, and we agree that to move to a more complex method of assigning genomic regions to target genes, significant statistical and biological improvement is required. Furthermore, we agree that this improvement needs to be shown using *1) multiple different benchmarks and 2)* *independent data that confirms the apparent improvement is not just due to ‘over-fitting’ or by chance*. We now better understand the basis of the concern and believe that we have now added the analyses and figures to address it. The reviewer is understandably still concerned that **1)** We did not present our top ranked EnTDef compared to BENGI in a clear, easily understandable way (i.e. the percent positive minus percent negative overlapping is an indirect and not easily interpretable measure). **2)** We did not include the baseline ‘nearest TSS’ approach in the comparison (Figure 4A), so it was not possible to compare our top EnTDef to ‘nearest TSS’ using the BENGI benchmark. **3)** we didn’t clearly show the extent of improvement over the baseline of ‘nearest TSS’ with independent data for either benchmark, that is, with gene set enrichment (GSE) testing and with BENGI. We now do both, since a major goal of this study is to improve gene set level interpretation of genome-wide regulatory data, as the manuscript title indicates.

First, we note that all of our EnTDefs and rankings were produced without using any information from BENGI (e.g. without having seen the BENGI benchmark first). By directly comparing our top/middle/bottom EnTDefs and the ‘nearest TSS’ with the cross-chromosome CV BENGI benchmark datasets, we now report the F1-scores and also separately sensitivity and specificity for them instead of the previous Figure 4A. (Note that unlike the methods compared in Fig 4 of the BENGI manuscript, our EnTDefs are not machine learning based, and do not have predictive scores for each enhancer-target assignment. Therefore, we are not able to perform the evaluation based on the AUPR scores in cross validation (CV) as the reviewer suggested. In other words, instead of being able to calculate an entire ROC curve or precision-recall curve when comparing to BENGI, we obtain only a single value for a sensitivity/specificity or precision/recall plot for each EnTDef and BENGI dataset.) Detailed methods for calculating this sensitivity, specificity and precision are provided at the end of our response for your reference and in the Supplementary Methods.

We examined the F1-scores of the top/middle/bottom EnTDefs, as well as the top EnTDef_plus5kb and baseline methods (‘nearest TSS’ and 5kb_outside). Since the ‘nearest TSS’ method assigns genome-wide genomic locations to their nearest genes, whereas our EnTDefs only assign distal regions (>5kb of a TSS) to their target genes, we also included the EnTDef_plus5kb (i.e. EnTDef + 5kb locus definition) which is more directly comparable to ‘nearest TSS’. As shown in the **updated Figure 4A**, the average F1-scores of the top10 EnTDef were significantly higher than ‘nearest TSS’ in both BENGI datasets (EnTDef vs nearest_TSS ANOVA test in BENGI with fixed positive/negative ratio [1:4]: p value = 2.74×10^-104^, and in BENGI with natural positive/negative ratio [much more negative than positive pairs]: p value = 5.48×10^-3^), although the extent of the increase became smaller in the BENGI natural ratio dataset. We note that for all BENGI datasets and results, the data with “ambiguous pairs” removed were shown for simplification, since the results and all plots were nearly identical with versus without them (see an example in **Response to reviewers Figure 1**).

It is worth noting that the ranks of our top/middle/bottom EnTdefs and baseline methods based on the BENGI-derived F1-scores vs. those based on our original GSE testing-derived F1-scores were highly correlated (**updated Figure 4B**), indicating general concordance between the two main benchmarks used. Furthermore, our single top EnTDef.top_plus5kb ranked by GSE was also the top ranked by BENGI (also updated Figure 4B). However, a difference is that BENGI consistently ranked methods *without* the 5kb promoter regions (EnTDef.top and 5kb_outside) higher than the ones with the 5kb promoter regions (EnTDef.top_plus5kb and nearest_tss), whereas the GSE benchmark did the opposite.

In addition to the F1 scores, we separately examined sensitivity and specificity to understand *how* our top EnTDefs were better than ‘nearest TSS’. The overall sensitivity of the top1 EnTDef showed a 91% increase compared to that of the ‘nearest TSS’ method (0.61 vs 0.32), while the specificity and precision decreased by ~20% and ~14%, respectively (**Response to reviewers Table 1**). This is a beneficial trade-off, especially for applications such as GSE testing where sensitivity (comprehensiveness) is very important, and it is fairly robust to moderate decreases in specificity. Nevertheless, it is an important piece of information beyond what the F1-score illustrates. The average sensitivity of the top10 EnTDefs and EnTDefs_plus5kb were significantly increased as compared to ‘nearest TSS’ and ‘>5kb’ in all BENGI subsets, while the specificity showed a small decrease (**Supplementary Figure S6A**). The same trend can be observed in individual EnTDefs plotted as sensitivity vs (1-specificity) (**Supplementary Figure S6B)** as is done for a ROC curve**.**

Although in this revision we updated the evaluation metric for benchmarking EnTDefs using BENGI data (i.e. using sensitivity/specificity/F1-score instead of the % positive minus % negative in the previous manuscript), we still examined how our EnTDefs performed compared to ‘nearest TSS’ using the previous metric for completeness. As shown in the **Response to reviewers Figure 2**, our top10 EnTDefs with or without plus 5kb significantly outperformed ‘nearest TSS’ (Wilcoxon signed-rank test, EnTDef.top10_pus5kb vs nearest_tss: p=1.781×10^-8^; EnTDef.top10 vs nearest_tss: p= 1.368×10^-10^).

For the second part of this revision, we compared our top EnTDefs to ‘nearest TSS’ using GSE testing on a completely independent set of ChIP-seq experiments. That is, we used data that are both from completely different, non-overlapping transcription factors (TFs) and completely different, non-overlapping cell types. Thus, our original ranking of EnTDefs was not aware of any of these TFs or cell types. The new datasets include 31 ENCODE ChIP-seq experiments of 9 cell lines and 14 transcription factors (TFs) which all passed quality controls according to the Cistrome project (<http://cistrome.org/db/#/about>). The ROC and precision-recall (PR) curves were generated for each ChIP-seq dataset when comparing the significant Gene Ontology biological process (GOBP) terms with the assigned ones for the tested TF (same as previous Methods: “Evaluation of enhancer-target gene definitions”) at a series of GSE p value cutoffs, and the AUPRC and AUROC were calculated. As compared to the baseline methods, our top10 EnTDefs with or without plus 5kb locus definition had both higher overall AUPRC and AUROC across the 31 ChIP-seq datasets (**Supplementary Figure S3C and Supplementary Table S7**). This is independent evidence showing the outperformance of our EnTDef compared to the commonly used ‘nearest TSS’ method, illustrating the robustness of our top EnTDefs across a broad range of datasets.

Overall, given the remarkable increase in recall (sensitivity) with only a moderate decrease in specificity and precision when compared with the ‘nearest TSS’ approach, the selected top1 EnTDef is much more beneficial to identify distal regulatory events and thus will be more informative to support hypothesis generation and gene set enrichment testing for regulome studies. Accordingly, we added in the discussion: “For those using an enhancer-target gene links database where it is more important to limit false positive links than to identify true ones (i.e. specificity is much more important than sensitivity), then our top EnTDef approach is likely not optimal. However, for gene set enrichment, identifying likely enhancer-target links that will be validated by experiment, or other situations where it is important to have high sensitivity, our top EnTDef has been well optimized and clearly outperforms the nearest TSS approach”.

**Updated Figure 4.** (A) Violin plots of average F1-scores of different types of EnTDefs (top 10 EnTDefs with 5kb locus definition [EnTDef.top_plus5kb], top10 EnTDefs (distal regions >5kb from TSS only), middle 10 EnTDefs [ranked at 732 - 741] and bottom 10 EnTDefs) in BENGI datasets with “ambiguous pairs” removed (“BENGI_fixedRatio”: the positive and negative pairs in 1:4 ratio; “BENGI_naturalRatio”: originally generated negative pairs). The values of the GSE F1 ranked top1 EnTDefs with or without 5kb locus definition are annotated by a red star, and the values of baseline locus definitions (nearest TSS [nearest_tss] and >5kb [5kb_outside]) are annotated by the dashed lines (red: nearest TSS, blue: >5kb). ANOVA test was performed between the top10 EnTDefs and nearest TSS in “BENGI_fixedRatio”: p value = 2.74×10^-104^, and in “BENGI_naturalRatio”: p value = 5.48×10^-3^. (B) The ranking comparison of the top 10 with 5kb (red), top 10 (dark red), middle 10 (ranked at 732 - 741) (coral), bottom 10 (blue) EnTDefs, nearest TSS (orange) and >5kb (magenta) based on GSE testing-derived F1-score (x-axis) vs. BENGI benchmarking-derived F1-score (y-axis). The Pearson’s correlation (R), regression equation and the p values are shown for each type of BENGI benchmark datasets.

**Response to reviewer Table 1.** Average sensitivity, specificity and precision of nearest TSS and top1 EnTDef as compared to the BENGI datasets

**Supplementary Figure S6.** Different views of sensitivity and specificity of the selected EnTDefs and baseline locus definitions as compared to the BENGI dataset. (A) Violin plots of average sensitivity and specificity of EnTDefs (top 10 EnTDefs.plus5kb [EnTDef.top_plus5kb], top10 EnTDefs, middle 10 EnTDefs and bottom 10 EnTDefs) in each type of BENGI datasets. The values of the top1 EnTDef are annotated by a red star and the values of baseline locus definitions (nearest TSS [nearest_tss] and >5kb [5kb_outside]) were annotated by the dashed lines (red: nearest TSS, blue: >5kb). ANOVA test was performed between top10 EnTDefs and nearest TSS: sensitivity in BENGI with fixed positive/negative ratio (BENGI_fixedRatio): p value = 3.92×10^-226^; sensitivity in BENGI with natural positive/negative ratio (BENGI_naturalRatio): p value =8.56×10^-237^; specificity in BENGI_fixedRatio: p value = 2.28×10^-156^; specificity in BENGI_naturalRatio: p value = 4.48×10^-218^ (B) Scatter plots of overall false positive rate (1-specificity) vs. true positive rate (sensitivity) of each type of EnTDefs and baseline locus definitions in BENGI_fixedRatio and BENGI_naturalRatio dataset separately.

**Response to reviewers Figure 1.** Scatter plots of false positive rate (1-specificity) vs. true positive rate (sensitivity) of EnTDefs and baseline locus definitions in individual cross-chromosome cross-validation BENGI sub-datasets.

**Response to reviewers Figure 2.** Violin plots of average %pos minus %neg of EnTDefs (top 10 EnTDefs.plus5kb [EnTDef.top_plus5kb], top10 EnTDefs, middle 10 EnTDefs and bottom 10 EnTDefs) in each type of BENGI datasets. The values of baseline locus definitions (nearest TSS [nearest_tss] and >5kb [5kb_outside]) were annotated by the dashed lines (red: nearest TSS, blue: >5kb). ANOVA test was performed between top10 EnTDefs and nearest TSS, in BENGI with fixed positive/negative ratio (BENGI_fixedRatio): p value = 6.59×10^-157^; BENGI with natural positive/negative ratio (BENGI_naturalRatio): p value = 3.97×10^-153^.

**Supplementary Figure S3C.** Scatter plot of average AUPRC (aura under the precision-recall curve) vs AUROC (aura under the receiver operating characteristic curve) of the top 10 EnTDef, top 10 EnTDef_plus5kb and baseline locus definitions (nearest_tss and 5kb_outside) in the gene set enrichment (GSE) testing on the 31 independent ChIP-seq datasets from 14 transcription factors in 9 cell lines, which were completely different from the TFs and cell lines used in the EnTDef ranking analysis.

*Methods for benchmarking EnTDefs using BENGI dataset:*

Firstly, we identified the overlapped enhancer regions between our EnTDefs and BENGI dataset, and then called true positive (TP), false positive (FP), true negative (TN) and false negative (FN) for each of the BENGI pairs according to the following criteria: *i)* TP call when BENGI pair was true (1) and the assigned gene was also assigned to the overlapped enhancers in the comparative EnTDef or baseline methods; *ii)* FP call when BENGI pair was false (0) and the assigned gene was also assigned to the overlapped enhancers in the comparative EnTDef or baseline methods; *iii)* TN call when BENGI pair was false (0) and the assigned gene was not assigned to the overlapped enhancers in the comparative EnTDef or baseline methods; and *iv)* FN call when BENGI pair was true (1) and the assigned gene was not assigned to the overlapped enhancers in the comparative EnTDef or baseline methods. Using the number of the BENGI pairs in each of the four categories (TP/FP/TN/FN), we calculated and reported the average true positive rate (i.e. sensitivity or recall, TF/[TF+FN]), false positive rate (i.e. 1-specificity, 1-TN/[TN+FP]) and precision (positive predictive value, TP/[TP+FP]) scores.
